# YAP/TAZ activation mediates PQ-induced lung fibrosis by sustaining senescent pulmonary epithelial cells

**DOI:** 10.1186/s12931-024-02832-z

**Published:** 2024-05-18

**Authors:** Youjia Yu, Chunyan Chu, Kang Wang, Yan Li, Zhengsheng Mao, Li Hu, Jie Wang, Yanfang Yu, Hao Sun, Feng Chen

**Affiliations:** 1 Department of Forensic Medicine, Nanjing Medical University, 101 Longmian Avenue, Jiangning District, Nanjing, 211166 Jiangsu China; 2 Department of Pathology, School of Medicine, Zhongda Hospital, Southeast University, Nanjing, 210009 Jiangsu China; 3 Biomedical publications center, Nanjing Medical University, Nanjing, 211166 Jiangsu China; 4 Department of Emergency, Affiliated Hospital of Medical School, Nanjing Drum Tower Hospital, Nanjing University, Nanjing, 210008 Jiangsu China; 5 The Key Laboratory of Modern Toxicology of Ministry of Education, Nanjing Medical University, Nanjing, 211166 Jiangsu China; 6 Key Laboratory of Targeted Intervention of Cardiovascular Disease, Collaborative Innovation Center for Cardiovascular Disease Translational Medicine, Nanjing Medical University, Nanjing, 211166 Jiangsu China; 7 Wuxi People’s Hospital Affiliated with Nanjing Medical University, Wuxi, 214023 Jiangsu China

**Keywords:** Paraquat, Poisoning, Pulmonary fibrosis, Cellular senescence, Pulmonary epithelial cell, YAP/TAZ

## Abstract

Paraquat (PQ) is a widely used herbicide and a common cause of poisoning that leads to pulmonary fibrosis with a high mortality rate. However, the underlying mechanisms of PQ-induced pulmonary fibrosis and whether pulmonary epithelial cell senescence is involved in the process remain elusive. In this study, PQ-induced pulmonary epithelial cell senescence and Hippo-YAP/TAZ activation were observed in both C57BL/6 mice and human epithelial cells. PQ-induced senescent pulmonary epithelial cells promoted lung fibroblast transformation through secreting senescence-associated secretory phenotype (SASP) factors. Yap/Taz knockdown in mice lungs significantly decreased the expression of downstream profibrotic protein Ctgf and senescent markers p16 and p21, and alleviated PQ-induced pulmonary fibrosis. Interfering YAP/TAZ in senescent human pulmonary epithelial cells resulted in decreased expression of the anti-apoptosis protein survivin and elevated level of apoptosis. In conclusion, our findings reveal a novel mechanism by which the involvement of Hippo-YAP/TAZ activation in pulmonary epithelial cell senescence mediates the pathogenesis of PQ-induced pulmonary fibrosis, thereby offering novel insights and potential targets for the clinical management of PQ poisoning as well as providing the mechanistic insight of the involvement of Yap/Taz activation in cell senescence in pulmonary fibrosis and its related pulmonary disorders. The YIN YANG balance between cell senescence and apoptosis is important to maintain the homeostasis of the lung, the disruption of which will lead to disease.

**Supplementary Information:**

The online version contains supplementary material available at 10.1186/s12931-024-02832-z.

## Introduction

Paraquat (PQ; N, N-dimethyl-4, 4′-bipyridinium dichloride) is a widely used non-selective contact herbicide and is linked to a series of human diseases such as pulmonary fibrosis and Parkinson’s disease [1, 2]. PQ has been registered and used in over 120 developed and developing countries throughout the world [3]. Although PQ has been banned in Europe and recently in China, it is still in use in the United States and many Asian countries [4]. Mortality rates of PQ intoxication, especially in self-poisoned patients, remain greater than 50% due to a lack of efficient therapeutic measurements [5, 6], which makes PQ intoxication a severe global health problem, particularly in developing countries. PQ causes multi-organ injury, particularly characterized by pulmonary fibrosis, due to its accumulation in the lung by specific polyamine uptake [3]. Clinically, death occurs either at the early phase of intoxication due to interstitial inflammation and severe pneumonia followed by respiratory failure, or after progression to pulmonary fibrosis within a few weeks [7]. Understanding the underlying mechanism of PQ-induced pulmonary fibrosis is of great importance for clinical resuscitation.

Given that PQ causes cellular toxicity predominately by excessive free radicals generation and mainly targets the lung, mechanisms related to oxidative stress, inflammatory response, and fibrosis become major research focuses [4]. Cellular senescence is a state of stable replicative arrest triggered by various stressors, including oxidative stress, DNA damage, and proteome instability [8]. PQ is known to induce cellular senescence in the brain and is used to establish Parkinson’s disease (PD) model [2]. Senescent cells affect tissue functions and structure via secreting bioactive molecules including inflammatory cytokines, chemokines, and metalloproteinases, characterized as senescence-associated secretory phenotype (SASP) [9]. SASP factors have been reported to participate in the pathogenesis of idiopathic pulmonary fibrosis (IPF) by promoting inflammation and epithelial-to-mesenchymal transition (EMT) processes [10, 11]. Our previous studies have demonstrated that PQ intoxication induces cell cycle arrest in human pulmonary epithelial cells, reduces anti-oxidative metabolites, and upregulates the SASP factor IL-6 in murine models [12–14]. However, it remains elusive if pulmonary epithelial cell senescence contributes to PQ-induced pulmonary fibrosis.

Signaling pathways involved in PQ-induced lung injury have been studied in order to reveal the toxic mechanisms and to develop potential therapeutic strategies. The Hippo pathway is a well-characterized pathway that regulates organ growth and oncogenesis. Yes-associated protein 1 (YAP) and WW domain-containing transcription factor (WWTR1 or TAZ) are major Hippo downstream effectors of canonical Hippo pathway [15]. The expression and activation of YAP can be rapidly regulated by biological processes such as oxidative stress [16, 17], energy homeostasis [18], and mechanical pressure [19]. Two recent studies reported the increased expression of YAP and its downstream profibrotic factor connective tissue growth factor (Ctgf) in rodent PQ poisoning models, indicating the potential role of YAP in PQ-induced lung injury [17, 20]. Moreover, in IPF models, YAP/TAZ is shown to contribute to pulmonary fibrosis and alveolar regeneration [21]. So far, the role of the Hippo-YAP/TAZ pathway in PQ-induced lung fibrosis is still unknown.

In the current study, we aimed to investigate the role and mechanism of pulmonary epithelial cell senescence in PQ-induced pulmonary fibrosis. Initially, both in vivo and in vitro studies revealed the presence of cellular senescence and activation of the Hippo-YAP/TAZ pathway in pulmonary mesenchyme and bronchial epithelial cells after PQ treatment. Further investigations demonstrated that these senescent epithelial cells could stimulate the transformation of lung fibroblasts. Additionally, YAP/TAZ inhibition was found to prevent cell senescence and induce cell apoptosis, which protected against PQ-induced pulmonary fibrosis. Our findings uncover a novel molecular mechanism for PQ-induced lung senescence and fibrosis, which may provide new insights and potential targets for the clinical management of PQ-poisoned patients.

## Materials and methods

### Chemicals and reagents

Paraquat (CAS: 75365-73-0) and dihydroethidium (DHE) (CAS: 104821-25-2) were purchased from Sigma-Aldrich (St. Louis, MO, United States). Verteporfin (VP) (CAS: 129497-78-5) was provided by Absin, China. The primary antibodies included anti-p16^Ink4a^ (for immunofluorescence: ab211542, 1:100, Abcam, UK; for Western blotting: for human: 10883-1, 1:1000, Proteintech, China; for mouse: sc-1661, 1:1000, Santa Cruz Technology, TX, United States), anti-p21^Cip1/Waf1^ (sc-6246, 1:1000, Santa Cruz Technology), anti-β-actin (4970, 1:8000, CST, MA, United States), anti-Ki-67 (AG8471, 1:100, Beyotime, China), anti-α-SMA (19245, 1:200, CST), anti-pYAP (Ser127) (13008S, 1:1000, CST), anti-YAP (12395S, 1:1000 for Western blotting and 1:400 for immunohistochemisty, CST; 13584-1-AP, 1:200 for immunofluorescence, Proteintech), anti-pTAZ (Ser89) (59971S, 1:1000, CST), anti-TAZ (83669S, 1:1000 for Western blotting and 1:200 for immunofluorescence, CST), anti-Survivin (10508-1-ap, 1:1000, Proteintech), anti-Bcl-2 (BS1511, 1:1000, BioWorld, China), anti-Bax (50599-2-Ig, 1:1000, Proteintech), anti-Fn1 (sc-8422, 1:1000 for Western blotting, Santa Cruz Technology; ab45688, 1: 500 for immunofluorescence, Abcam), anti-AQP5 (20334-1-AP, 1:100, Proteintech), Sfptc (10774-1-AP, 1:200, Proteintech), anti-Cyk19 (GB12197, 1:500) and anti-Ctgf (23936-1-AP, 1:1000, Proteintech). ELISA kits for human IL-6, IL-1α, and IL-8, as well as mouse Il-6, Il-1α, and Il-8 were obtained from Yifeixue Biotech, China. WST-1 kit and SA-β-gal staining kit were offered by Beyotime. The terminal deoxynucleotidyl transferase-mediated dUTP nick-end labeling (TUNEL) kit was provided by Vazyme, China. siRNAs were synthesized by RiboBio, China. siRNA sequences were listed in Table S1. Adeno-associated virus 5 (AAV5) was constructed by HanBio, China.

### Animal experiments

This study was designed following the guidelines of the Institute for Laboratory Animal Research of Nanjing Medical University. All protocols were approved by the Animal Care and Ethical Committee of Nanjing Medical University (IACUC-2006015, IACUC-2107031). Male and female mice of 8-week-old with body weight (BW) of 20–25 g (C57BL/6 mice, Oriental Bio Service Inc., Nanjing, China) were maintained under a constant environmental condition with a temperature of 23℃ ± 2℃, humidity of (55 ± 5)%, and 12:12 h light/dark cycle in the Animal Research Center of Nanjing Medical University with free access to food and water before and after all procedures. Mice were randomly divided into PBS control (ctrl) and PQ group (*N* = 8 for each group). Mice were instilled with 50 µL PBS In the ctrl group or 0.02 mg PQ in the PQ group, and sacrificed after 14 days. In the YAP/TAZ knockdown experiment, mice were intratracheally infected with 5 × 10^10^ PFU of AAV-Ctrl, AAV-shYap, AAV-shTaz, or both AAV-shYap and AAV-shTaz and instilled with 0.02 mg PQ at day 21 post-infection. PBS was instilled at day 0 and day 21 in the PBS control group (*N* = 6 for each group).

### Cell culture and treatments

A549, 16HBE, human lung fibroblast (HLF), HEK293T, and L929 cells were preserved in our lab and cultured in Dulbecco’s modified eagle’s medium (DMEM) (KeyGEN BioTECH, China) with 10% fetal bovine serum (FBS) (Lonsa, United States). Cells were incubated under 5% CO_2_ at 37℃. The senescent lung epithelial cell model was established as follows: A549 cells were 1:4 and 16HBE cells were 1:10 passaged to the culture plates and immediately added with 200 µM PQ. Cells were changed with fresh medium (without PQ) after 24 h and continuously cultured for another 6 days. For lentivirus (LV) preparation, HEK293T cells were co-transfected with pRRE, pRSV, pMD2.G, and PLKO.1 which was constructed with shYAP, shTAZ, or shCtrl sequences. The supernatant containing LV was harvested at 48 h and 72 h after transfection and stored at − 80℃ until use.

### Histology and immunostaining

Tissues were embedded with paraffin or OCT and cut into 5-µm thick slides. Paraffin slides were stained with hematoxylin and eosin (HE) or Masson’s trichrome staining as manufacturer’s instruction. Frozen slides or cells growing on coverslides were stained by SA-β-gal or incubated with primary antibodies at 4℃ overnight, and then incubated with fluorescent secondary antibodies and DAPI. Slides were observed under a fluorescence microscope (BX53, Olympus, Japan).

### Western blotting

Lung tissues and cells were lysed with RIPA buffer as previously described. An equal amount of protein samples were separated by 10% or 12% SDS-PAGE and transferred to the PVDF membrane. After blocking with 5% skim milk, membranes were incubated with primary antibodies at 4℃ overnight, washed with TBS-T, and then incubated with secondary antibodies at room temperature for 1 h. Band visualization was performed with the enhanced chemiluminescence kit (ECL, Thermo-Fisher, United States), and the quantification was done by ImageJ FIJI software.

### qRT-PCR

Total RNA was extracted from lung tissues and cells with TRIzol (Yifeixue Biotech). Approximately 1 µg of total RNA was used for reverse transcription with a first-strand cDNA synthesis kit (abm, China). Quantitative real-time PCR (qRT-PCR) was performed using BrightGreen 2× qPCR MasterMix (abm) with a three-step protocol by the CFX Optics Module (Bio-Rad, Singapore). Measured targets and primer sequences are listed in Table S2.

### Measurement of cytockines

Blood was obtained from mice eyeballs and serum was obtained by centrifugation at 8,000 g for 15 min. Cell supernatant was collected and filtered with a 0.22 μm filter to remove cell debris. The serum samples and supernatant were stored at -80 °C until use. The levels of cytokines were measured with ELISA kits according to the manufacturer’s manual with POLARstar Omega (BMG Labtech, Germany).

### TUNEL assay

Cells growing on the coverslide were washed twice with PBS after 4% paraformaldehyde fixation. TUNEL assay was performed according to the manufacturer’s instructions. The apoptotic cells were observed with a fluorescence microscope (Olympus) as indicated by their distinct green color.

### Transcriptome analysis

Raw sequencing data of mouse lung transcriptome were derived from our earlier experiments [22]. RPKM calculations were performed using the limma and countToFPKM packages in R. The heatmaps were generated by applying heatmap.2 package based on the RPKM.

### Statistical analysis

Data analysis was performed using GraphPad Prism version 6.01 (GraphPad Software, United States). Data were shown as mean ± SEM. Data were analyzed by Student’s *t*-test between 2 groups, and one-way analysis of variance (ANOVA) followed by Dunnett’s correction was used for comparisons among multiple groups. Statistical significance was set at *P* < 0.05.

## Results

### PQ treatment induces pulmonary cellular senescence in mice

**Fig. 1:**
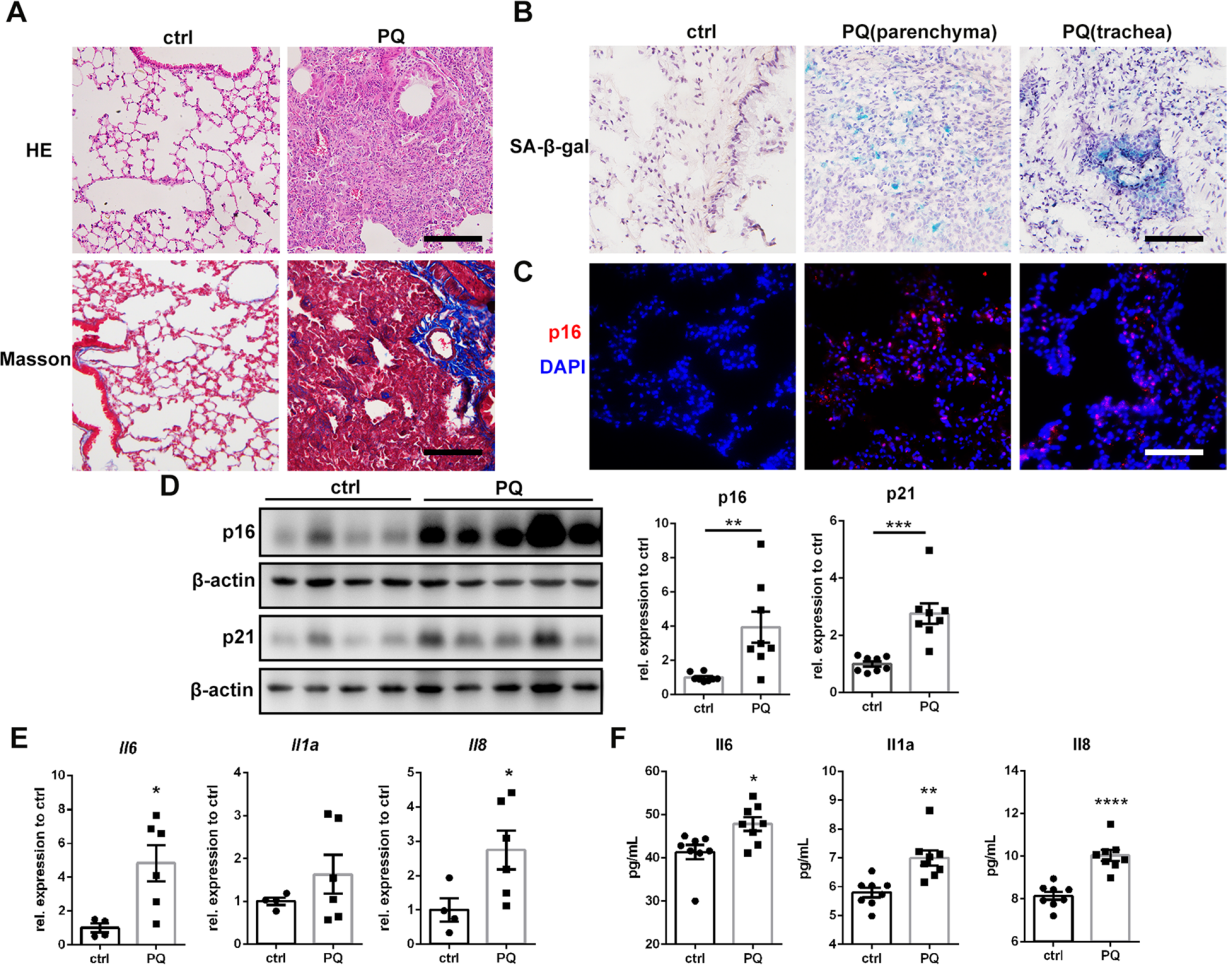
Paraquat treatment induces pulmonary cellular senescence in mice: **A** HE staining and Masson’s trichrome staining of representative lung sections from control and PQ group (original magnification 100×, scale bar = 400 μm), **B** SA-β-gal staining of parenchyma and trachea in lung tissues (original magnification 200×, scale bar = 200 μm), **C** representative images of immunoflourescence staining for p16 (red) and DAPI (blue) in lung sections (original magnification 200×, scale bar = 200 μm), **D** total lung protein was assessed for p16 and p21, with β-actin as loading control (*N* = 8) by Western blotting, **E** relative mRNA levels of SASP markers *Il6*, *Il1a* and *Il8* compared to *Actb* in total lung tissues (*N* = 4 for ctrl group, *N* = 6 for PQ group) were analyzed by qRT-PCR, **F** serum levels of SASP markers Il-6, Il-1α and Il-8 (*N* = 8) were tested by ELISA assay. Values are shown as mean **±** SEM. Data were analyzed by Student’s *t* test. * *P* < 0.05, ** *P* < 0.01, *** *P* < 0.005, **** *P* < 0.001

Mouse lungs instilled with PQ for 2 weeks were stained with H&E and Masson’s trichrome staining. Obvious consolidation of the lung, fibroblast accumulation, decreased alveolar volume, and peri-bronchia collagen deposition were observed (Fig. 1A), indicating the successful establishment of PQ-induced lung injury. Senescence markers SA-β-gal staining and p16 immunofluorescence staining showed that the senescent cells were deposited in pulmonary mesenchyme and bronchia, which were mostly alveolar epithelial cells and bronchial epithelial cells (Fig. 1B, C). Protein expressions of p16 and p21 in total lung tissues were both significantly elevated with PQ treatment (Fig. 1D). SASP factors were measured using qRT-PCR. *Il6* and *Il8* mRNA levels were significantly increased in PQ lung tissues (Fig. 1E). Elevated serum levels of Il-6, Il-1α, and Il-8 after PQ treatment were validated by ELISA assay (Fig. 1F). These results indicate that PQ could induce pulmonary cellular senescence.

### PQ induces human pulmonary epithelial cell senescence in vitro

**Fig. 2:**
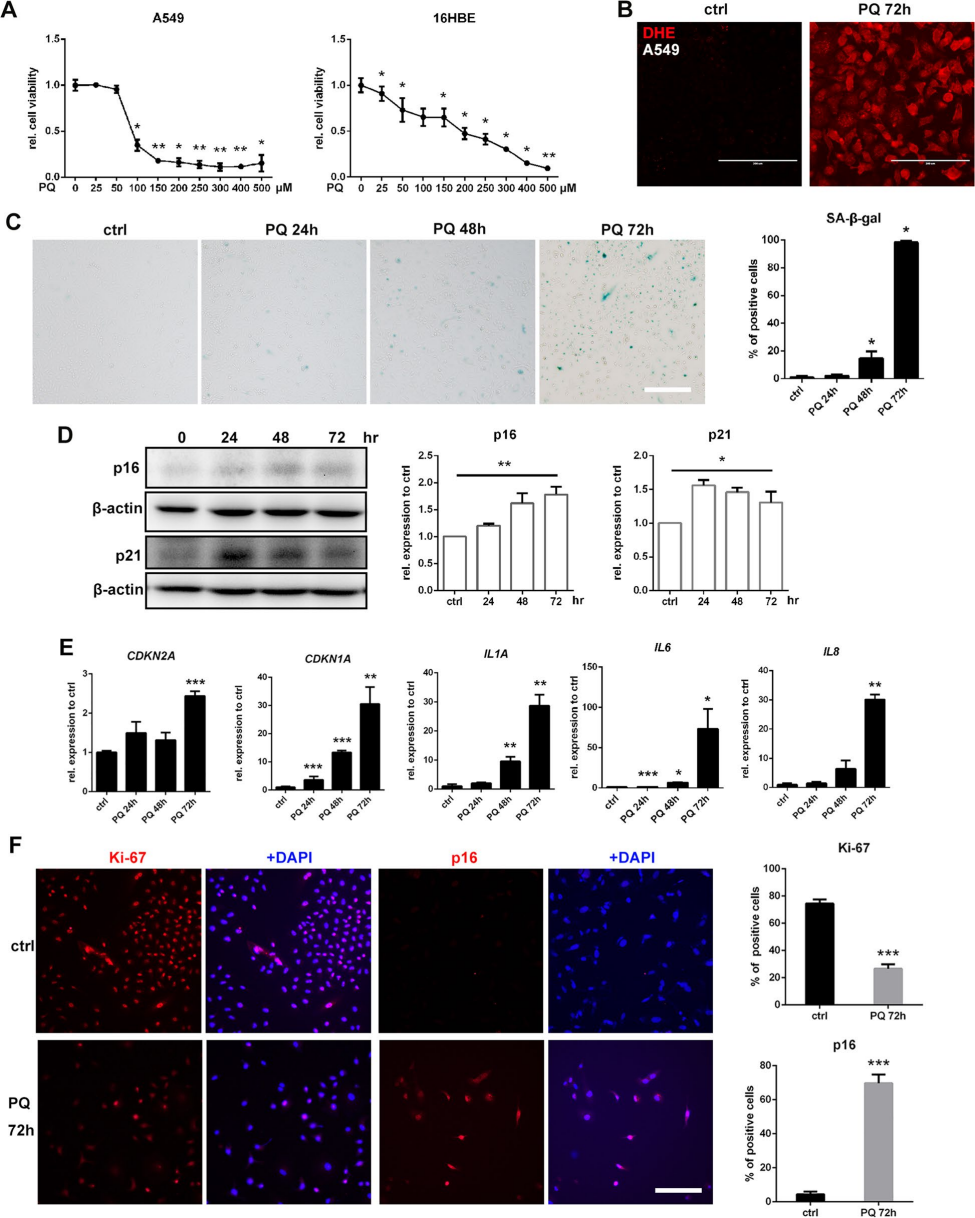
Paraquat induces pulmonary epithelial cell senescence in vitro: **A** WST-1 analysis shows dose-dependent decreases of A549 and 16HBE cells after PQ exposure for 72 h, **B** ROS content in A549 cells after treated with 80 µM PQ for 72 h was analyzed with DHE staining (red) (original magnification 200×, scale bar = 200 μm), **C** A549 cells were stained with SA-β-gal after treated with 80 µM PQ for 24 h, 48 h and 72 h (original magnification 200×, scale bar = 200 μm), and quantification of positive cells, **D** A549 cells treated with 80 µM PQ for 24 h, 48 h and 72 h were harvested and assessed for p16 and p21, with β-actin as loading control by Western blotting, **E** relative mRNA levels of *CDKN2A* (p16), *CDKN1A* (p21), and SASP markers *IL6*, *IL1a* and *IL8* compared to *ACTB* in A549 cells treated with 80 µM PQ for 24 h, 48 h and 72 h were analyzed by qRT-PCR. **F** immunoflourescence staining of A549 cells treated with 80 µM PQ for 72 h for Ki-67 (red), p16 (red) and DAPI (blue) (original magnification 200×, scale bar = 200 μm), and quantification of positive cells. All statistical data were from three independent experiments. Values are shown as mean ± SEM. Data were analyzed by Student’s *t* test between 2 groups, and one-way ANOVA with the Dunnett’s correction was used for comparisons among multiple groups. * *P* < 0.05, ** *P* < 0.01, *** *P* < 0.005

Human type II alveolar epithelial cell-like cell A549 and human bronchial epithelial cell 16HBE were employed to study the effect of PQ on pulmonary epithelial cell senescence *in vitro.* WST-1 assays were performed to evaluate the cytotoxicity of PQ on these cells and the data revealed that PQ led to a dose-dependent reduction in cell viability (Fig. 2A). IC_50_ for A549 was 80 µM and for 16HBE was 200 µM. IC_50_ for both cell lines were adopted for further studies. DHE staining showed elevated ROS production in A549 and 16HBE cells with exposure to PQ for 72 h (Fig. 2B and S1A). SA-β-gal staining showed an obvious increase in the number of positive cells, which were larger in size, after 48 h of PQ treatment (Fig. 2C). Western blotting and qRT-PCR showed the elevation of both protein and mRNA levels of p16 (*CDKN2A*) and p21 (*CDKN1A*), and increased mRNA levels of *IL1A, IL6*, and *IL8* after 24 to 48 h of PQ treatment (Fig. 2D, E, and S1B, C). Immunofluorescence staining indicated decreased Ki-67 expression of A549 cells, which was not obvious in 16HBE cells, while p16 was positively stained in most cells of both cell lines after 72 h of PQ treatment (Fig. 2F and S1D). To exclude the possibility that cell death also induced senescence in neibouring cells, a lower concentration (50µM) of PQ was used to treating both cell lines. Increased number of SA-β-gal-positive cells (Fig S1E) and elevated protein levels of p16 and p21 were also observed after exposed to PQ for 72 h (Fig S1F). The above results demonstrated that pulmonary epithelial cells exhibit a significant senescence phenotype within 72 h of PQ exposure in vitro.

### Senescent epithelial cells promote lung fibroblast transformation via secreting SASP factors

**Fig. 3:**
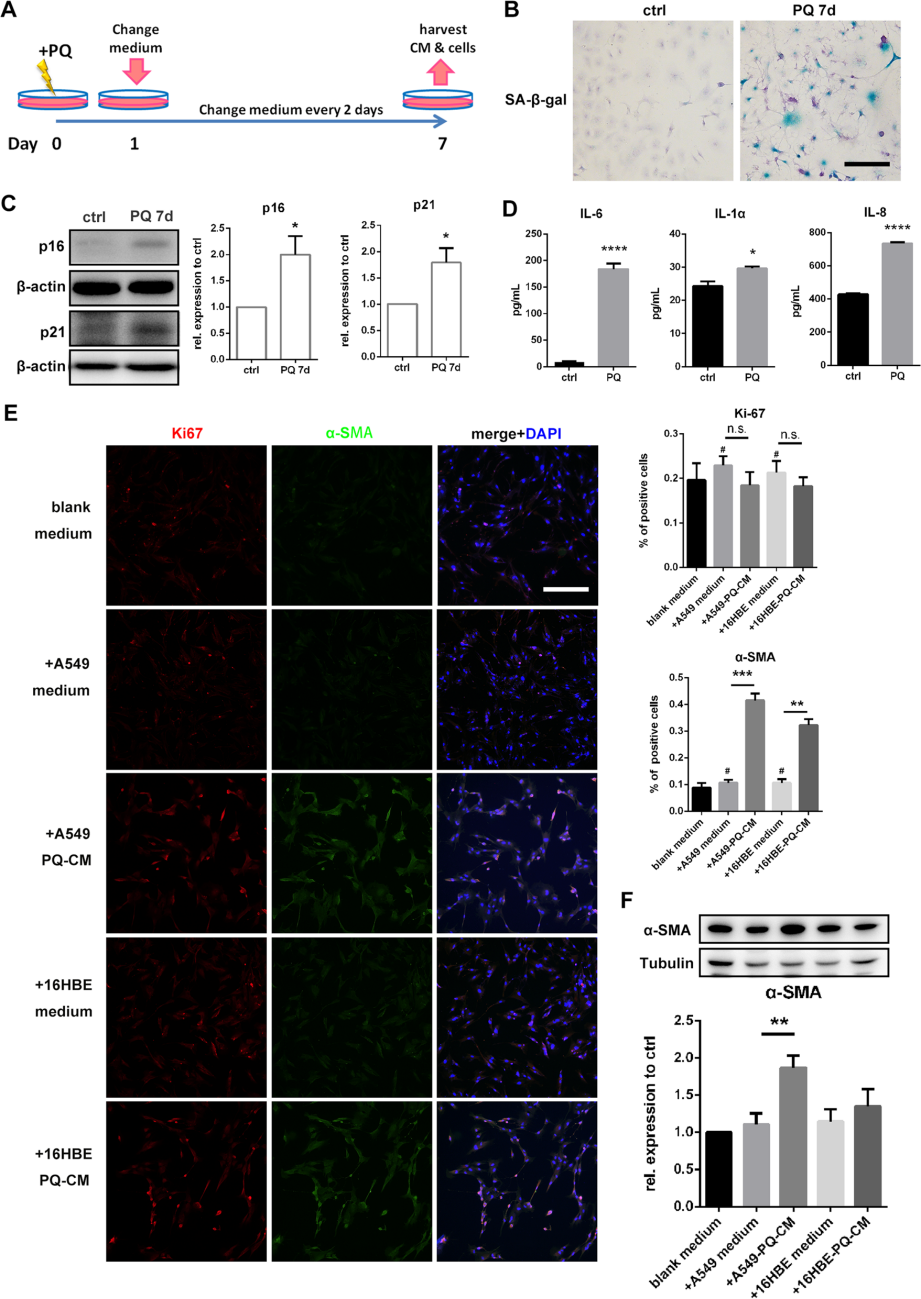
Senescent lung epithelial cells promote lung fibroblast transformation via secreting SASP factors: **A** Schematic illustration of the establishment of senescent lung epithelial cell model. A549 and 16HBE cells were exposed to 200 µM PQ for 24 h, and then changed with fresh medium for continuous 6-day culture. Cells and supernatant (PQ-conditional medium, PQ-CM) were harvested at the 7th day; medium incubated with untreated cells for 48 h were harvested as control medium, **B** A549 cells were stained with SA-β-gal after exposed to 200 µM PQ for 24 h and continuously cultured for 6 days (original magnification 200×, scale bar = 200 μm), **C** A549 cells harvested at the 7th day were assessed for p16 and p21, with β-actin as loading control by Western blotting, **D** SASP markers IL-6, IL-1α and IL-8 in the supernatant were tested by ELISA assay, **E** immunoflourescence staining of HLF cells after 72 h incubation with A549 and 16HBE CM for Ki-67 (red), α-SMA (green) and DAPI (blue) (original magnification 200×, scale bar = 200 μm), and quantification of positive cells, **F** HLF cells incubated with CM for 72 h were assessed for α-SMA, with Tubulin as loading control by Western blotting. All statistical data were from three independent experiments. Values are shown as mean **±** SEM. Data were analyzed by Student’s *t* test. * *P* < 0.05, ** *P* < 0.01, *** *P* < 0.005, **** *P* < 0.001, n.s. no statistical significance, # no statistical significance compared to blank medium group

To further study the pathological functions of senescent pulmonary epithelial cells in PQ-induced lung injury, senescent cell models of A549 and 16HBE were established as described (Fig. 3A). On the 7th day after exposure to PQ, cells were harvested for senescence phenotype detection, and the supernatant was preserved as PQ-conditional medium (PQ-CM). All cells were positively stained with SA-β-gal (Fig. 3B). P16 and p21 proteins were significantly increased as shown by Western blotting (Fig. 3C and S2A). Elevation of IL-6, IL-1α, and IL-8 levels in the supernatant was demonstrated by ELISA assay (Fig. 3D and S2B). By using immunofluorescence staining and Western blotting, an obvious increase of myofibroblast marker α-SMA was shown in HLF after incubating with A549 and 16HBE PQ-CM for 72 h comparing with medium from routine cell culture. However, no significant increase in the number of Ki-67-positive cells was observed (Fig. 3E and F). Our results indicate that senescent pulmonary epithelial cells promote lung fibroblast transformation through secreting SASP factors, but do not affect fibroblast proliferation.

### YAP and TAZ are activated during PQ-induced pulmonary cellular senescence *in vivo* and *in vitro*

**Fig. 4:**
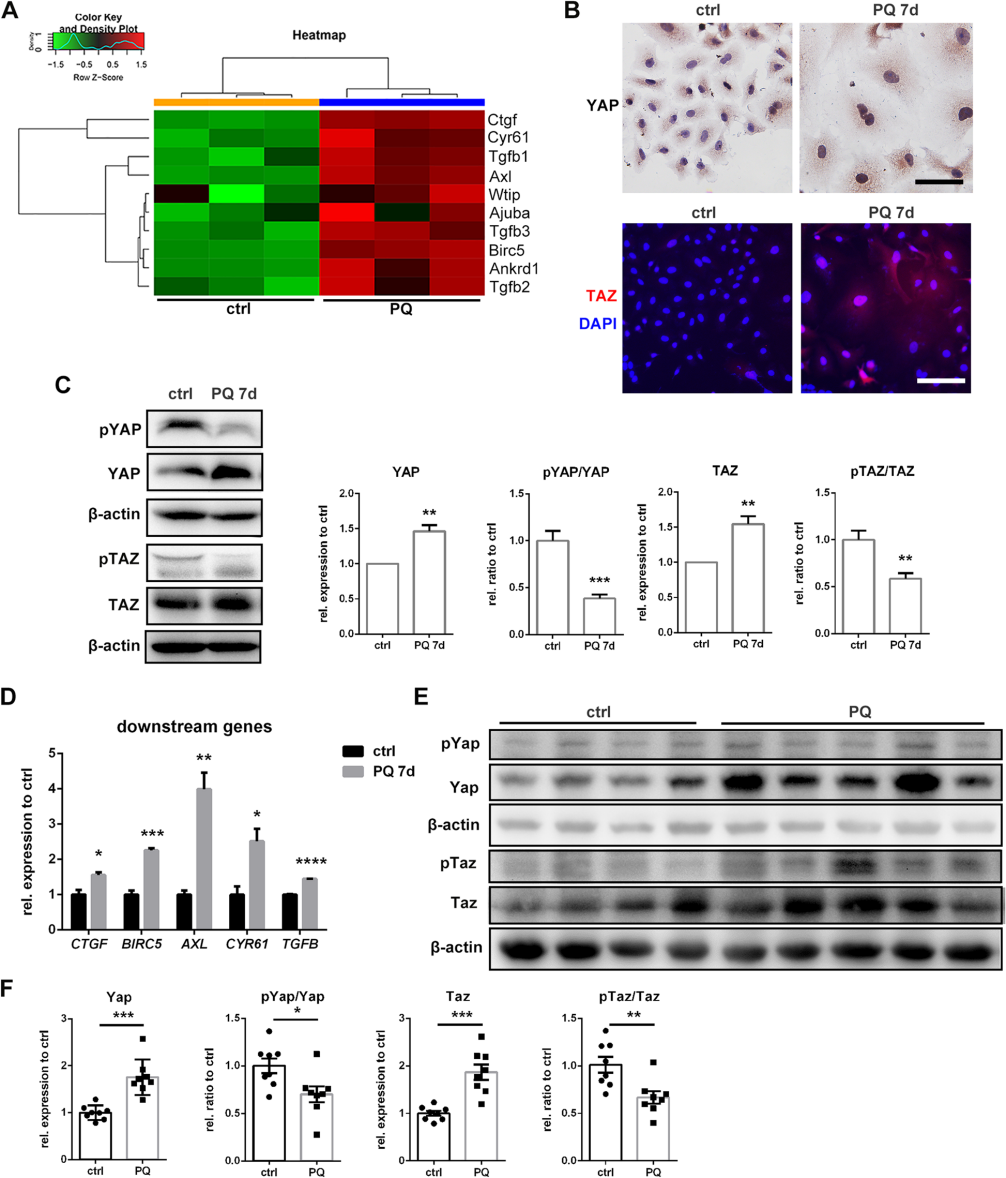
YAP and TAZ are activated during paraquat induced pulmonary cellular senescence in vivo and in vitro: **A** Heatmap of Yap/Taz downstream genes in lungs of PQ treated mice and control mice from RNAseq data, **B** immunohistochemistry staining for YAP and immunoflourescence staining for TAZ (red) and DAPI (blue) in senescent A549 cells (original magnification 400×, scale bar = 100 μm), **C** senescent A549 cells were assessed for pYAP, YAP, pTAZ, and TAZ, with β-actin as loading control by Western blotting, **D** relative mRNA levels of YAP/TAZ downstream genes compared to *ACTB* in senescent A549 cells were analyzed by qRT-PCR, **E**, **F** total lung protein was assessed for pYap, Yap, pTaz and Taz, with β-actin as loading control (*N* = 8) by Western blotting. All statistical data for were from three independent experiments in vitro. Values are shown as mean **±** SEM. Data were analyzed by Student’s *t* test. * *P* < 0.05, ** *P* < 0.01, *** *P* < 0.005, **** *P* < 0.001

**Fig. 5:**
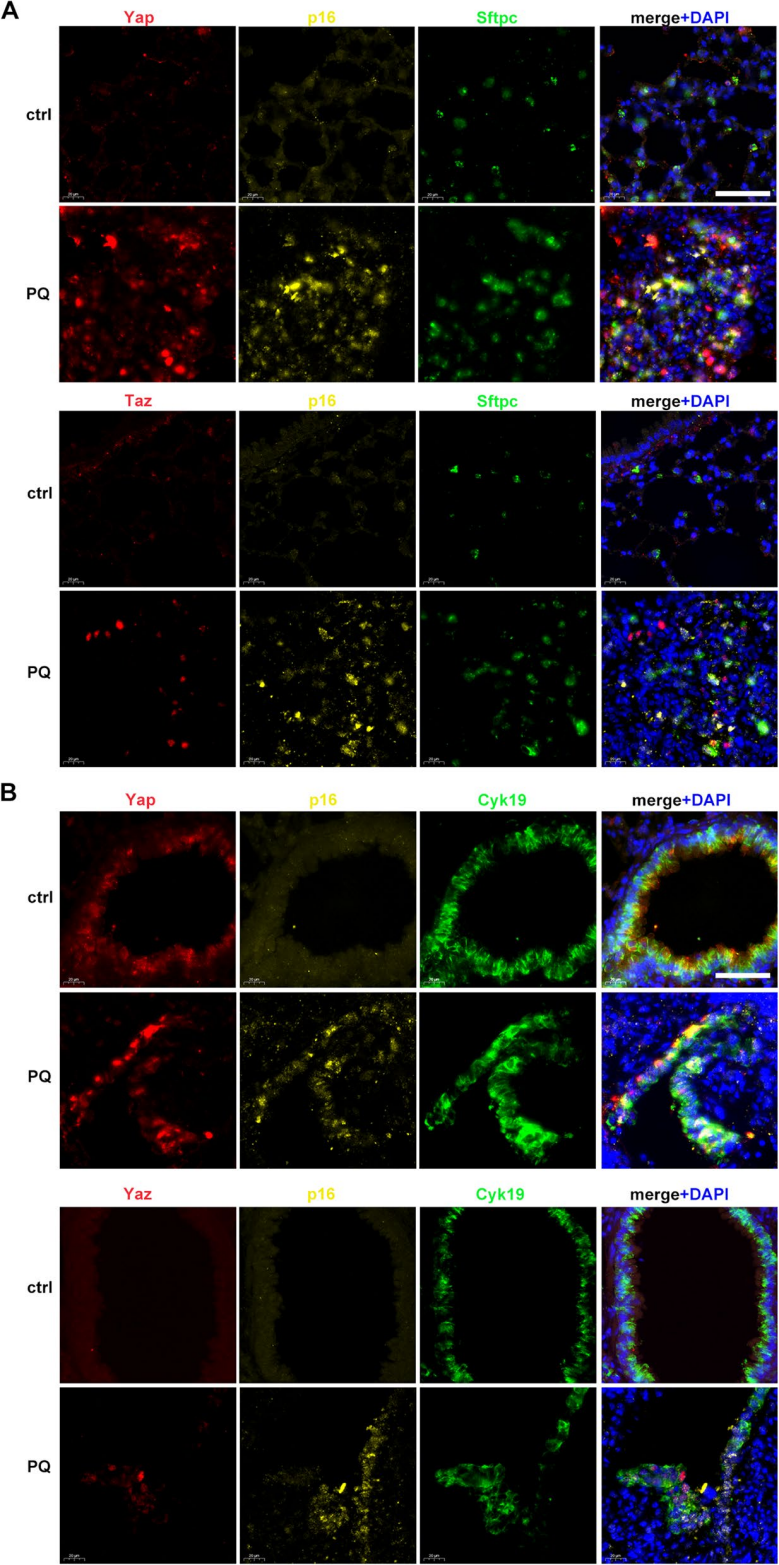
Co-locolization of Yap/Taz and p16 is observed in type II alveolar epithelial cells and bronchial epithelial cells: **A** representative images of immunoflourescence staining for Yap or Taz (red), p16 (yellow), type II alveolar epithelial cell marker Sftpc (green) and DAPI (blue) in lung sections of PQ group and control group (original magnification 400×, scale bar = 100 μm), **B** representative images of immunoflourescence staining for Yap or Taz (red), p16 (yellow), bronchial epithelial cell marker Cytokeratin 19 (Cyk19, green) and DAPI (blue) in lung sections of control and PQ  groups (original magnification 400×, scale bar = 100 μm)

To elucidate the underlying mechanisms of PQ-induced pulmonary senescence, we analyzed our previous transcriptome data [22] and found that expressions of Yap/Taz downstream genes were significantly elevated with PQ treatment (Fig. 4A). Hippo-YAP/TAZ signaling is closely related to cell proliferation and apoptosis, and might also contribute to lung fibrosis [21, 23]. YAP and TAZ are activated by dephosphorylation in cytoplasm and translocation into the nucleus, resulting in transcriptional activation of downstream genes [24]. Thus, we hypothesized that Yap/Taz activation might be involved in PQ-induced lung senescence and fibrosis. In the current senescent cell model, increased expression and nucleus accumulation of YAP and TAZ were observed by immunostaining, while they were predominantly expressed in cytoplasm in regularly cultured cells (Fig. 4B and S2C). Western blotting showed the increased expression of total YAP and TAZ, and decreased levels of phosphorylation (Fig. 4C and S2D). Increased mRNA levels of representative YAP/TAZ downstream genes, including *CTGF*, *BIRC5*, *AXL*, *CYR61*, and *TGFB*, were validated in PQ-induced senescent cells (Fig. 4D). Increased expression of total Yap and Taz and decreased levels of Yap/Taz phosphorylation were also observed in PQ-treated mice (Fig. 4E-F). Immunostaining showed clear nucleus co-localization of Yap/Taz and p16 in type II alveolar epithelial cells and bronchial epithelial cells and nuclear localization of Yap/Taz in p16 positive cells after PQ treatment (Fig. 5A and B), suggesting Yap/Taz activation in senescent type II alveolar epithelial cells and bronchial epithelial cells. In addition, in fibrotic lung tissues, type I alveolar epithelial cells could not be observed (Fig S3) and Yap/Taz was not localized in fibroblasts (Fig S4). These results confirm the involvement of YAP and TAZ activation in PQ-induced pulmonary epithelial cell senescence.

### Yap and Taz inhibition abrogates PQ-induced pulmonary fibrosis by eliminating senescent cells

**Fig. 6:**
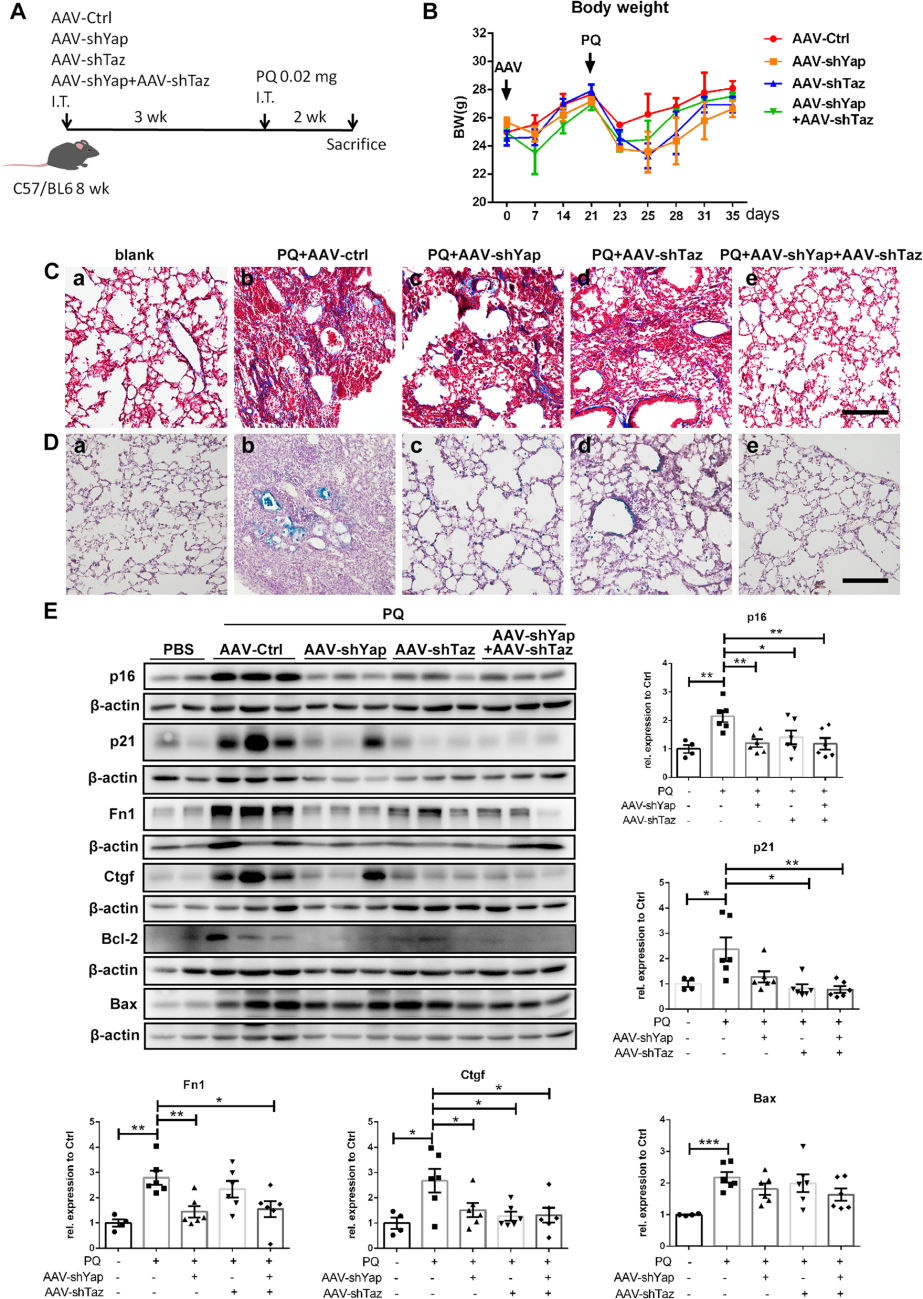
Yap and Taz knockdown abrogates paraquat-Induced pulmonary fibrosis in vivo: (**A**) Schematic illustration of the animal experiment. C57BL/6 mice were intratracheal infected with 5 × 10^10^ PFU of AAV-Ctrl, AAV-shYap, AAV-shTaz or both AAV-shYap and AAV-shTaz, and intratracheal instillated with 0.02 mg PQ at day 21 postinfection. PBS was intratracheal instillated at day 0 and day 21 as blank control, (**B**) body weight of mice in each group, (**C**) Masson’s trichrome staining (original magnification 100×, scale bar = 400 μm) and (**D**) SA-β-gal staining (original magnification 100×, scale bar = 400 μm)  of representative lung sections from each group: (a) blank control group, (b) AAV-Ctrl group, (c) AAV-shYap group, (d) AAV-shTaz group, (e) AAV-Yap + AAV-shTaz group, (**E**) total lung protein was assessed for p16, p21, Fn1, Ctgf, Bcl-2 and Bax, with β-actin as loading control by Western blotting (*N* = 4 for PBS group, *N* = 6 for other groups). All graphs are shown as mean ± SEM. Parametric variables were calculated using two-tailed Student’s *t* test between 2 groups. * *P* < 0.05, ** *P* < 0.01, *** *P* < 0.005

To further verify the functions of YAP and TAZ in PQ-induced lung injury, AAV5 was instilled to knock down *Yap* and *Taz* in mouse lungs. L929 cells were transfected with 3 siRNAs targeting *Yap* or 3 siRNAs targeting *Taz*, separately (Table S1), and their knockdown efficiencies were detected by Western blotting. Sequences of siYap-1 with the best knockdown efficiency of *Yap* and least interference of *Taz*, and siTaz-1 with the best knockdown efficiency of *Taz* and least interference of *Yap* were selected to be further constructed into AAV5 (AAV-shYap and AAV-shTaz) (Figure S5B). The knockdown efficiencies of AAV-shYap and AAV-shTaz were verified in the lung tissues of mice infected with AAVs for 3 weeks. Immunostainings and Western blotting showed satisifatory knockdown efficiencies of both AAVs (Figure S5C-E). Mice were infected with AAVs for 3 weeks and then instilled with PQ for 2 weeks (Fig. 6A). A mild decrease of BW after AAV infection and a rapid decrease of BW after PQ instillation were observed, indicating successful administration of AAV and PQ. There was no significant difference in BW among groups on the day of sacrifice (Fig. 6B). By Masson’s trichrome staining of lung slides in each group, we found decreased fibroblast accumulation and collagen deposition in lung tissues after the knockdown of *Yap*, *Taz*, or both *Yap* and *Taz* (Fig. 6C). SA-β-gal staining also showed decreased number of senescent cells in lung tissues from *Yap* and *Taz* knockdown mice (Fig. 6D), which was in accordance with the results of Western blotting. As shown in Fig. 6E, senescent markers p16 and p21, fibroblast marker Fn1, and profibrotic factor Ctgf, which was also a Yap/Taz downstream protein, were significantly decreased in the knockdown groups. However, expression of Bax was elevated after PQ treatment but was not significantly changed after *Yap* and *Taz* knockdown, indicating a minor effect on total apoptosis level of the lung tissues after interference of YAP and TAZ pathway. Together, these data show that lung-specific knockdown of *Yap* and *Taz* protects PQ-treated lungs from fibrosis by eliminating senescent cells.

### YAP and TAZ inhibition promotes PQ-induced senescent cells to apoptosis

**Fig. 7:**
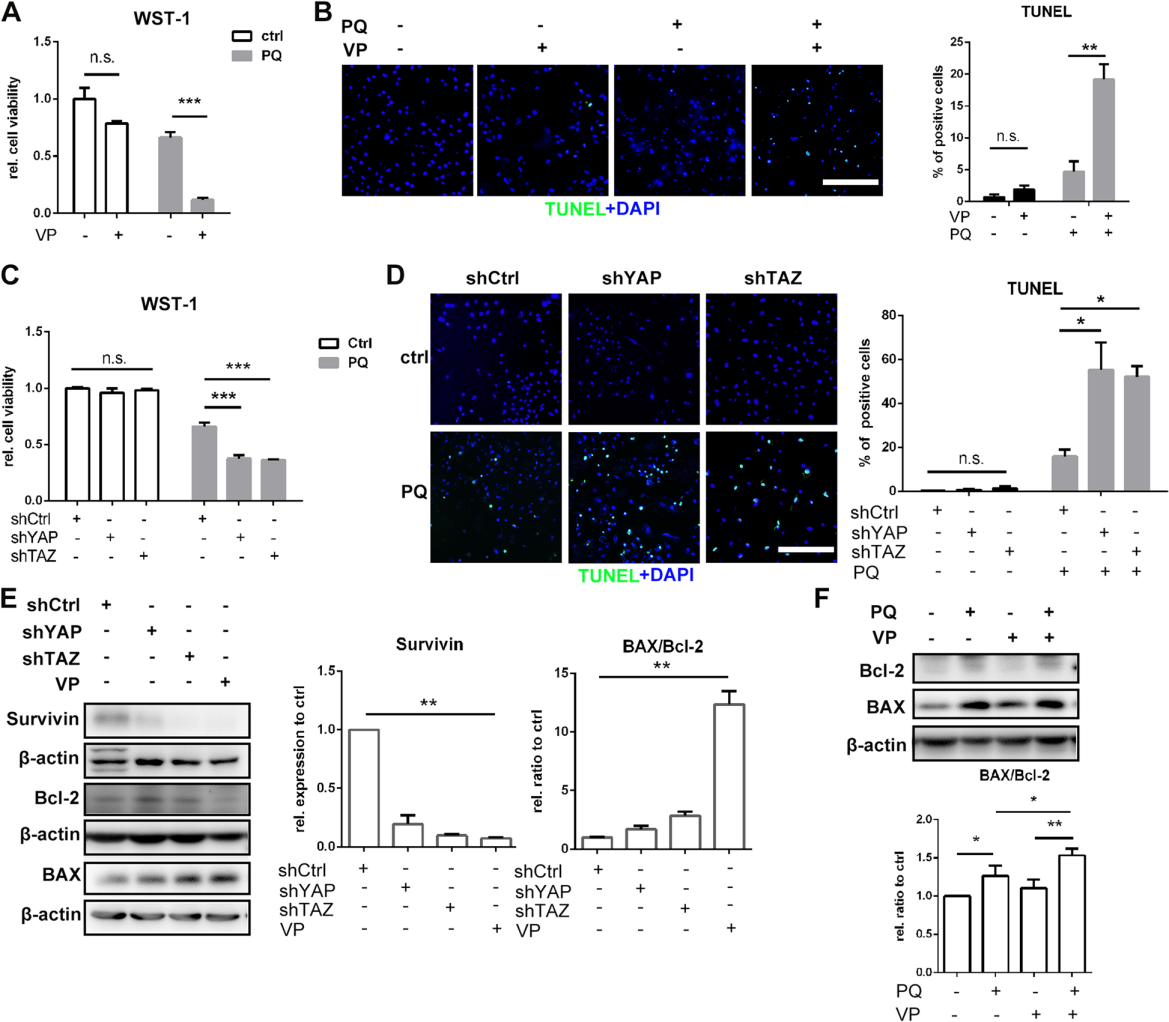
YAP and TAZ inhibition promotes paraquat induced senescent cells to apoptosis: A549 cells were treated with 2 µM verteporfin (VP) for 24 h and than treated with 200 µM PQ for 24 h, and detected after another 24 h. Cell viability was assessed by WST-1 analysis (**A**), apoptosis was detected by TUNEL staining (original magnification 200×, scale bar = 200 μm), and positive cells were quantified (**B**). A549 cells were infected with lentivirus interfering YAP (shYAP), TAZ (shTAZ) or control lentivirus seperately for 24 h and than treated with 200 µM PQ for 24 h, and detected after another 24 h. Cell viability was assessed by WST-1 analysis (**C**), apoptosis was detected by TUNEL staining (original magnification 200×, scale bar = 200 μm), and positive cells were quantified (**D**). **E** Senescent A549 cells treated with lentivirus or VP for 48 h were assessed for Survivin, Bcl-2 and BAX, with β-actin as loading control by Western blotting, (**F**) A549 cells treated with PQ for 48 h and VP for 24 h were assessed for Bcl-2 and BAX, with β-actin as loading control by Western blotting. All statistical data were from three independent experiments. Values are shown as mean ± SEM. Data were analyzed by Student’s *t* test between 2 groups, and one-way ANOVA with the Dunnett’s correction was used for comparisons among multiple groups. * *P* < 0.05, ** *P* < 0.01, *** *P* < 0.005, n.s.: no significance

To investigate the functions of YAP/TAZ in PQ-induced senescent pulmonary epithelial cells, verteporfin (VP), a YAP/TAZ inhibitor, was used to inhibit YAP/TAZ functions, and YAP and TAZ knockdown were performed by infection of cells with LV carrying sequences silencing YAP/TAZ. A549 cells were transfected with three siRNAs targeting *YAP* or three siRNAs targeting *TAZ* separately (Table S1), and the knockdown efficiencies were detected by Western blotting. Sequences of siYAP-2 with the best knockdown efficiency of YAP and least interference of TAZ, and siTAZ-1 with the best knockdown efficiency of TAZ and least interference of YAP were selected to be further constructed into LV (LV-shYAP and LV-shTAZ) (Figure S5A). We tried to establish the senescent cell model by pretreating cells with VP or LVs prior to PQ exposure, but all cells died within 72 h of PQ treatment. As shown in Fig. 7A-D and S6A-D, cells were pretreated with VP or LVs for 24 h and exposed to PQ for another 24 h, significantly reduced cell viability was observed by WST-1 in each treated group. TUNEL staining revealed that apoptosis occurred in the majority of the VP or LVs pretreated cells after PQ exposure. Next, we treated PQ-induced senescent cells with VP or LVs, and found that Survivin, a YAP/TAZ downstream gene that functioned as anti-apoptosis, was significantly suppressed by both VP and LVs. Likewise, the ratio of apoptosis markers BAX/Bcl-2 significantly increased after YAP/TAZ inhibition by VP or LVs (Fig. 7E). To investigate the short-term effect of VP on PQ treated cells, cells were treated with VP after exposure to 50 µM PQ for 48 h. Western blotting results showed that VP treatment could promote apoptosis indicated by the increased BAX/Bcl-2 ratio in PQ treated cells (Fig. 7F). The above results demonstrated the crucial role of YAP and TAZ in the survival of PQ-induced senescent pulmonary epithelial cells.

## Discussion

**Fig. 8:**
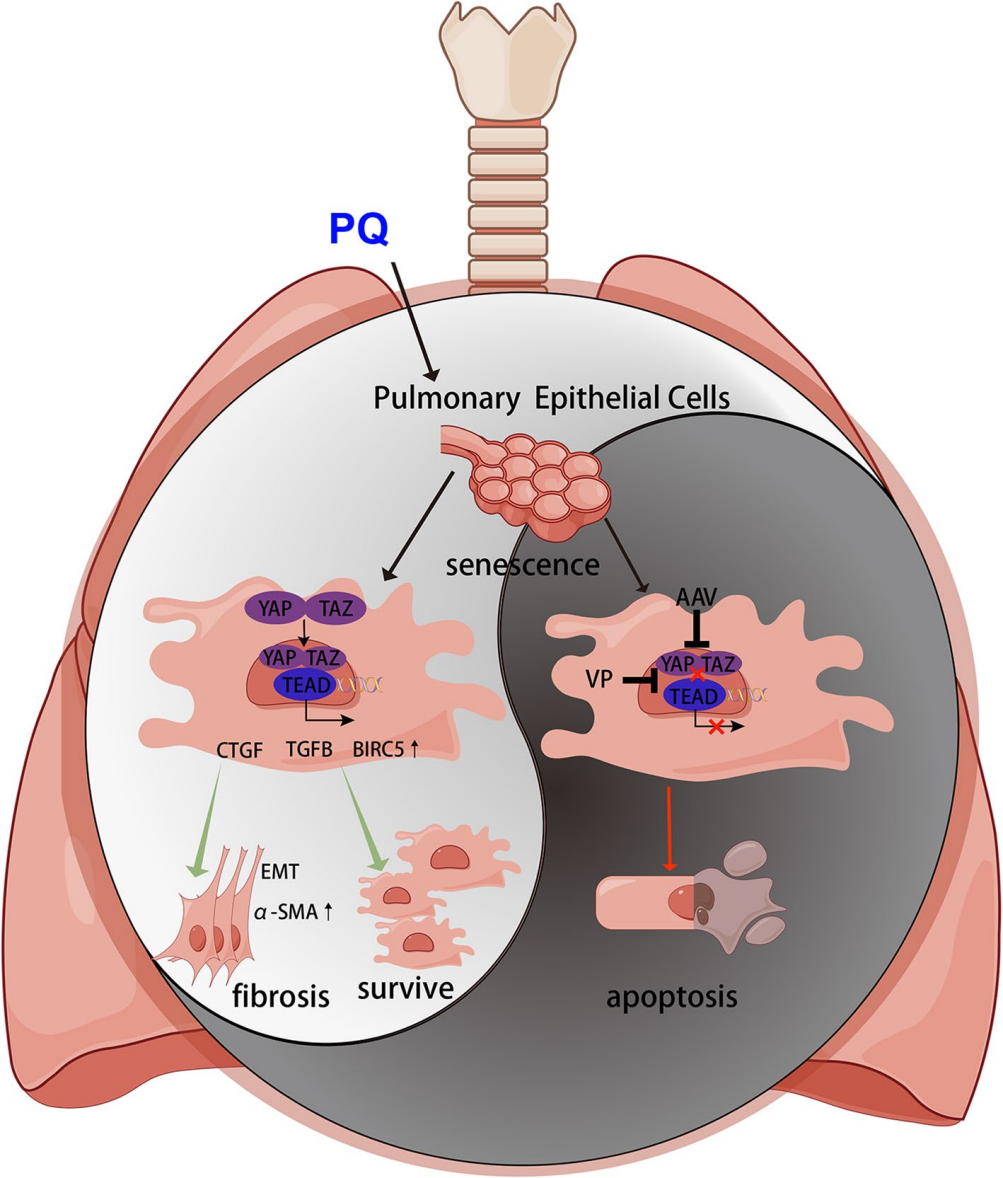
Overview of the present study

The present study revealed that pulmonary epithelial cell senescence and YAP/TAZ activation were the underlying mechanisms of PQ-induced pulmonary fibrosis. We employed human pulmonary epithelial cell lines to validate PQ-induced cellular senescence in vitro and established a senescent cell model to further disclose the functions of YAP/TAZ in senescent cell maintenance and pulmonary fibrosis. Our findings demonstrated that YAP/TAZ activation in pulmonary epithelial cells prevented PQ-induced senescent cells from apoptosis, which promoted pulmonary fibrosis via the continuous release of SASP factors. Importantly, we demonstrated that interference of YAP and TAZ in lungs could improve PQ-induced pulmonary fibrosis by eliminating senescent cells (Fig. 8).

The pathological features of the lungs in PQ intoxication include diffuse alveolar collapse, inflammatory cell infiltration, cell apoptosis, proliferation of bronchial epithelial cells, and excessive collagen deposition [3]. Current knowledge attributes these PQ-induced pulmonary injuries to redox cycle disruption [3], induction of cellular apoptosis [25], and subsequent initiation of inflammatory responses [13]. The DNA damage response (DDR) can be activated by intracellular ROS, transcriptionally activating the cyclin-dependent kinase (CDK) inhibitor p21^Cip1/Waf1^ and p16^Ink4a^, which antagonize CDKs to block cell cycle progression [8]. As revealed in our previous study, PQ induced cell cycle arrest and modulated the expressions of cyclinD1, p21, cyclinA2, and CDK2 ^12^, suggesting the initial growth arrest that might trigger cellular senescence via a p21-dependent mechanism. In the present study, we have validated the occurrence of cellular senescence, and observed that both p21 and p16 were elevated in lung tissues and cultured pulmonary epithelial cells after PQ treatment. The SASP is one of the key characteristics of senescent cells partially depending on the DDR signaling and ROS [26], manifested as upregulation of a prominent subset of genes that encode secreted cytokines and chemokines with proinflammatory properties, growth factors, and matrix remodeling proteases that modulate tissue structure and function [27]. A growing body of evidence implicates that cellular senescence contributes to fibrotic lung disease, such as IPF, chronic obstructive pulmonary disease, and bleomycin-induced lung fibrosis [10, 28, 29]. SASP of senescent alveolar epithelial cells acts as a viable origin of multiple signaling cascades that drive persistent fibroproliferative activation in bleomycin-induced pneumopathy [10, 30]. SASP factor IL-6 secreted by PQ-induced senescent human astrocytes is considered to be a crucial cytokine that causes neuropathy [31]. We have also reported in our previous work that PQ-induced pulmonary fibrosis through epigenetic upregulation of IL-6 ^13^. Here, we provided evidence of SASP in PQ-induced senescent pulmonary epithelial cells which promoted lung fibroblast transformation through secreting SASP factors. Our results revealed cellular senescence as one of the underlying mechanisms of PQ-induced pulmonary fibrosis.

To investigate the pathways involved in PQ-induced pulmonary epithelial cellular senescence, we analyzed our previous RNA-seq data and identified a cluster of YAP/TAZ downstream genes to be significantly elevated in lungs after PQ treatment, suggesting YAP/TAZ activation by PQ exposure. As downstream transcriptional regulators of the Hippo cascade, YAP and TAZ are highly related in function. Inhibitory phosphorylations of YAP and TAZ at Ser127 and Ser89 by LATS1/2 restrain YAP/TAZ functions in the cytoplasm, referred to as the ‘Hippo OFF’ state [19]. Once activated, YAP and TAZ translocate into the nucleus and interact with TEA domain (TEAD) transcription factors to trigger downstream gene transcription [32, 33]. Survivin, encoded by the *BIRC5* gene, is an important YAP/TAZ downstream gene belonging to the family of inhibitors of apoptosis proteins (IAPs) [34]. Survivin is crucial for suppressing apoptosis for its Baculovirus Inhibitor of the apoptosis repeat (BIR) domain, which is primarily responsible for inhibiting endogenous caspases [35]. Recently, YAP–TEAD pathway was revealed to influence senescent cell viability by a whole-genome CRISPR knockout screen technique [36]. Here, we showed that *BIRC5* mRNA was significantly upregulated after PQ treatment both in vivo and in vitro. Interfering YAP and TAZ could significantly decrease the expression of survivin in PQ-induced senescent pulmonary cells in vitro, which was coincident with cellular apoptosis. In vivo knockdown of YAP/TAZ in lungs decreases senescence markers p16 and p21 induced by PQ, demonstrating the elimination of senescent cells, possibly via inducing senescent cells to undergo cellular apoptosis.

Literatures show that YAP and TAZ are induced in the lungs of IPF patients, and sustain a pro-fibrotic transcriptional program including increased collagen, fibronectin deposition, and secretion of pro-fibrotic cytokines [37, 38]. The mechanism might be the transcriptional activation of pro-fibrotic downstream CTGF [33] by YAP/TAZ and crosstalk with TGF-β pathway [39]. PQ-induced pulmonary fibrosis displays similar pathological features with IPF [40]. Our results demonstrated YAP/TAZ activation and increased expression of CTGF and TGFB in PQ-treated lungs, thus, combined with the impact of YAP/TAZ on PQ-induced cellular senescence, we hypothesized that targeting YAP/TAZ could alleviate PQ-induced pulmonary fibrosis. By interfering YAP/TAZ in the murine model, the severity of pulmonary fibrosis was relieved after PQ treatment, together with the decrease of CTGF, suggesting the therapeutic potential of YAP/TAZ interference. VP is a benzoporphyrin derivative that inhibits the nuclear YAP/TAZ-TEAD interaction to suppress the transcriptional activity, which is an FDA-approved drug for the treatment of choroidal neovascularization and is commonly used as a YAP/TAZ inhibitor [41, 42]. A recent study showed that treating senescent cells with VP could selectively triggered apoptotic cell death in the organs of old mice and mice exhibiting doxorubicin-induced senescence [36]. Therefore, parallel to the AAV-knockdown experiments, we treated the PQ mice model with VP according to a previously reported method [43], but found that up to 70% of mice died within 10 days (data not shown). The reason could be the repetitive administration of high-dose VP and the possible interaction of PQ and VP. Therefore, though VP is a marketed drug, it might not become an ideal medication for PQ poisoning.

Pioneer study showed that removal of p16^Ink4a^-expressing cells could delay the onset of age-related pathologies and extend life span in mice [44], suggesting that reducing the burden of senescent cells could ameliorate age-related diseases. Senotherapeutics, including senolytics that selectively kills senescent cells and senomorphics that acts as SASP inhibitors, is a new class of antisenescence drugs to eliminate or delay the adverse effects of cellular senescence [45]. Senolytics were firstly reported in 2015 by Yi et al. [46]. Represented stratagies of senolytics include targeting proteins related to cell survival, such the BCL-2 protein family members, HSP90 and p53 [47]. IPF and COPD are most studied age-related lung diseases. It has been reported that high levels of the senescence markers p21 and p16 from patients with IPF and type II alveolar epithelial cell senescence is one of the key drivers of the occurance of pulmonary fibrosis [48]. Though senolytics drugs exert a positive effect on IPF mouse model [49], their efficancy and safety for IPF patients are waiting for verification by long-term preclinical trials [45]. A current phase I placebo-controlled pilot trial on feasibility and tolerability of dasatinib and quercetin (D + Q) therapy in IPF patients indicated a promising prospect of senolytics in clinic [50]. Until now, disputes on senolytics still exist for their the systemic toxicity and limited potency and cellular specificity in vivo, resticting their clinical application [51]. In this study, we provide a novel insight of senescent cell clearance strategy by silencing YAP/TAZ with AAVs in lung tissues. Considering numerous downstream genes of YAP/TAZ, further studies are needed to explore more accurate mechanisms and precise targets for senolytics.

In conclusion, our results uncover the linkage of cellular senescence and Hippo-YAP/TAZ pathway activation in the pathogenesis of PQ-induced pulmonary fibrosis. Targeting YAP/TAZ can alleviate pulmonary fibrosis through decreasing profibrotic downstream genes and elimination of senescent cells by inducing these cells to undergo apoptosis. Thus, the YIN YANG balance between cell senescence and apoptosis is important to maintain the homeostasis of the lung, the disruption of which may lead to disease. The present study provides new insights and potential targets for the clinical management of PQ poisoning.

### Supplementary Information

Supplementary Material 1.

Supplementary Material 2.

## Data Availability

No datasets were generated or analysed during the current study.

## Abbreviations

PQ: Paraquat
SASP: Senescence-associated secretory phenotype
IPF: Idiopathic pulmonary fibrosis
EMT: Epithelial-to-mesenchymal transition
YAP: Yes-associated protein 1
WWTR1: WW domain-containing transcription factor
Ctgf: Connective tissue growth factor
DHE: Dihydroethidium
VP: Verteporfin
TUNEL: Terminal deoxynucleotidyl transferase-mediated dUTP nick-end labeling
AAV5: Adeno-associated virus 5
BW: Body weight
DMEM: Dulbecco’s modified eagle’s medium
LV: Lentivirus
HE: Hematoxylin and eosin
ECL: Enhanced chemiluminescence qRT-PCR, quantitative real-time PCR ANOVA, analysis of variance
CM: Conditional medium
DDR: DNA damage response
CDK: Cyclin-dependent kinase
TEAD: TEA domain
IAPs: Inhibitors of apoptosis proteins
BIR: Baculovirus Inhibitor of the apoptosis repeat
