# Supplementary material for: YAP/TAZ activation mediates PQ-induced lung fibrosis by sustaining senescent pulmonary epithelial cells
Source: Respir Res. 2024 May 18;25:212. doi: 10.1186/s12931-024-02832-z (PMC11102259; doi:10.1186/s12931-024-02832-z)
Supplement: Supplementary file 1 — Supplementary Material 1. [file 12931_2024_2832_MOESM1_ESM.docx]

Supplementary data

**YAP/TAZ activation mediates PQ-induced lung fibrosis by sustaining senescent pulmonary epithelial cells**

**Youjia Yu ^a,#^, Chunyan Chu ^a,b,#^, Kang Wang ^a^, Yan Li ^a,c^, Zhengsheng Mao ^a^, Li Hu^a^, Jie Wang ^a^,Yanfang Yu ^a^, Hao Sun^d,^*, Feng Chen ^a,e,f^***

Table S1. SiRNA sequences

| Gene | siRNA-1 | siRNA-2 | siRNA-3 |
| --- | --- | --- | --- |
| Human *YAP* | GCTCATTCCTCTCCAGCTT | GCTGCCACCAAGCTAGATA | CCAGTTAAATGTTCACCAA |
| Human *TAZ* | CGATGAATCAGCCTCTGAA | GGACAAACACCCATGAACA | AGAGTCTGCTCTGAACAAA |
| Mouse *Yap* | CGAGATGAGAGCACAGACA | GCCAGTACTGATGCAGGTA | CCAACCAGCAGCAGCAAAT |
| Mouse *Taz* | CAGAATGACTTTAGAGAAT | CCTTAATCACATAGAGAAA | GAGGGCCCTATCATTCACG |

Table S2. Primer sequences.

| Gene | F(5’→3’) | R(3’→5’) |
| --- | --- | --- |
| *hACTB* | CCTGGGCATGGAGTCCTGTGG | CTGTGTTGGCGTACAGGTCTT |
| *hIL6* | TTCGGTCCAGTTGCCTTCTC | GAGGTGAGTGGCTGTCTGTG |
| *hIL1A* | GGTTGAGTTTAAGCCAATCCA | TGCTGACCTAGGCTTGATGA |
| *hIL8* | AGACAGCAGAGCACACAAGC | ATGGTTCCTTCCGGTGGT |
| *hCDKN2A* | TATCGCCAGGAATTGTTGCTG | CATCTATGCGGGCATGGTTACT |
| *hCDKN1A* | TGTCCGTCAGAACCCATGC | AAAGTCGAAGTTCCATCGCTC |
| *hCTGF* | CAGCATGGACGTTCGTCTG | AACCACGGTTTGGTCCTTGG |
| *hBIRC5* | AGGACCACCGCATCTCTACAT | AAGTCTGGCTCGTTCTCAGTG |
| *hAXL* | ATCAGCTTCGGCTAGGCAG | TCCGCGTAGCACTAATGTTCT |
| *hCYR61* | CTCGCCTTAGTCGTCACCC | CGCCGAAGTTGCATTCCAG |
| *hTGFB* | CTAATGGTGGAAACCCACAACG | TATCGCCAGGAATTGTTGCTG |
| *mActb* | TCCATCATGAAGTGTGACGT | GAGCAATGATCTTGATCTTCAT |
| *mIl6* | CCAATTTCCAATGCTCTCCT | ACCACAGTGAGGAATGTCCA |
| *mIl1a* | GCACCTTACACCTACCAGAGT | AAACTTCTGCCTGACGAGCTT |
| *mIl8* | TTGGTGATGCTGGTCATCTT | TTTAGATGCAGCCCAGACAG |


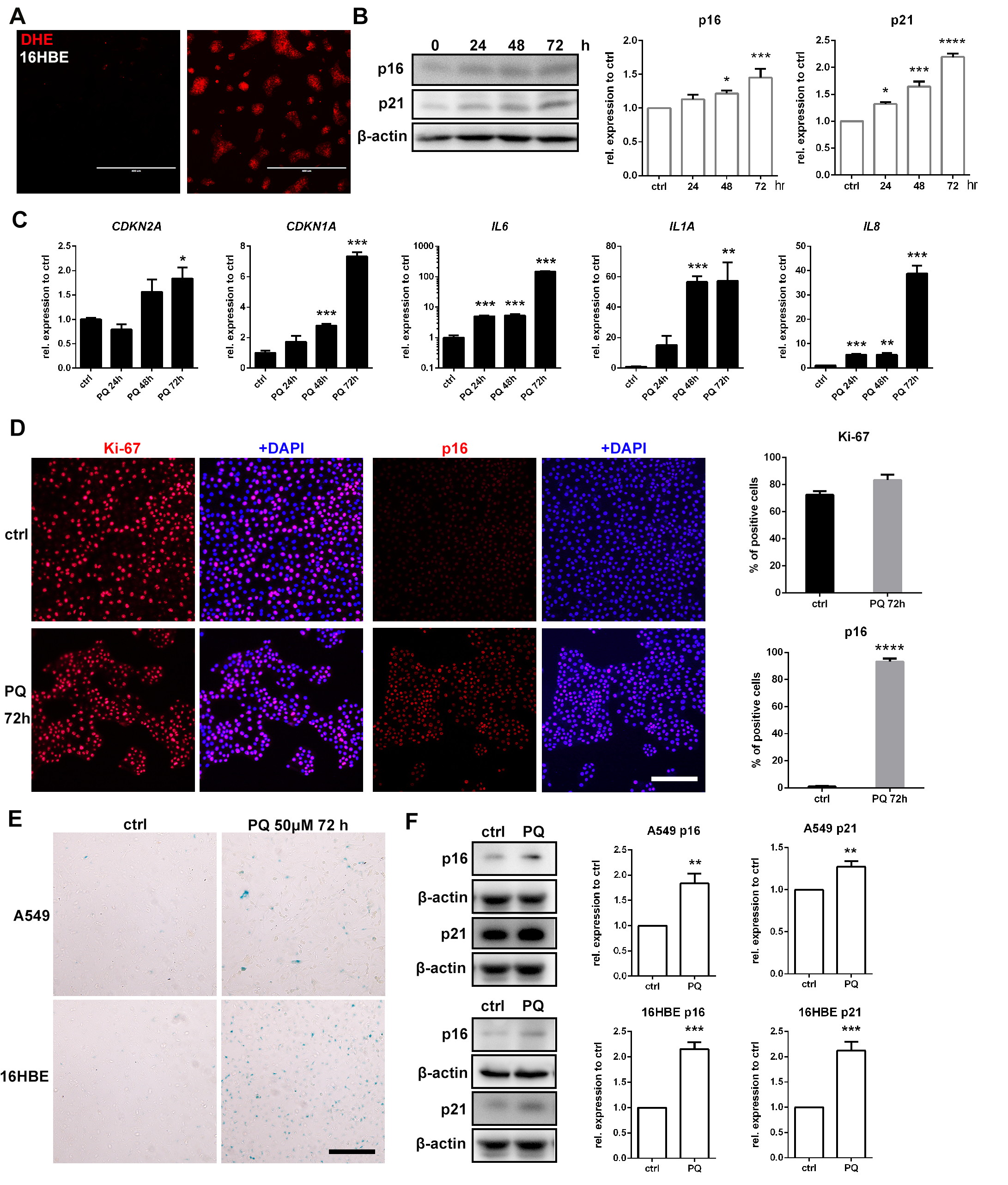


**Figure S1.** Paraquat induces 16HBE cell senescence: (A) ROS content in 16HBE cells after treated with 200 μM PQ for 72 h was analyzed with DHE staining (red) (original magnification 200×), (B) 16HBE cells treated with 200 μM PQ for 24 h, 48 h and 72 h were harvested and assessed for p16 and p21, with β-actin as loading control by Western blotting, (C) relative mRNA levels of CDKN2A (p16), CDKN1A (p21), and SASP markers IL6, IL1a and IL8 compared to ACTB in 16HBE cells treated with 200 μM PQ for 24 h, 48 h and 72 h were analyzed by qRT-PCR, (D) immunoflourescence staining of 16HBE cells treated with 200 μM PQ for 72 h for Ki-67 (red), p16 (red) and DAPI (blue) (original magnification 200×), and quantification of positive cells. (E) A549 and 16HBE cells were stained with SA**-**β-gal after treated with 50 μM PQ for 72 h (original magnification 200**×**). (F) A549 and 16HBE cells treated with 50 μM PQ for 72 h were harvested and assessed for p16 and p21, with β-actin as loading control by Western blotting. All statistical data were from three independent experiments. Values are shown as mean ± SEM. Data were analyzed by Student’s t test between 2 groups, and one-way ANOVA with the Dunnett's correction was used for comparisons among multiple groups. *P < 0.05, **P < 0.01, ***P < 0.005, ****P < 0.001.


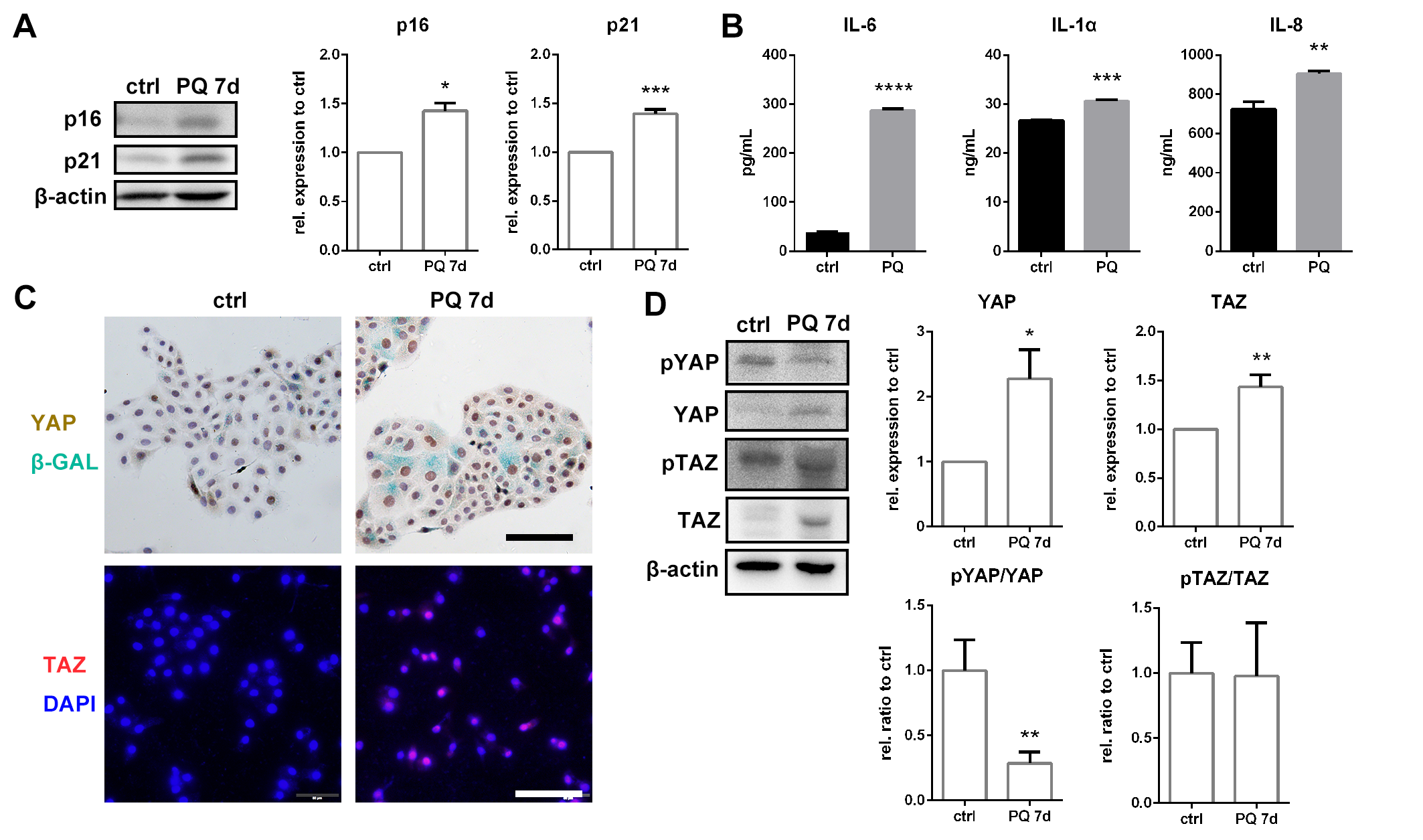


**Figure S2.** Establishment of senescent 16HBE cell model and YAP/TAZ activation in senescent 16HBE cells: (A) 16HBE cells harvested at the 7th day after exposed to 200 μM PQ for 24 h were assessed for p16 and p21, with β-actin as loading control by Western blotting, (B) SASP markers IL-6, IL-1α and IL-8 in the supernatant were tested by ELISA assay, (C) co-staining of SA-β-gal and YAP, and immunoflourescence staining for TAZ (red) and DAPI (blue) in senescent 16HBE cells (original magnification 400×), (D) senescent 16HBE cells were assessed for pYAP, YAP, pTAZ, and TAZ, with β-actin as loading control by Western blotting. All graphs are shown as mean±SEM. Parametric variables were calculated using two-tailed Student’s t test between 2 groups. *P < 0.05, **P < 0.01, ***P < 0.005, ****P < 0.001.


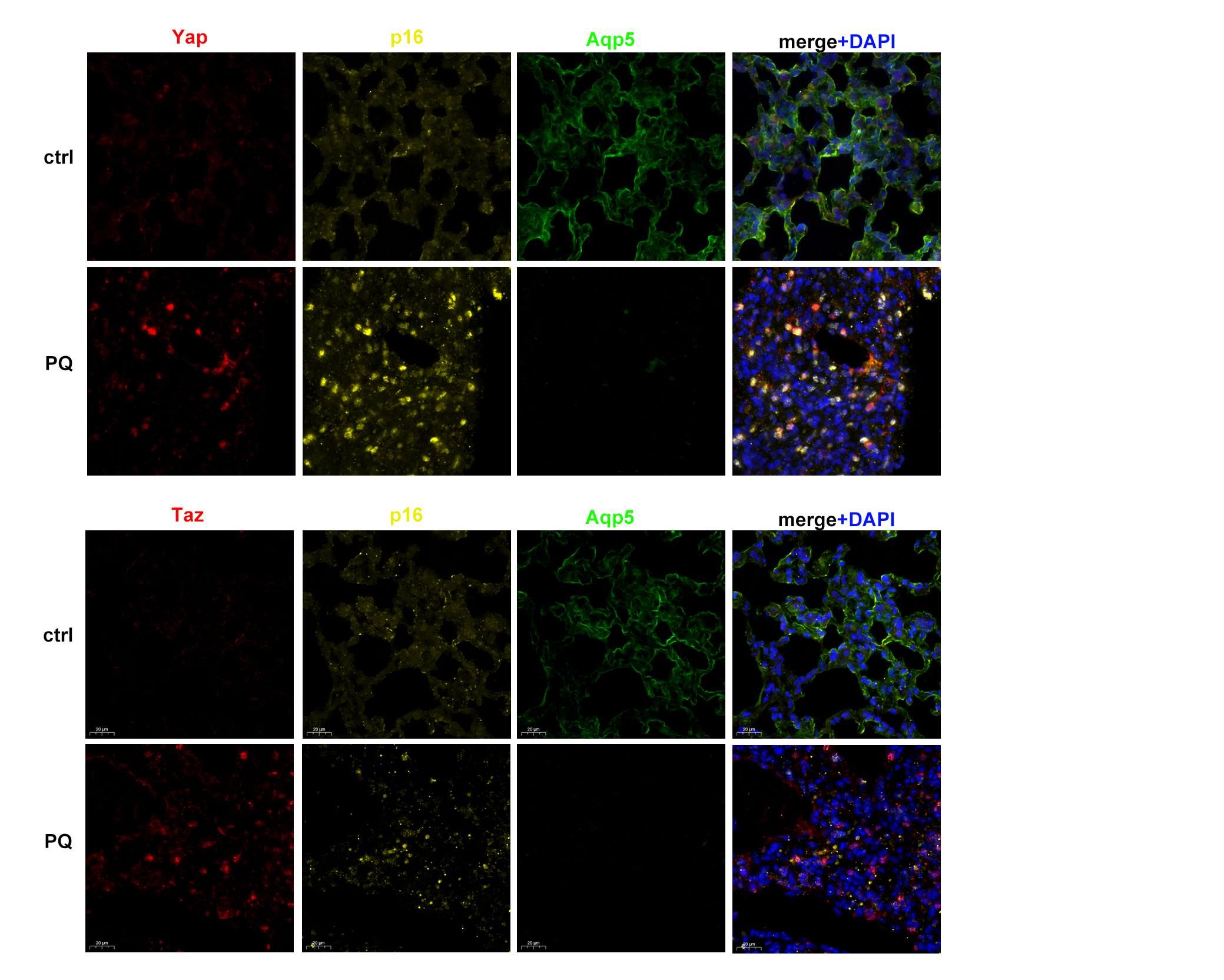


**Figure S3.** Representative images of immunohistochemistry staining of YAP/TAZ (red), p16 (yellow), Aqp5 (green) and DAPI (blue) in PQ treated mice and control mice lung tissues (original magnification 400×).


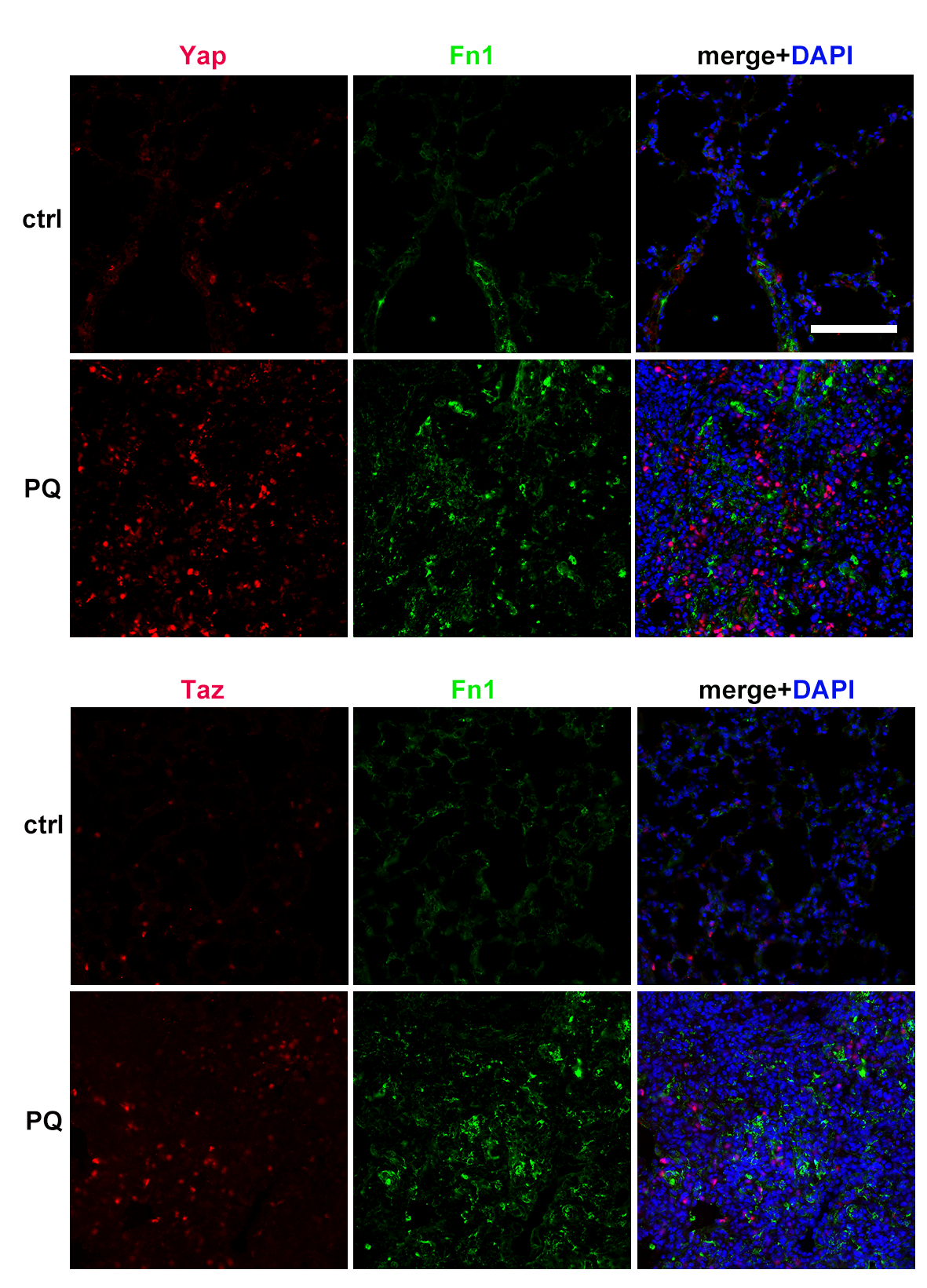


**Figure S4.** Representative images of immunohistochemistry staining of YAP/TAZ (red), Fn1 (green) and DAPI (blue) in PQ treated mice and control mice lung tissues (original magnification 400×).


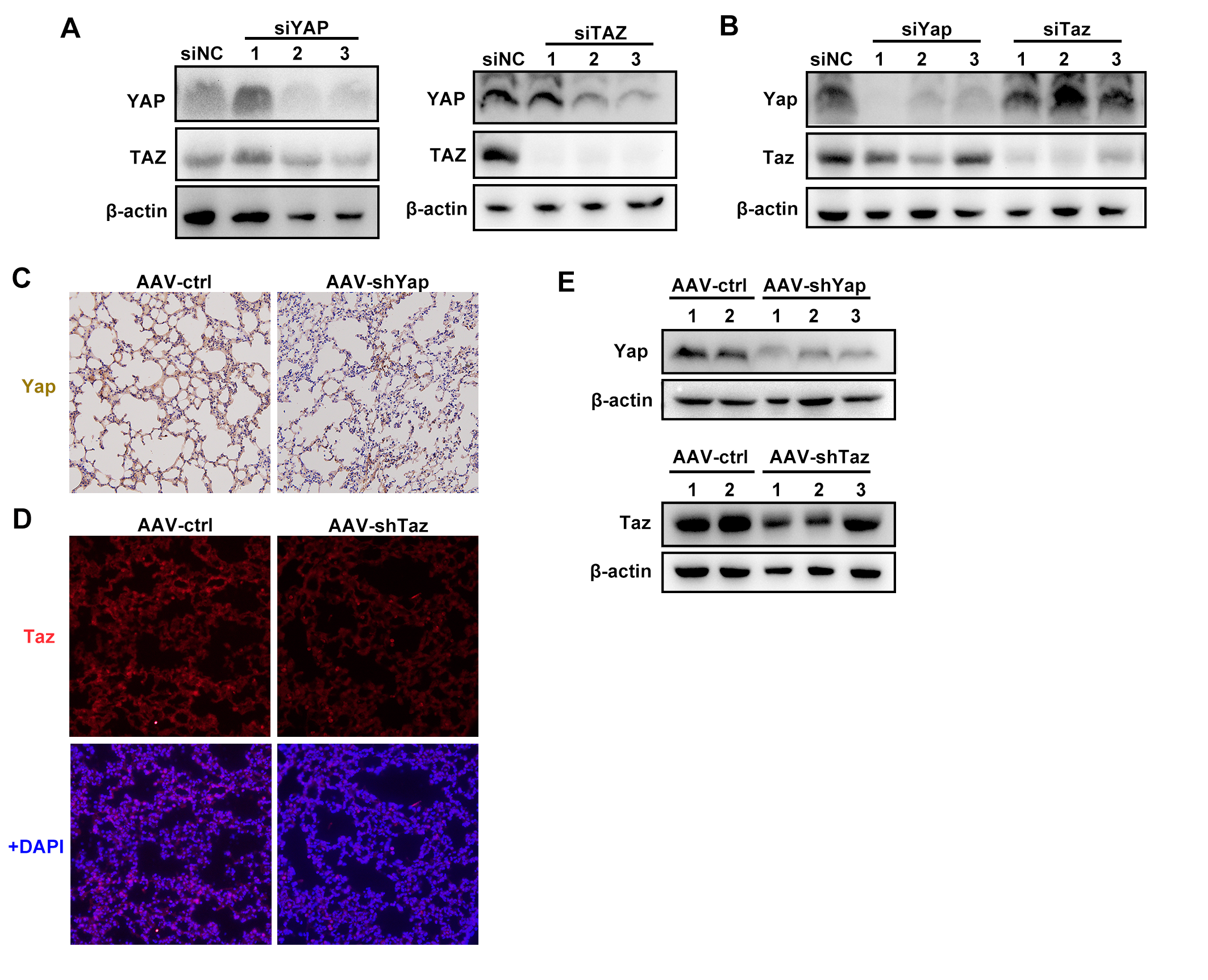


**Figure S5.** RNA-interference efficiency *in vitro* and *in vivo*: (A) 16HBE cells were transfected with 3 siRNAs targeting human YAP or TAZ separately, harvested after 48 h, and analyzed for YAP and TAZ, with β-actin as loading control by Western blotting, (B) L929 cells were transfected with 3 siRNAs targeting mouse Yap or Taz separately, harvested after 48 h, and analyzed for Yap and Taz, with β-actin as loading control by Western blotting. (C) immunohistochemistry staining for YAP in mouse lung tissues after intratracheal infected with 5 × 10^10^ PFU of AAV-Ctrl or AAV-shYap for 3 weeks (original magnification 100×), (D) immunoflourescence staining for TAZ (red) and DAPI (blue) in mouse lung tissues after intratracheal infected with 5 × 10^10^ PFU of AAV-Ctrl or AAV-shTaz for 3 weeks (original magnification 100×). (E) lung tissues were lysed and analyzed for Yap and Taz, with β-actin as loading control by Western blotting.


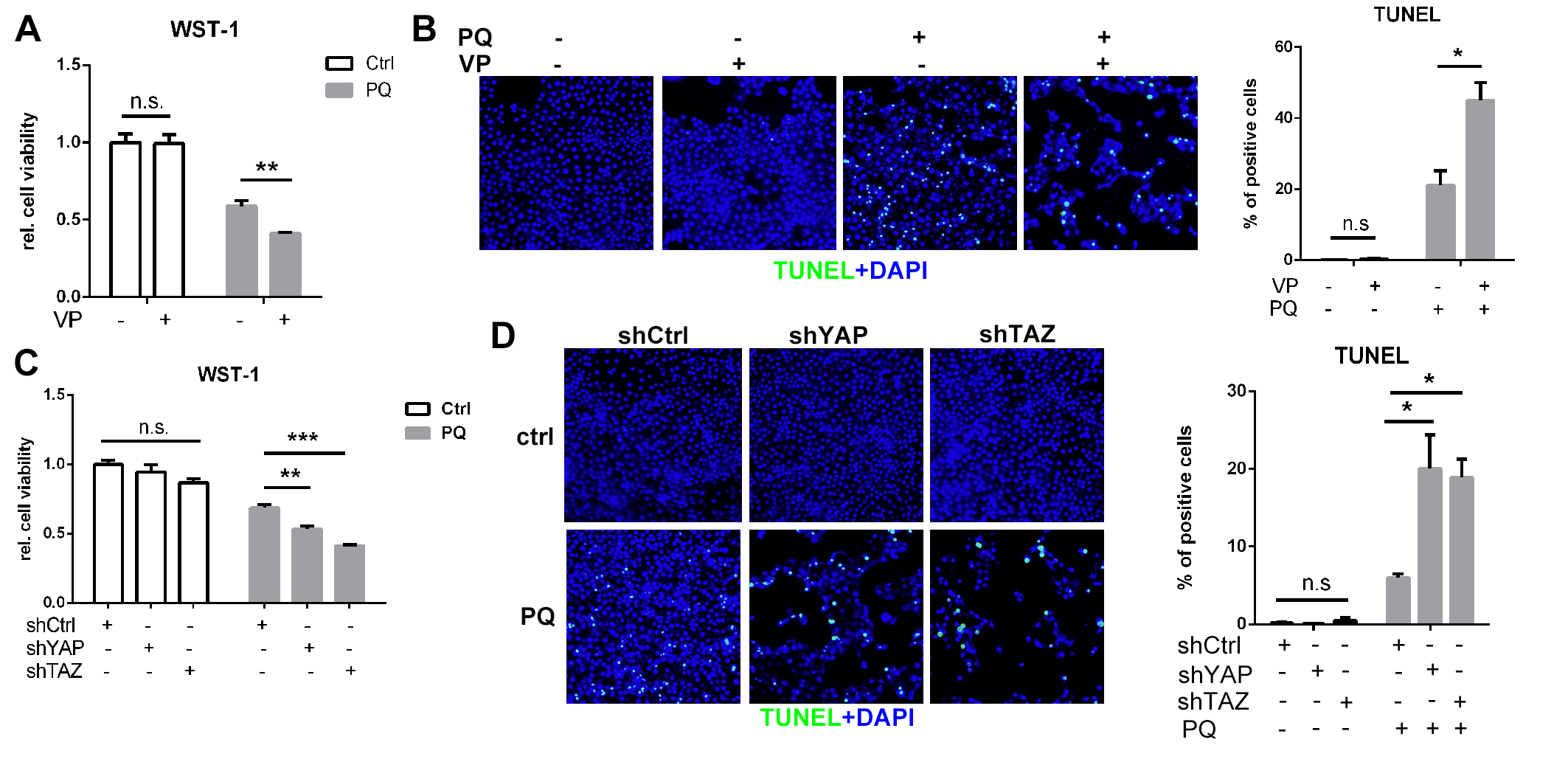


**Figure S6.** YAP and TAZ activation protects paraquat induced senescent cells from apoptosis: 16HBE cells were treated with 2 μM verteporfin (VP) for 24 h and than treated with 200 μM PQ for 24 h, and detected after another 24 h. Cell viability was assessed by WST-1 analysis (A), apoptosis was detected by TUNEL staining, and positive cells were quantified (B). 16HBE cells were infected with lentivirus interfering YAP (shYAP), TAZ (shTAZ) or control lentivirus seperately for 24 h and than treated with 200 μM PQ for 24 h, and detected after another 24 h. Cell viability was assessed by WST-1 analysis (C), apoptosis was detected by TUNEL staining, and positive cells were quantified (D). Values are shown as mean ± SEM. Data were analyzed by Student’s t test between 2 groups. *P < 0.05, **P < 0.01, ***P < 0.005, n.s.: no significance.
