# Supplementary material for: YAP/TAZ activation mediates PQ-induced lung fibrosis by sustaining senescent pulmonary epithelial cells
Source: Respir Res. 2024 May 18;25:212. doi: 10.1186/s12931-024-02832-z (PMC11102259; doi:10.1186/s12931-024-02832-z)

Fig1D

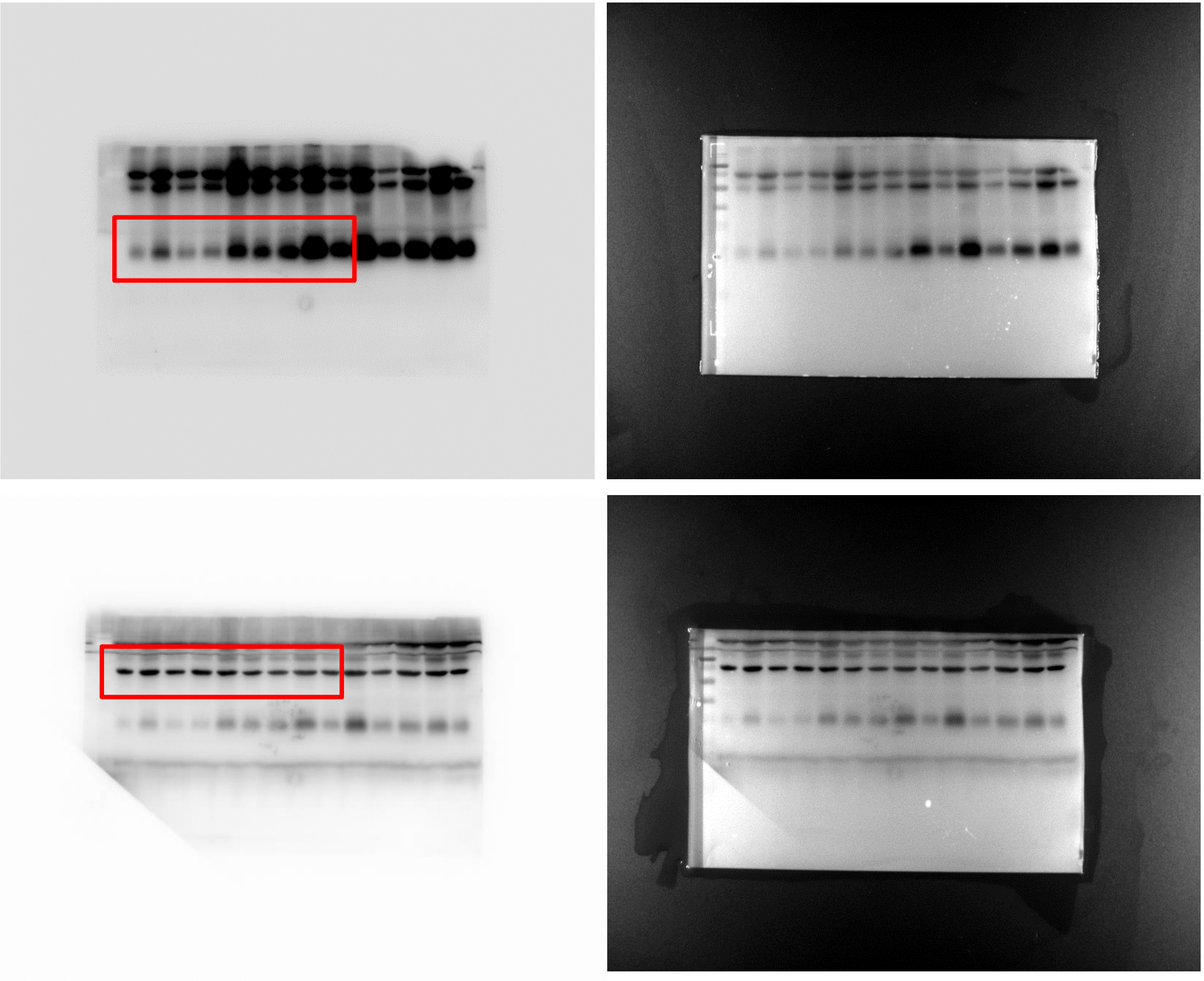

p16

P16-
β-actin

Fig1D

p21

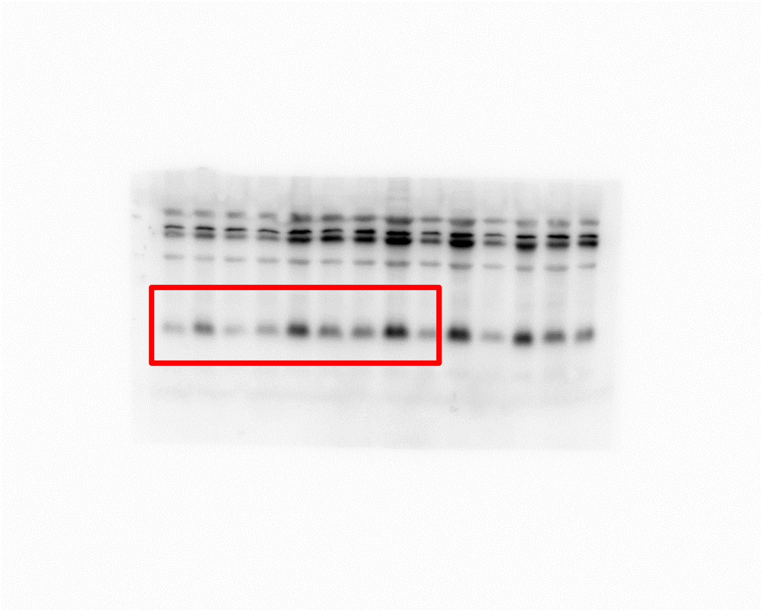

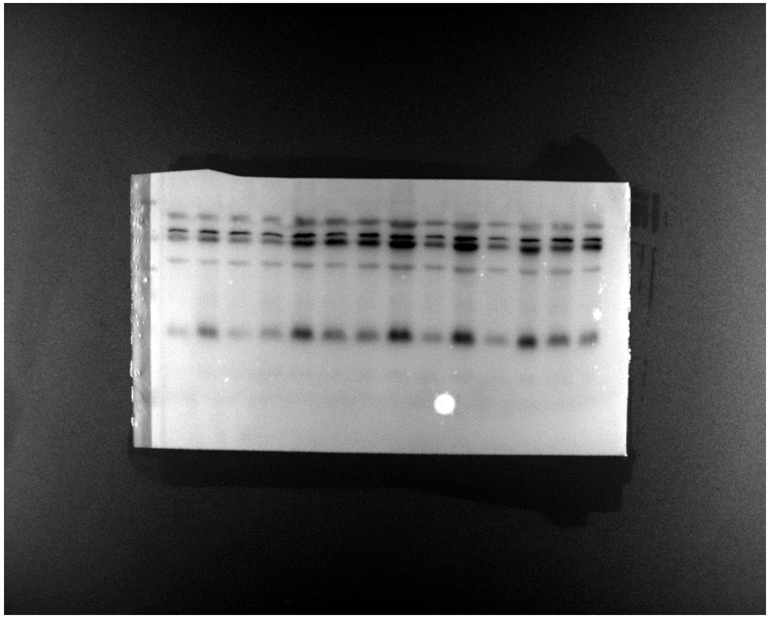

P21-
β-actin

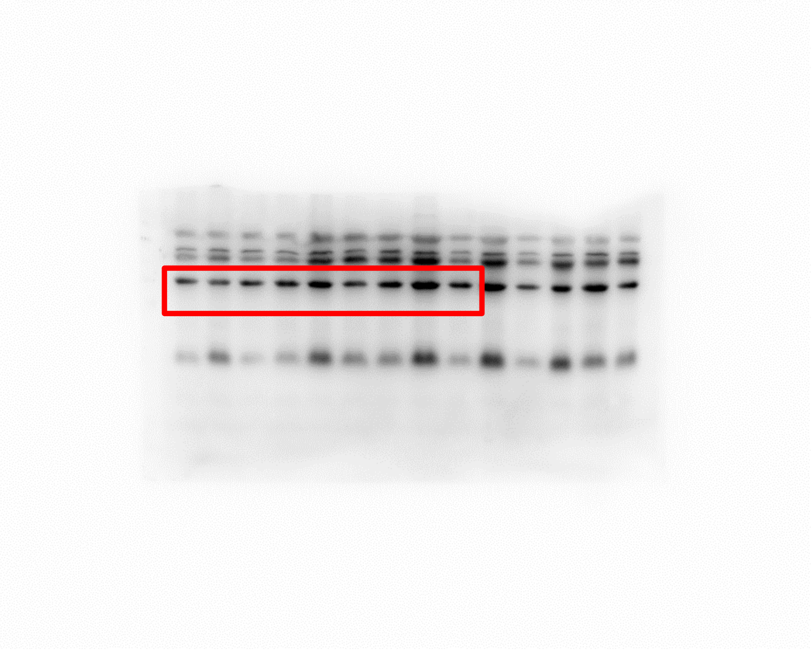

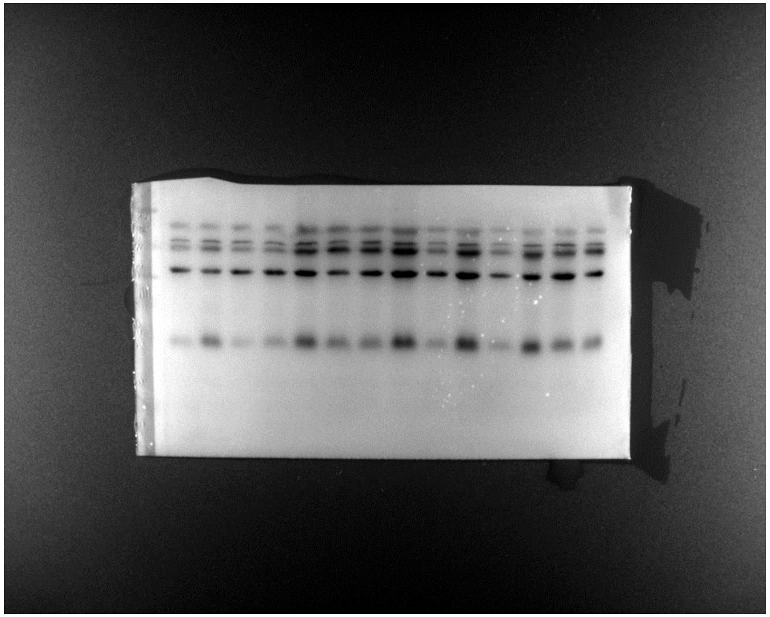

Fig2D

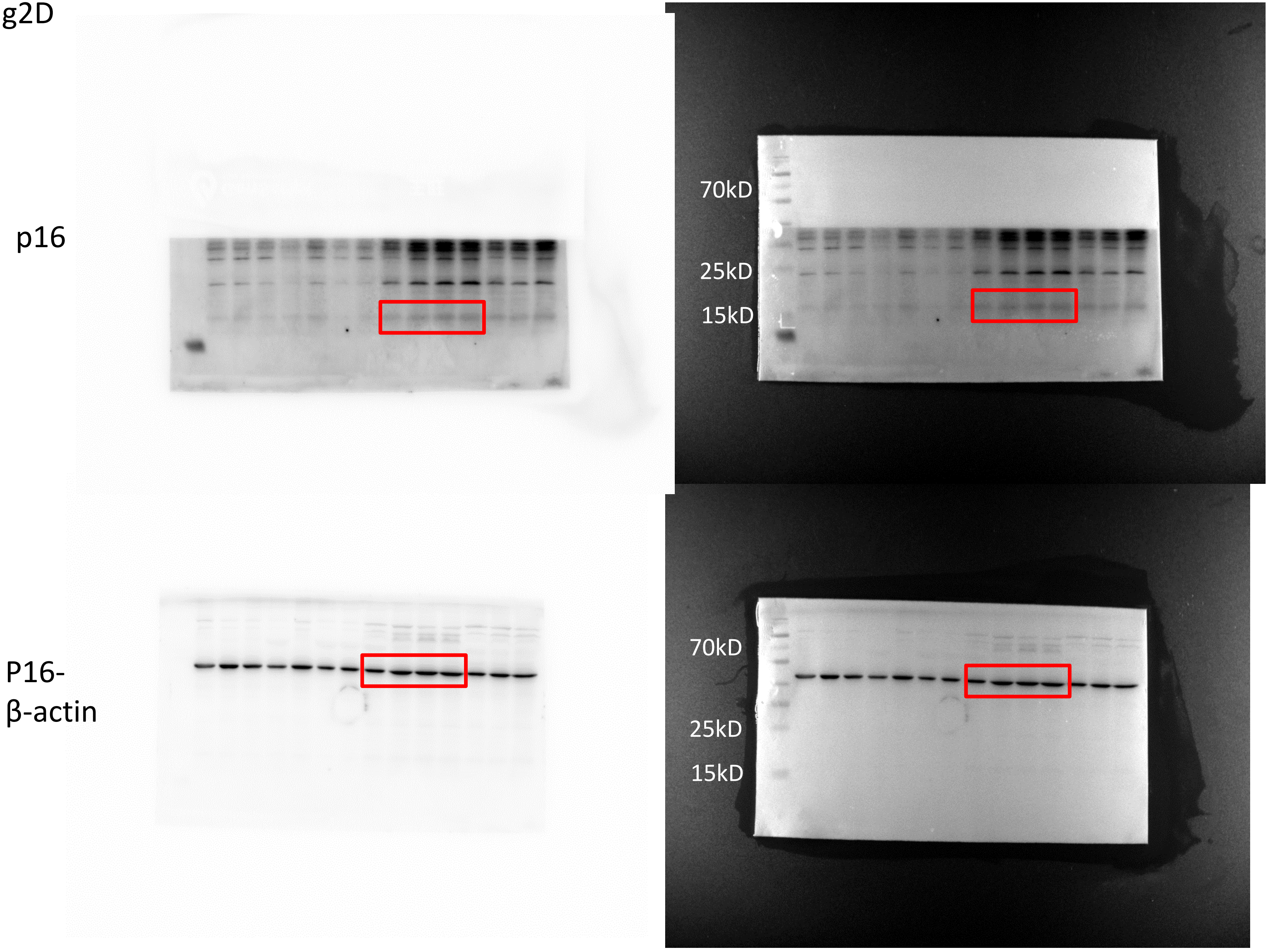

Fig2D

p21

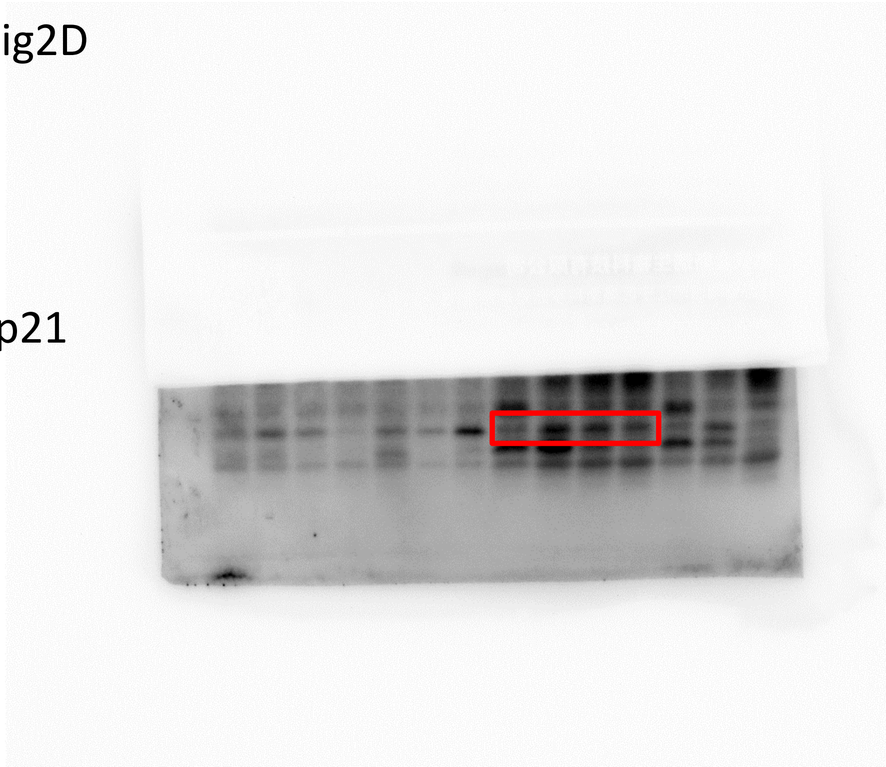

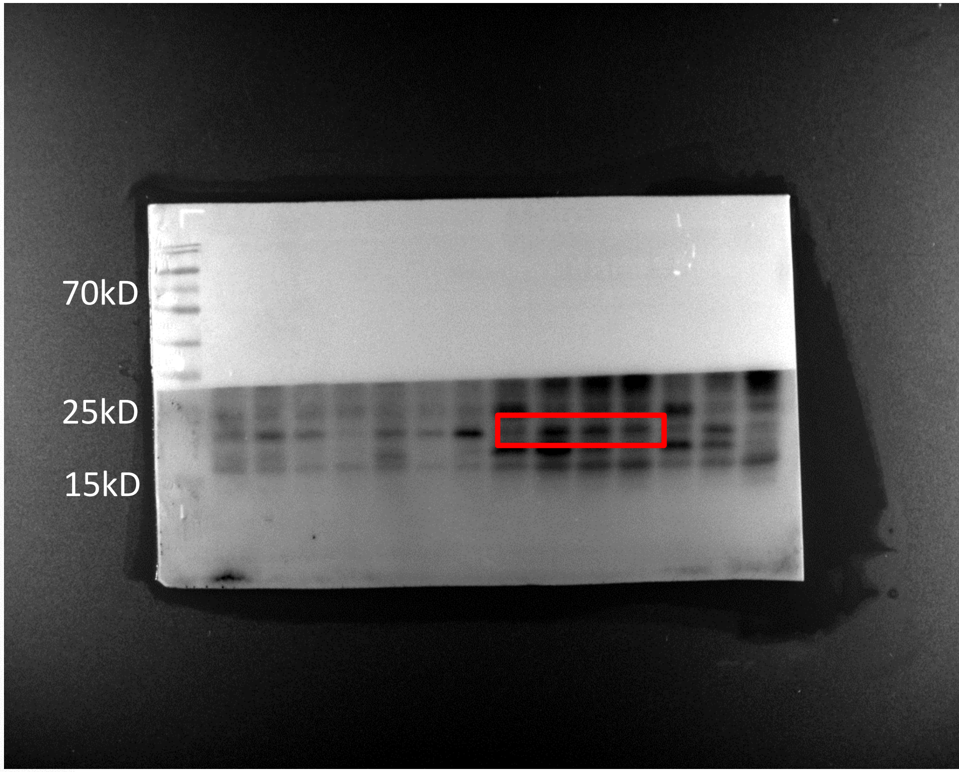

P21-
β-actin

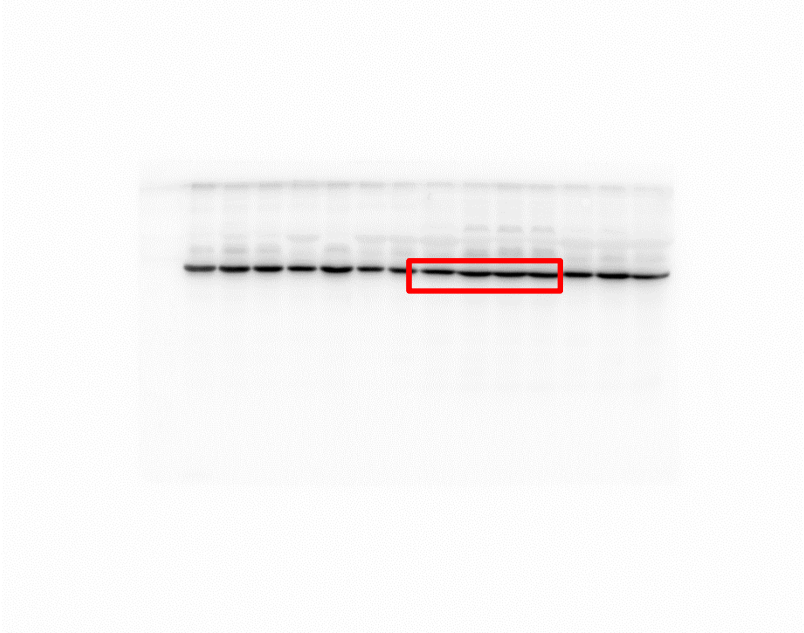

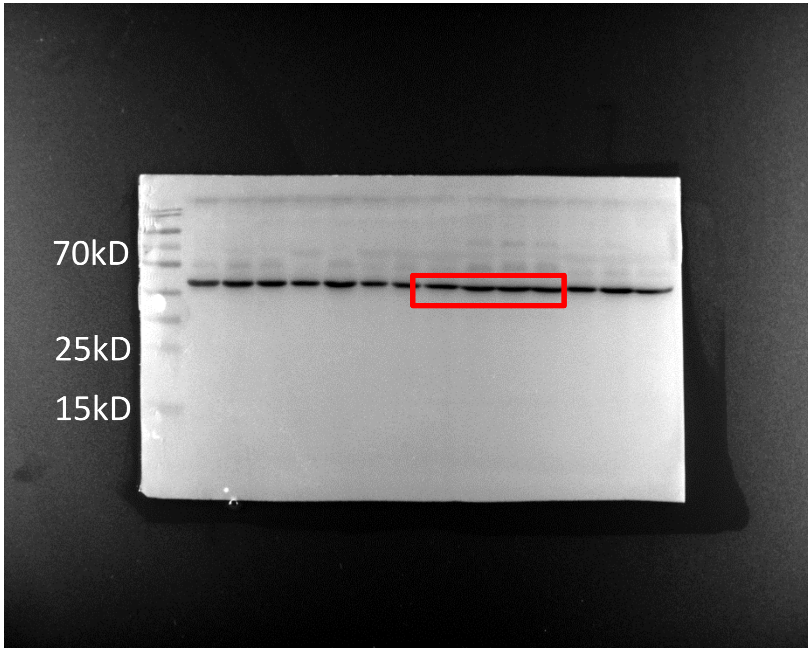

Fig3C

p16

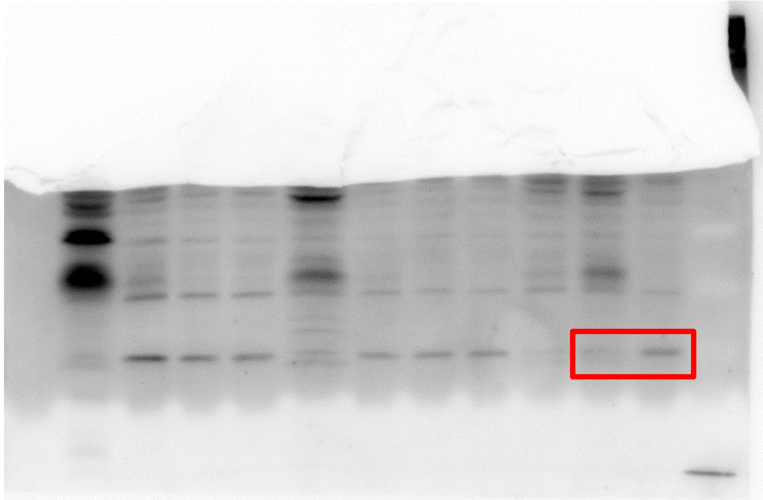

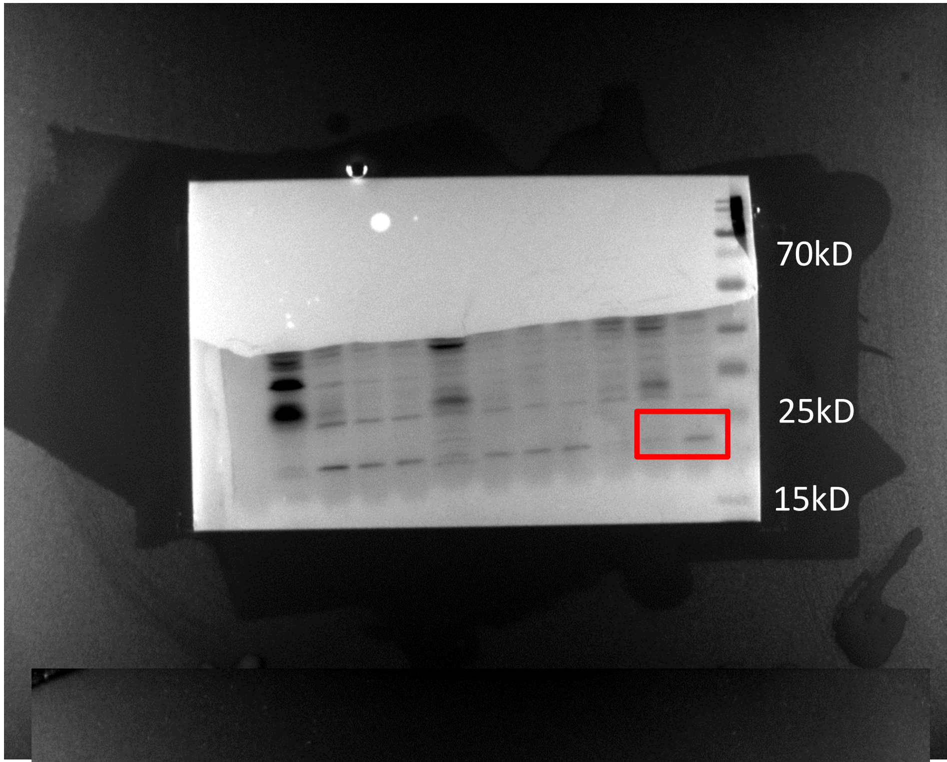

P16-
β-actin

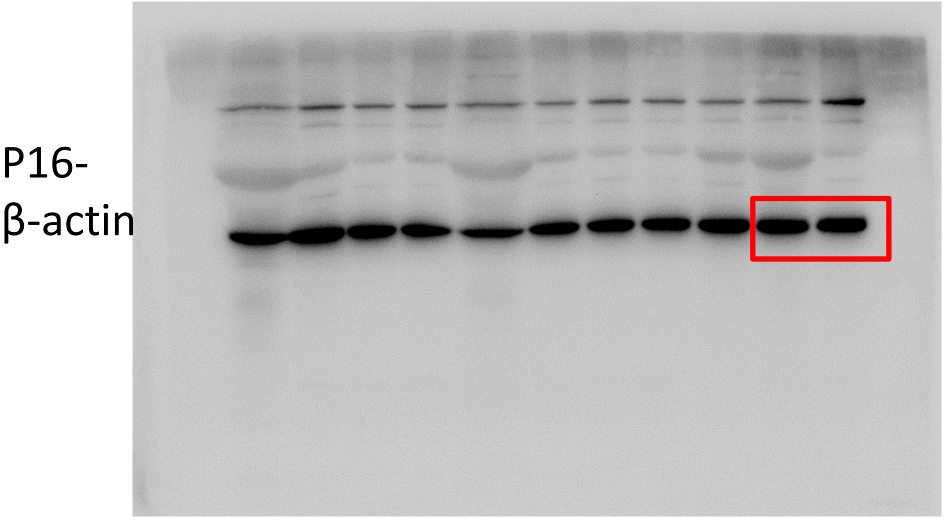

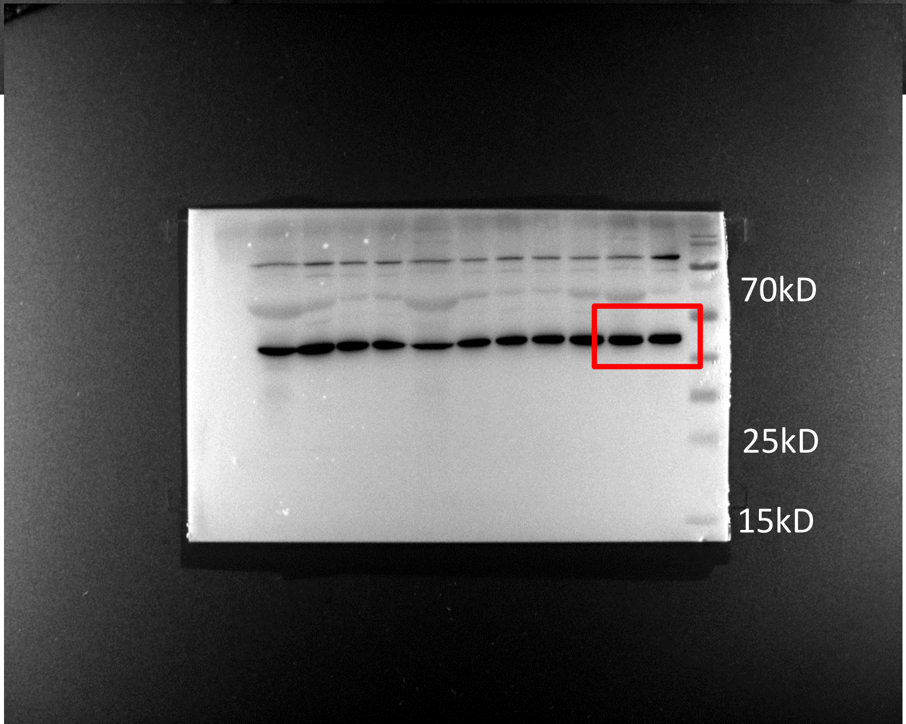

Fig3C

p21

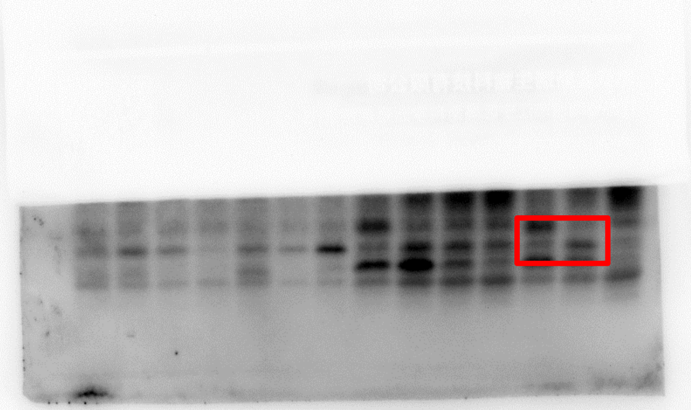

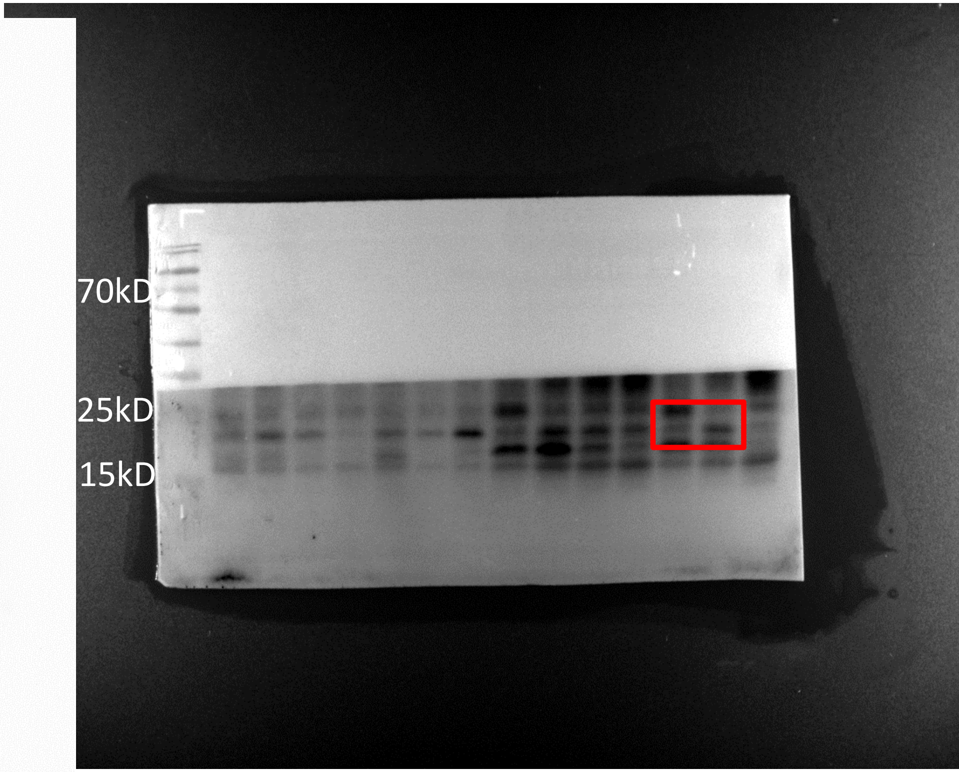

P21-
β-actin

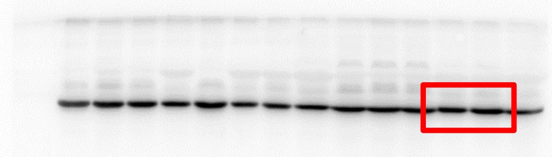

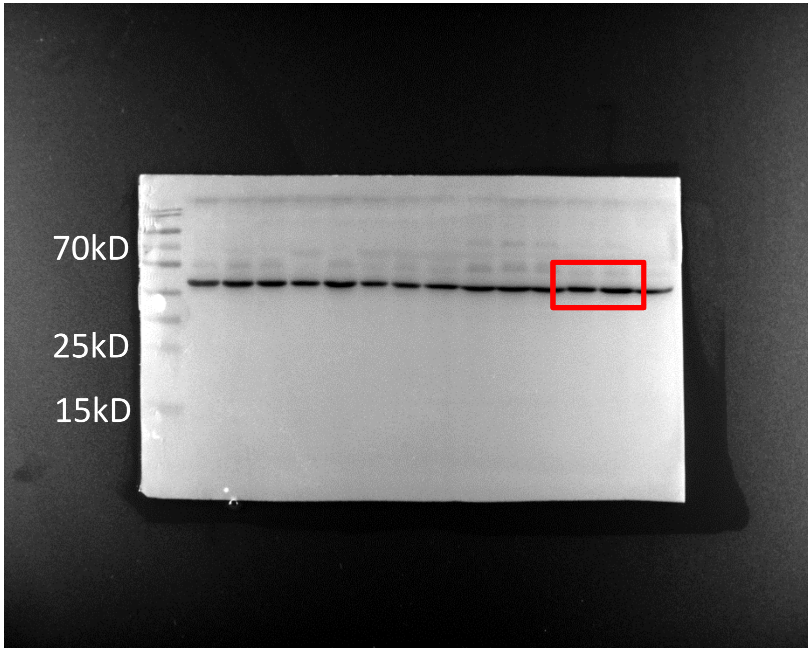

Fig3F

α-SMA

tubulin

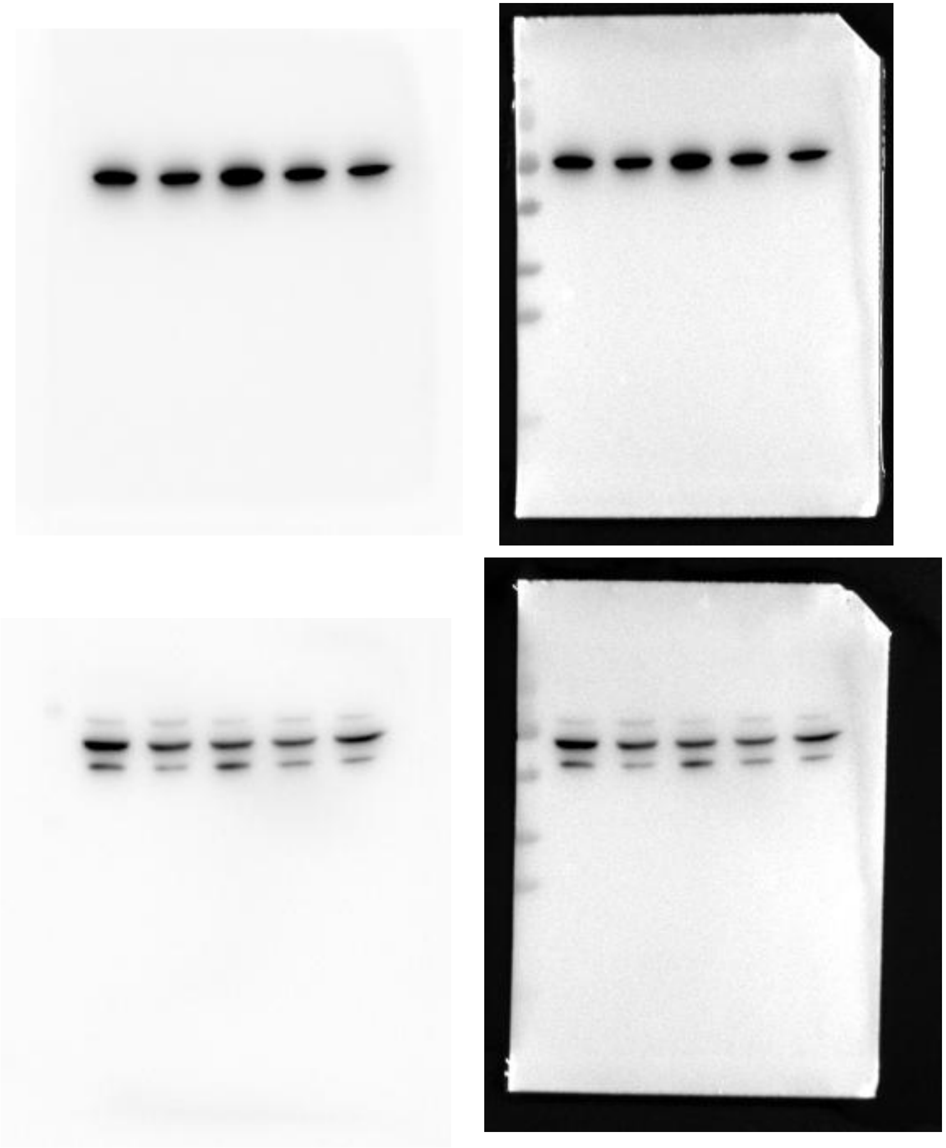

Fig4C

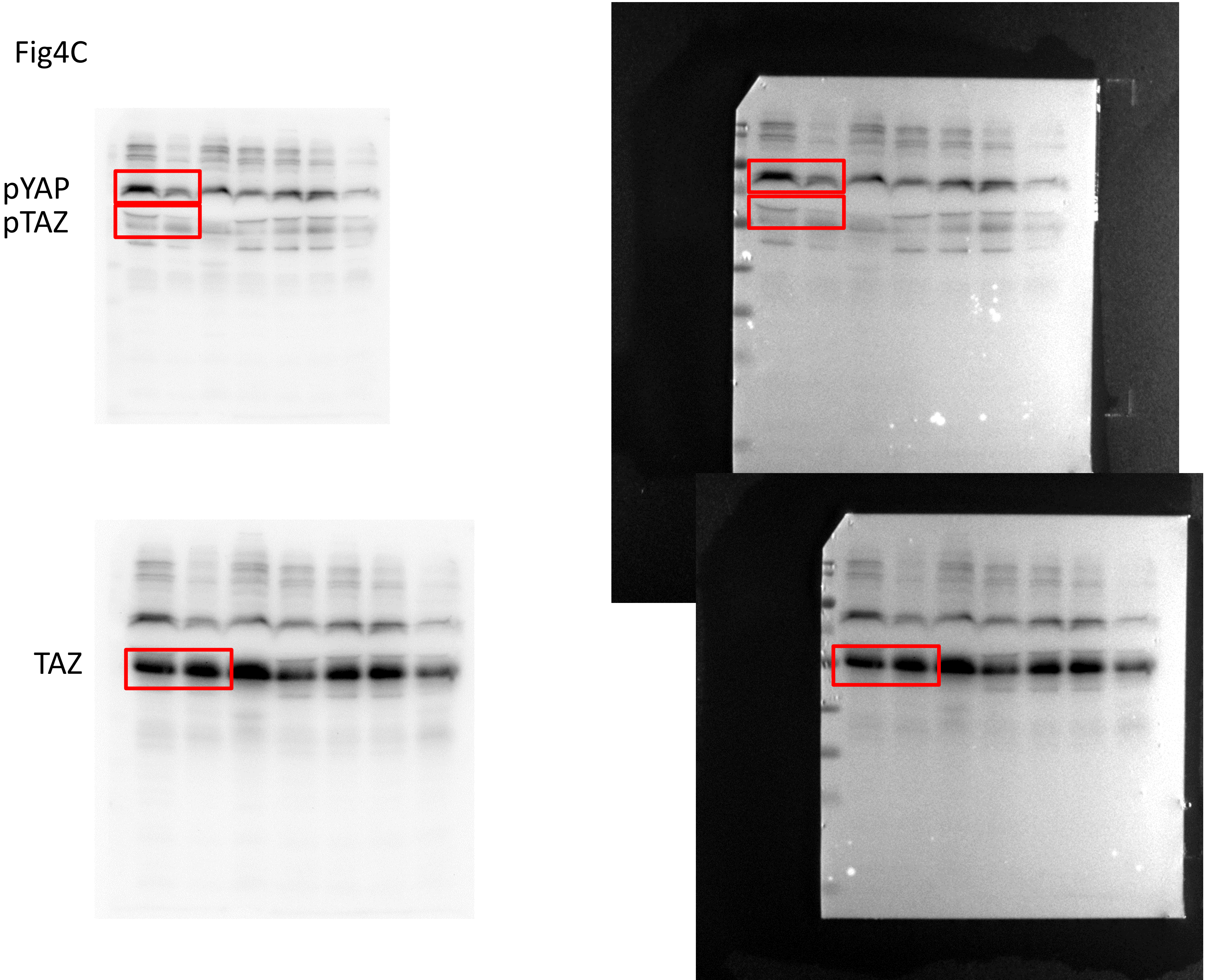

Fig4C

TAZ
β-actin

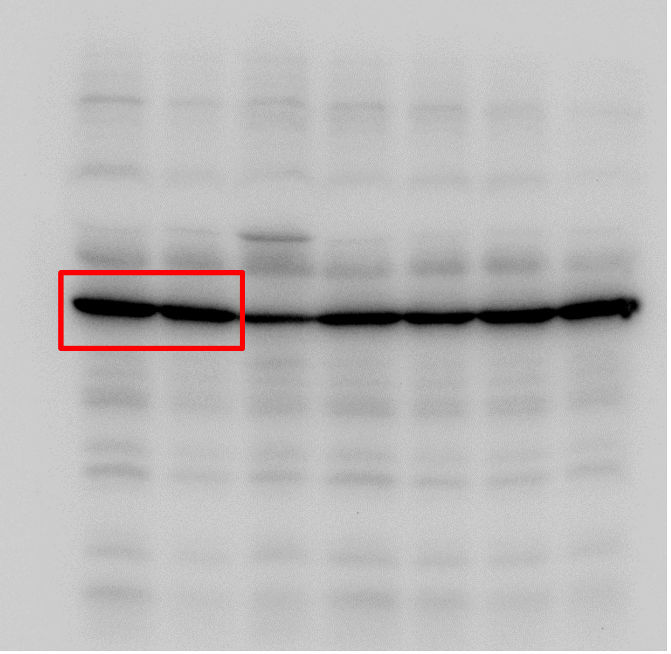

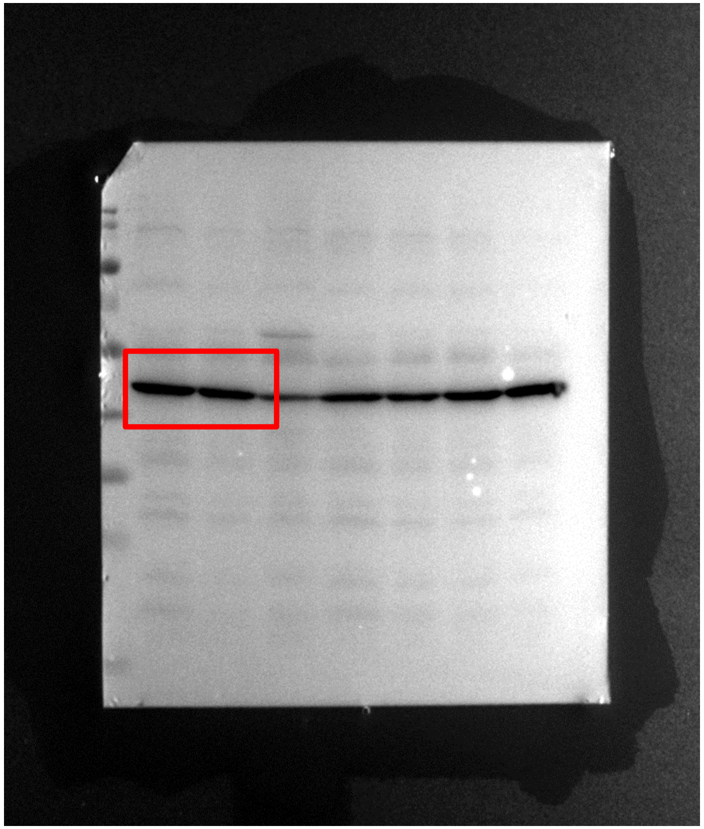

Fig4C

YAP

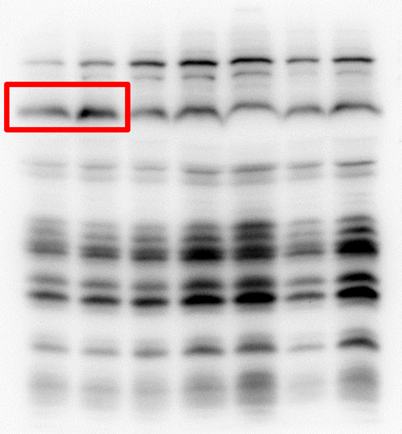

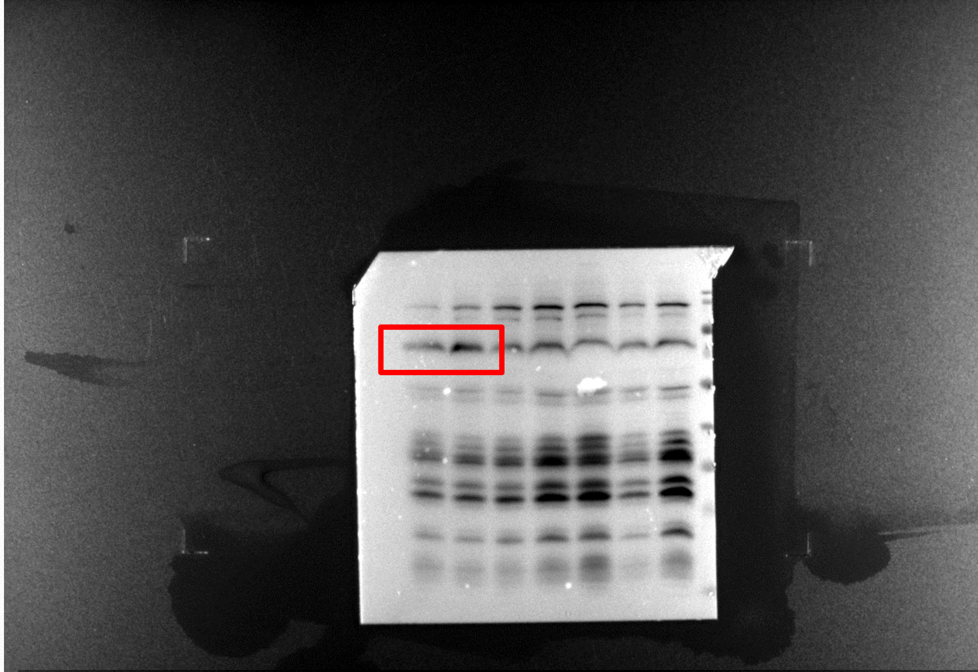

YAP
β-actin

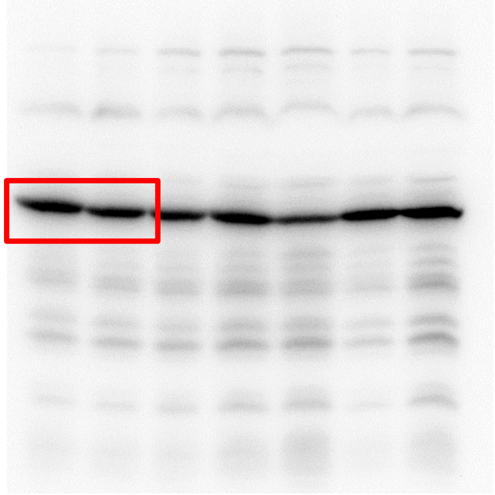

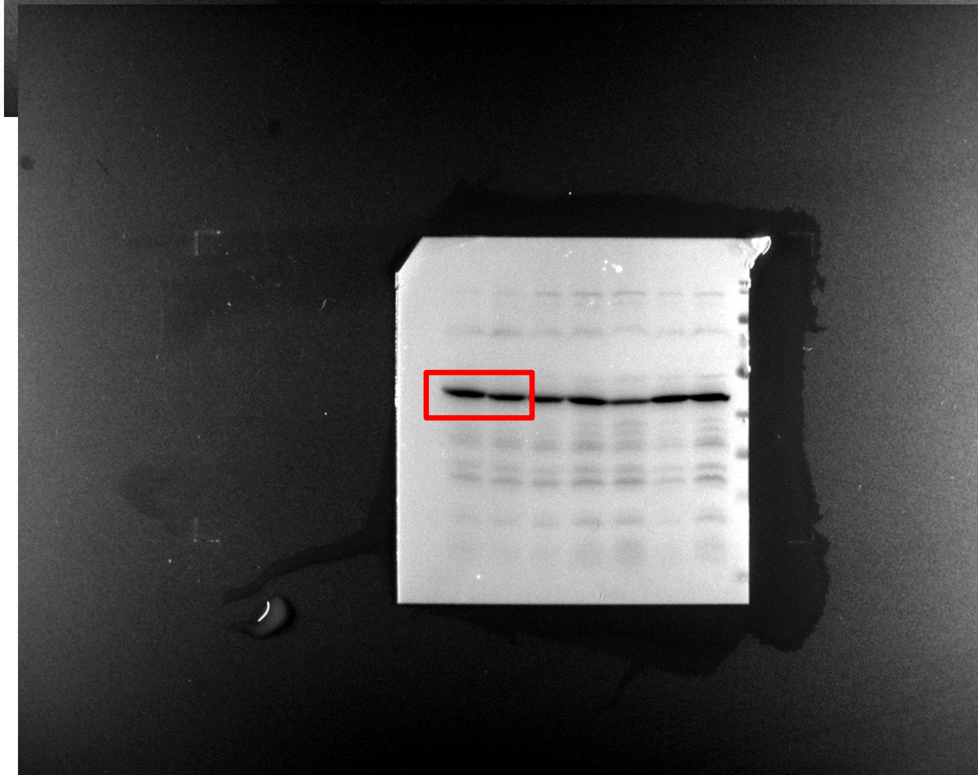

Fig4E

pYAP
pTAZ

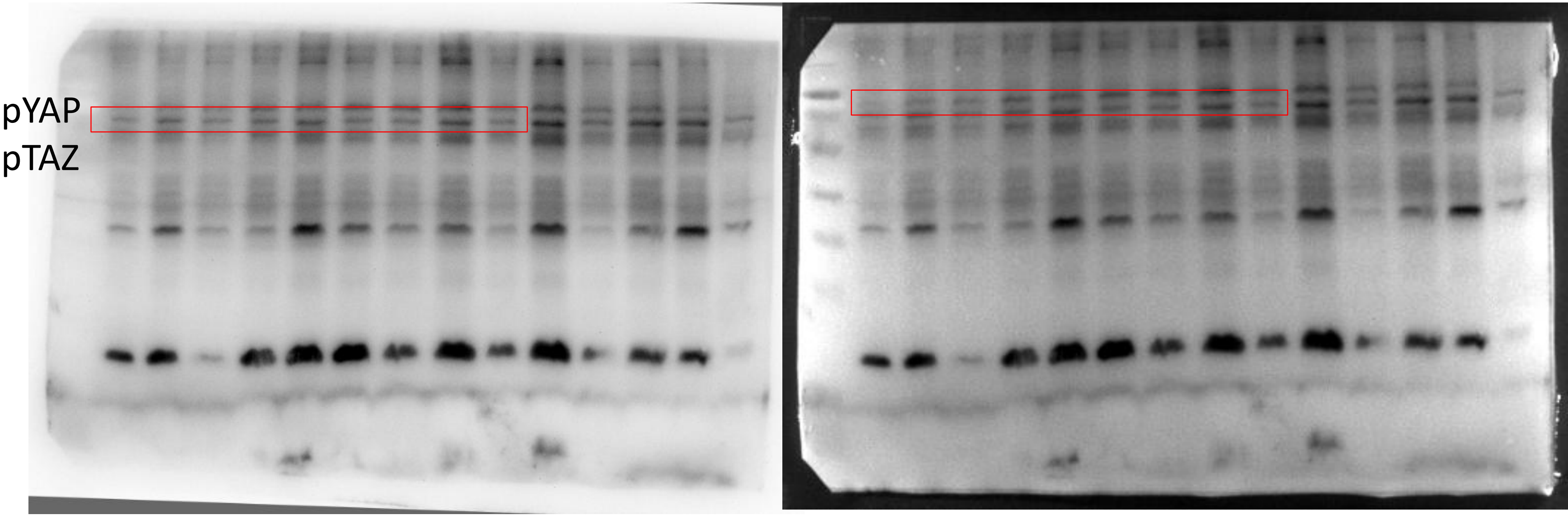

YAP

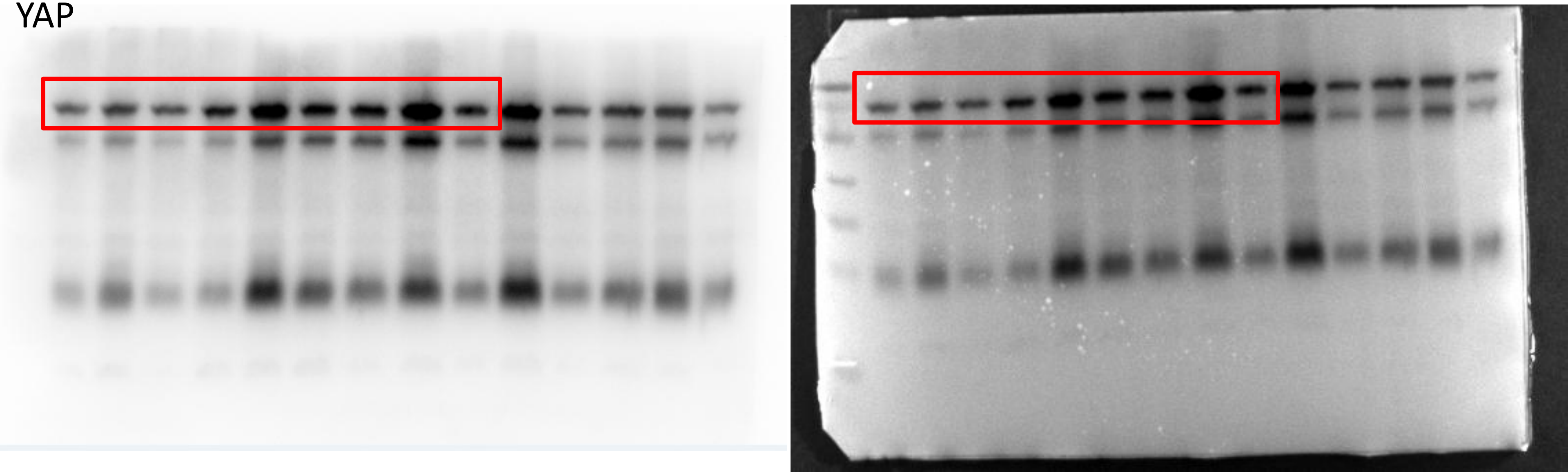

YAP
β-actin

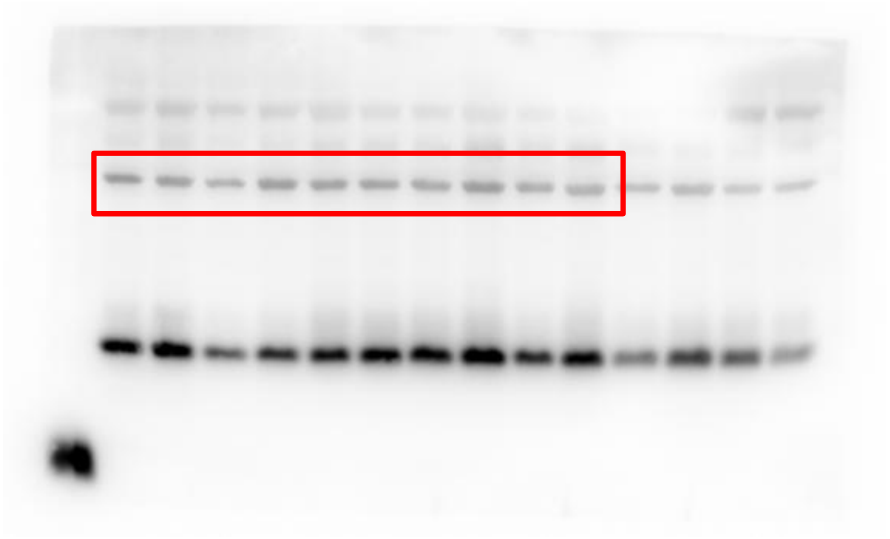

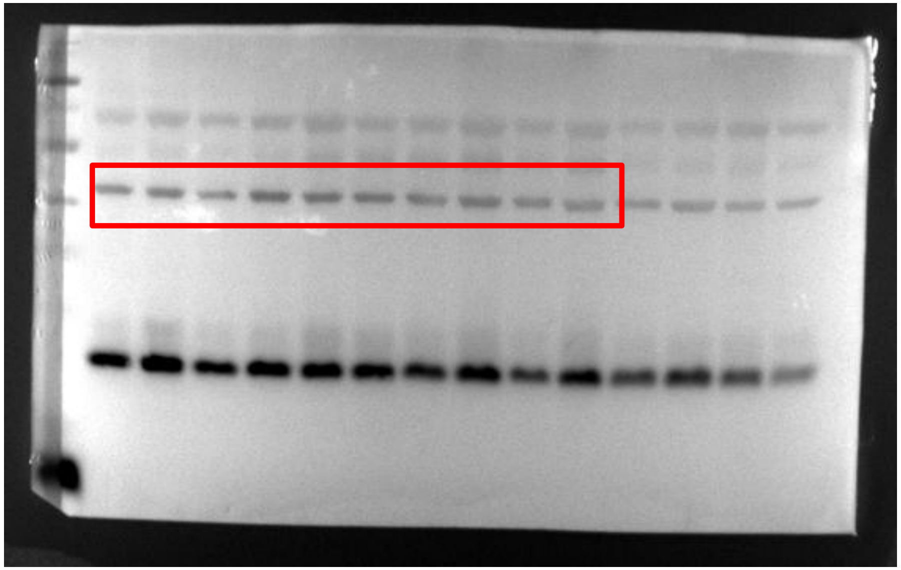

pTAZ

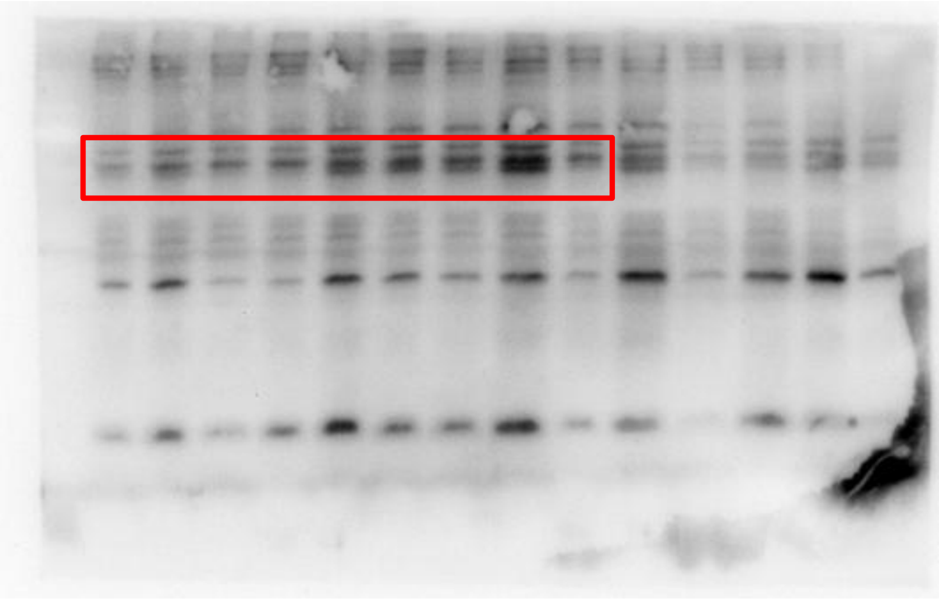

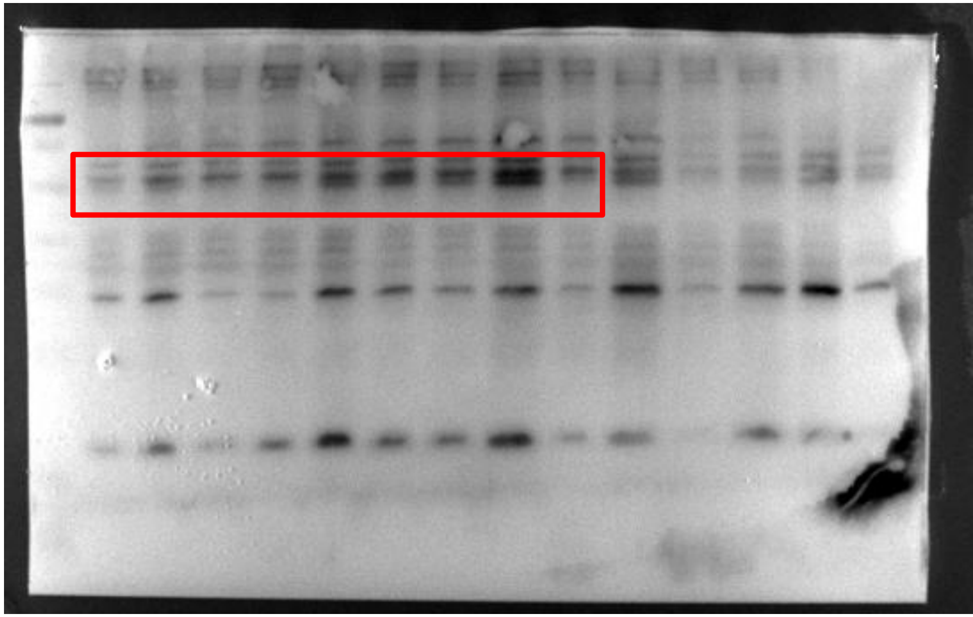

TAZ

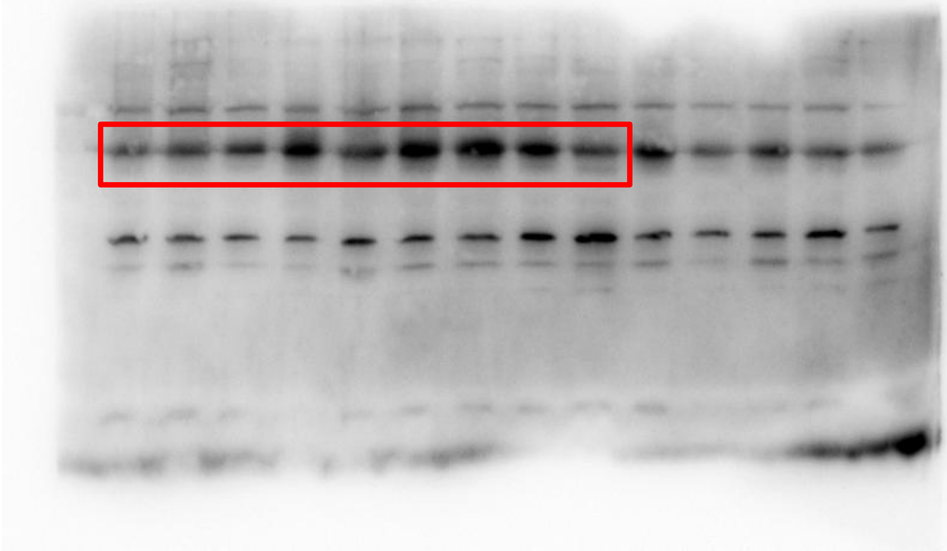

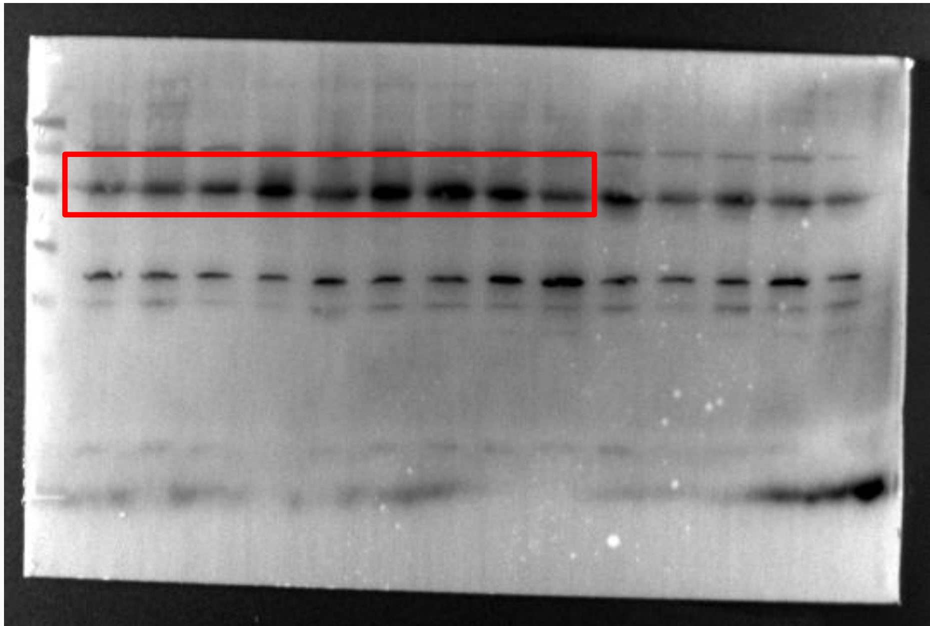

TAZ
β-actin

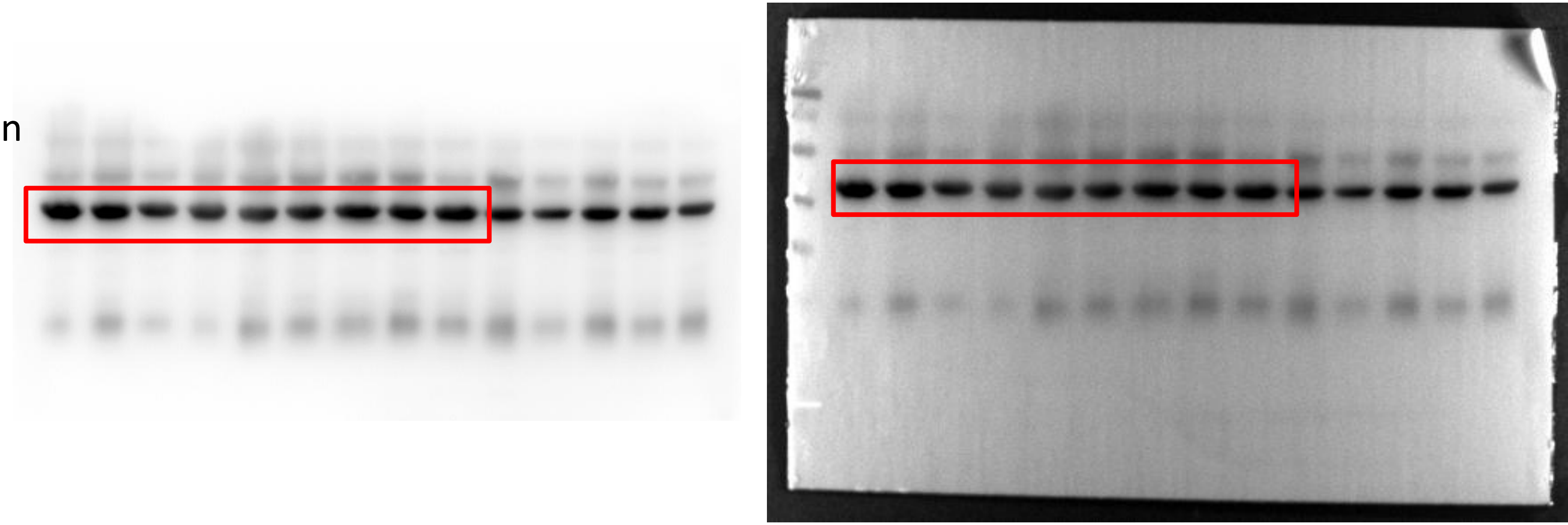

Fig6

Bax

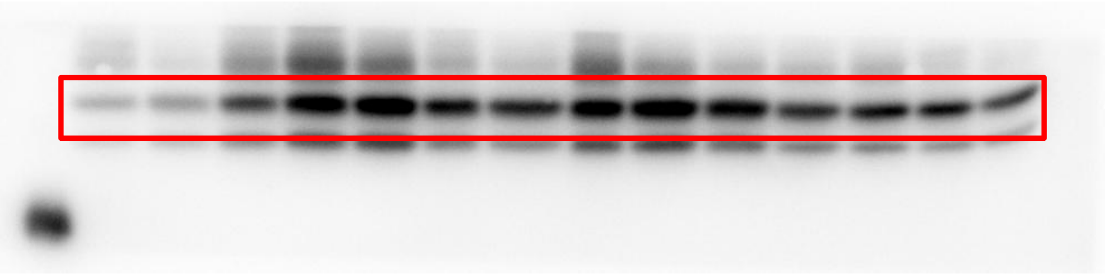

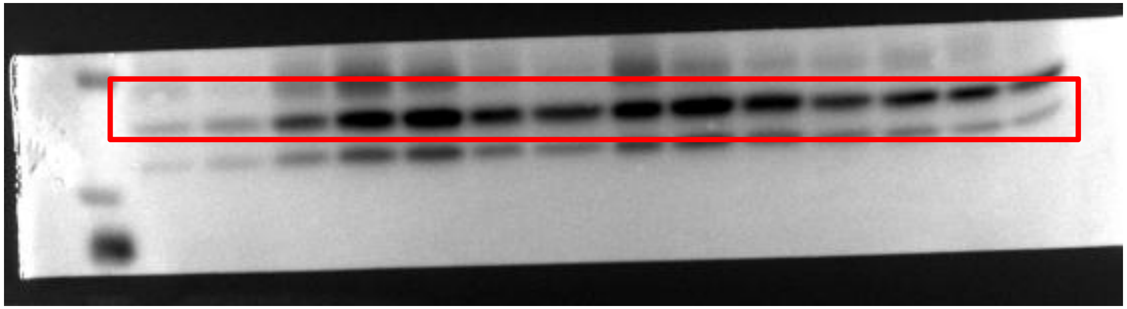

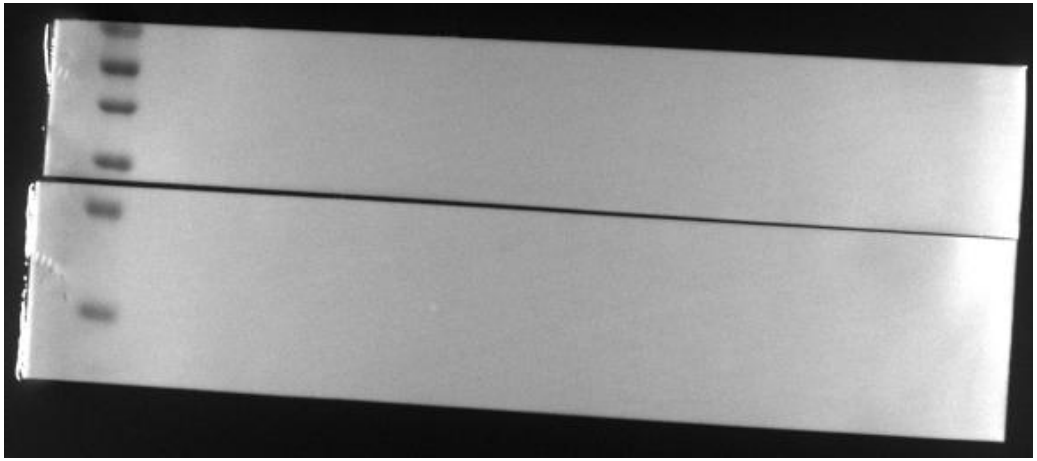

Fig6

Ctgf

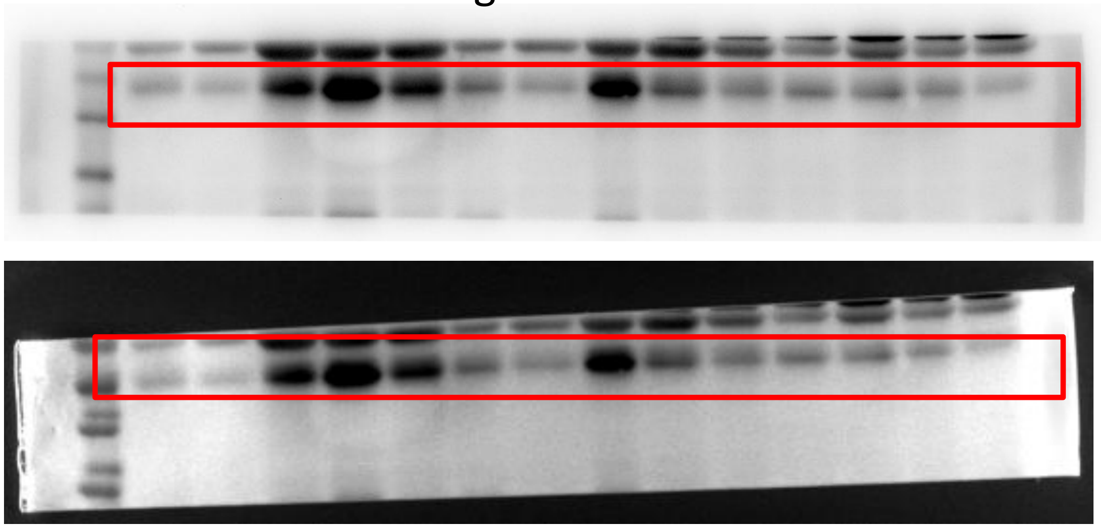

Bax+Ctgf

β-actin

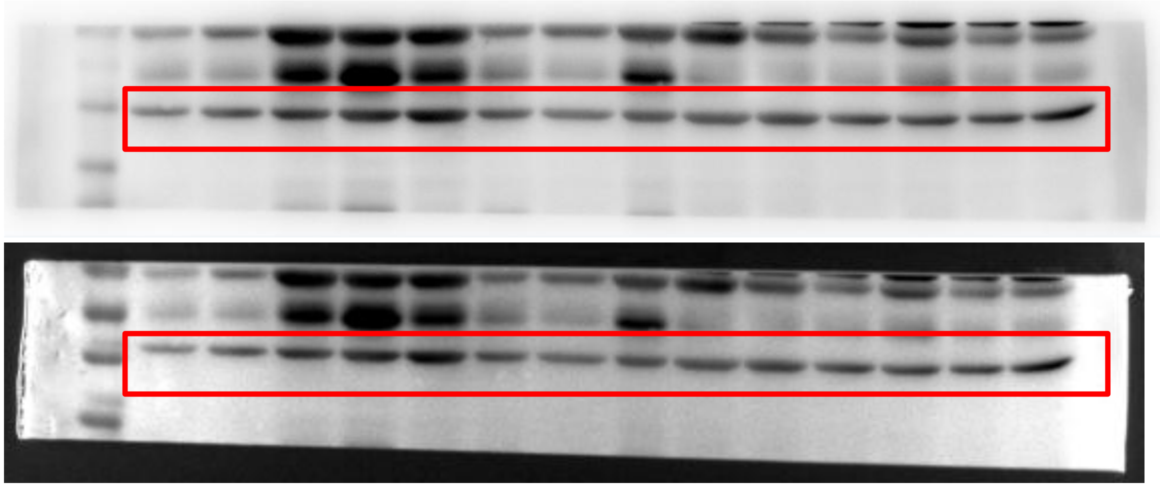

Fig6

p16

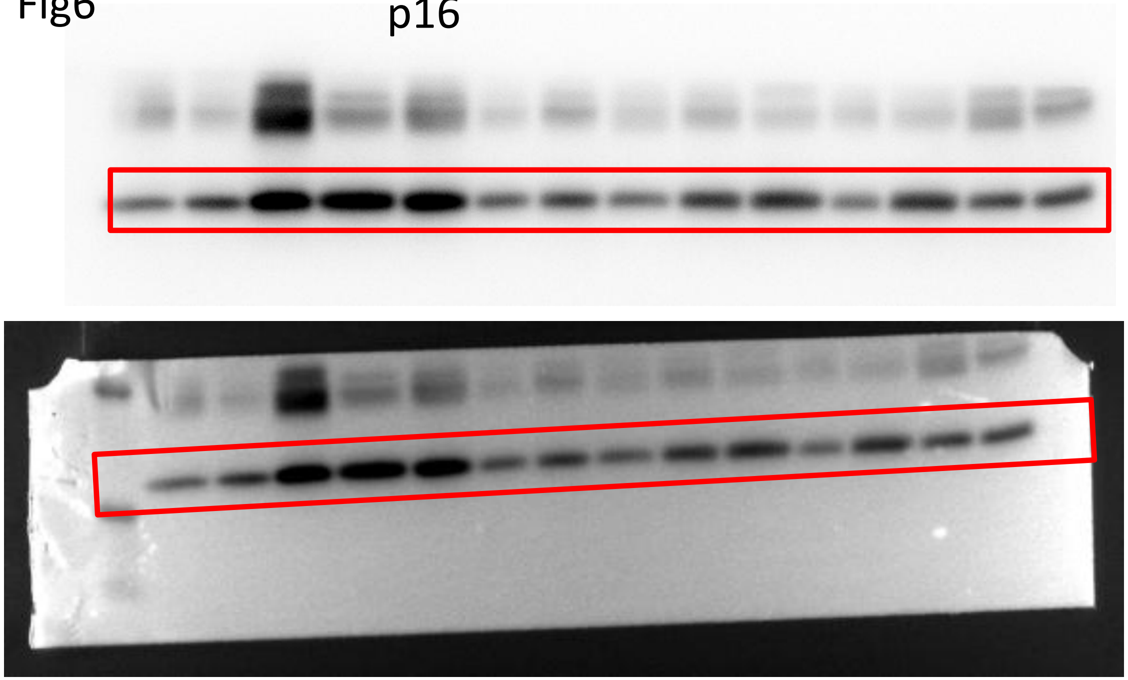

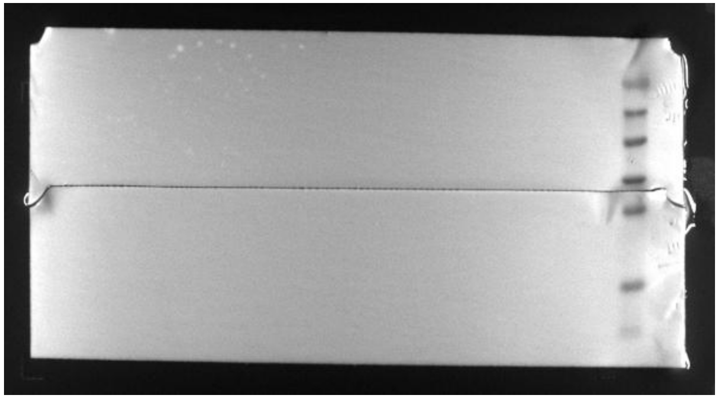

P16-actin

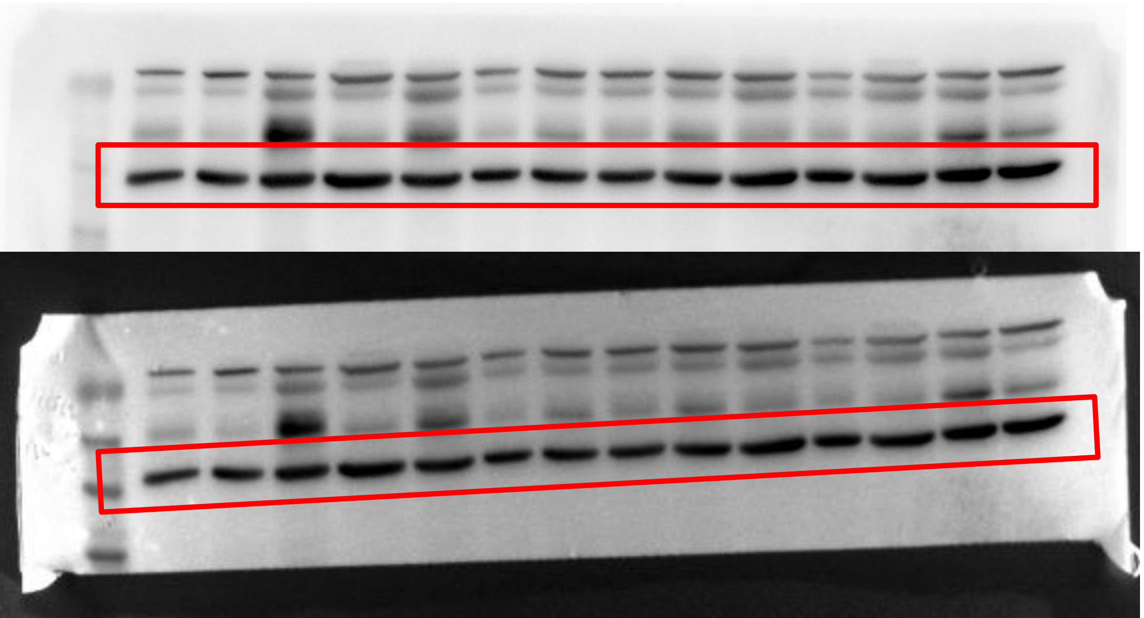

Fig6

Fn1

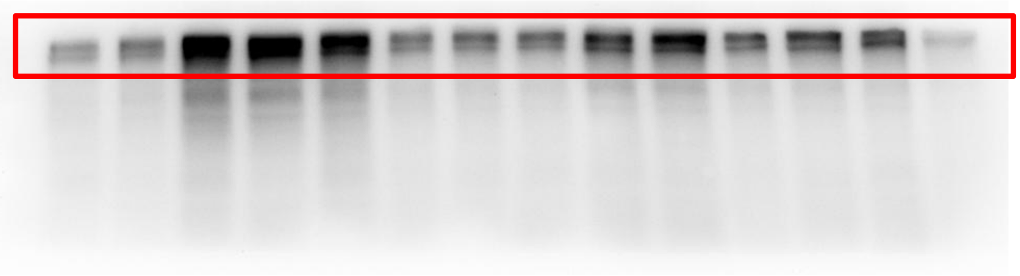

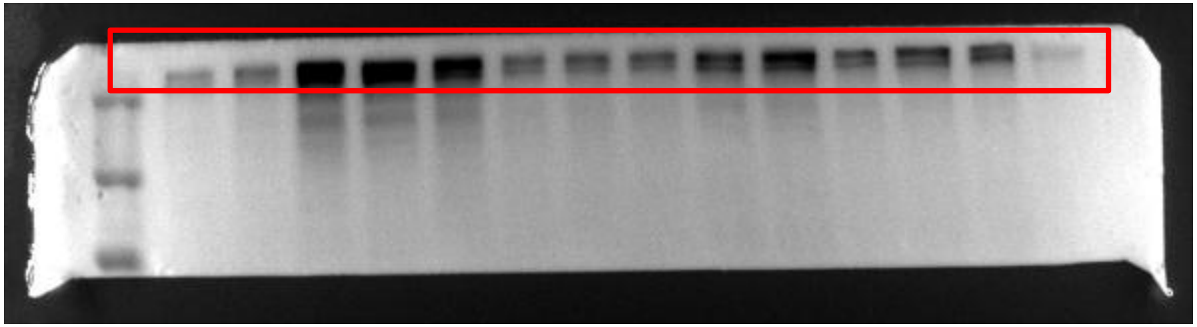

Fn1-actin

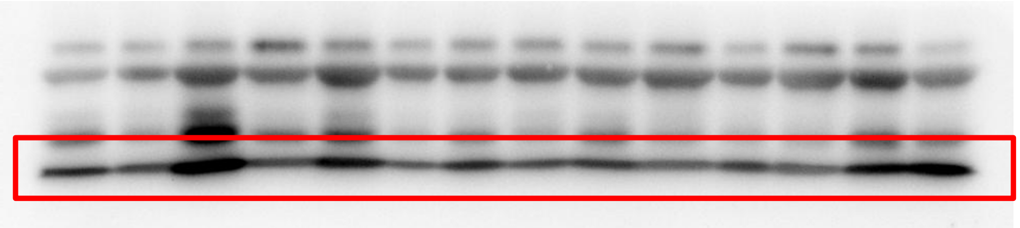

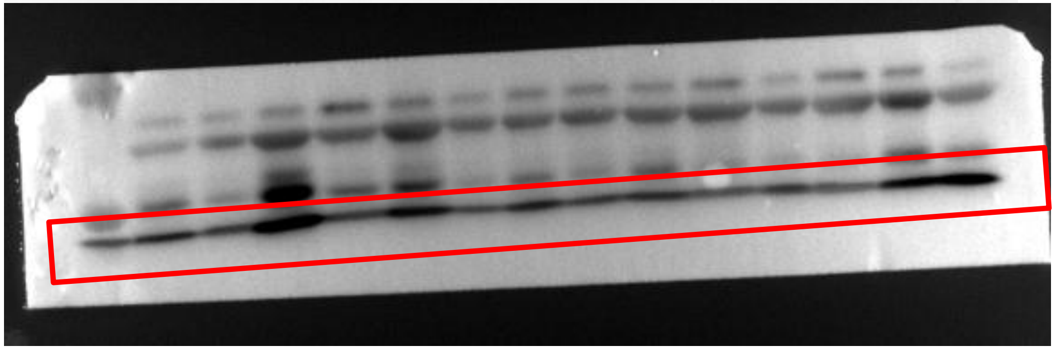

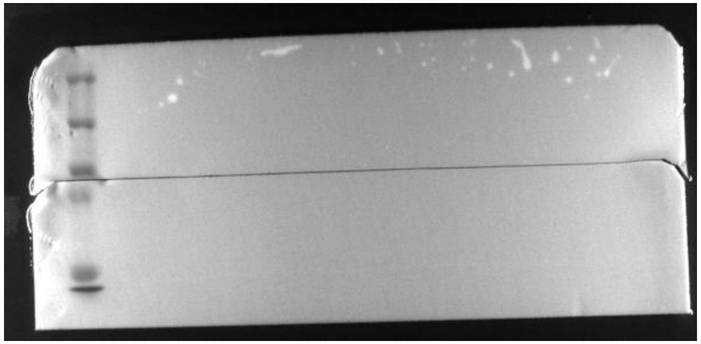

Fig6

p21

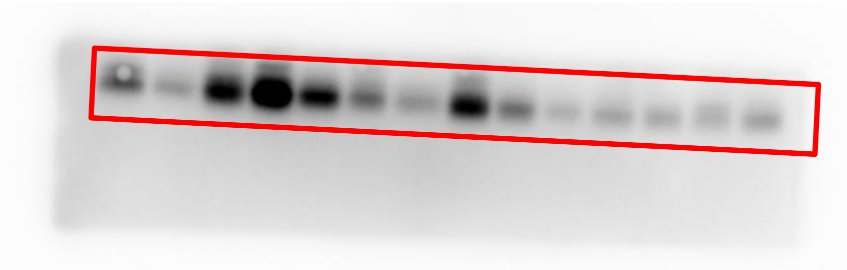

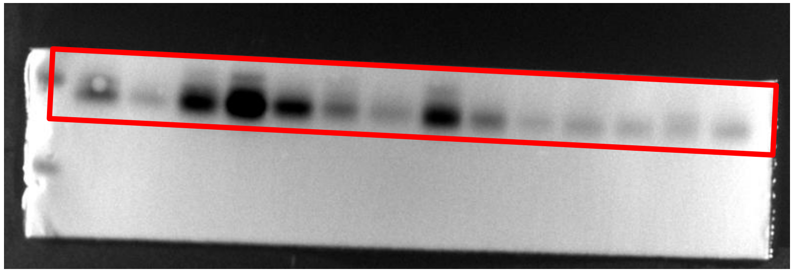

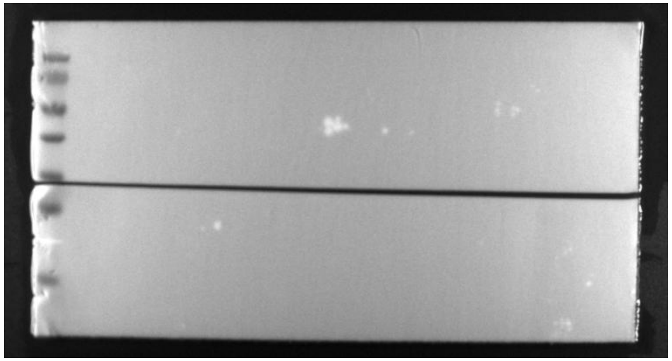

P21-actin

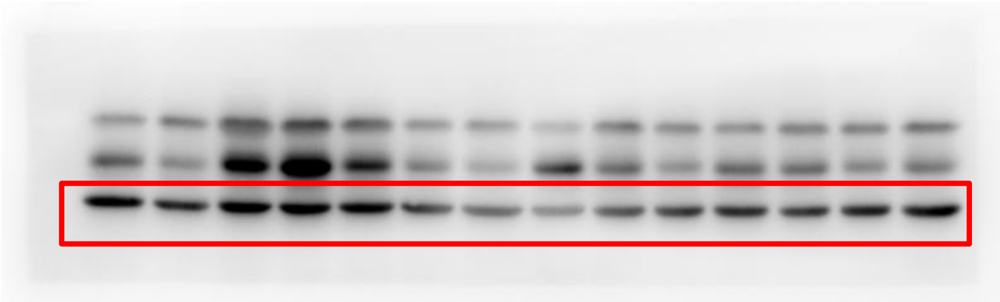

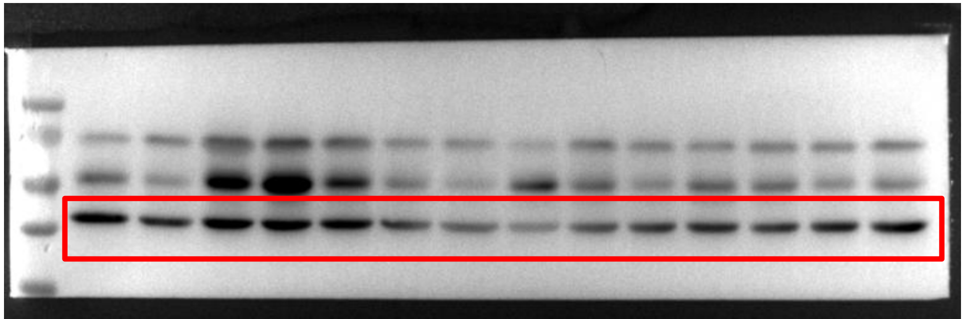

Fig6

Bcl-2

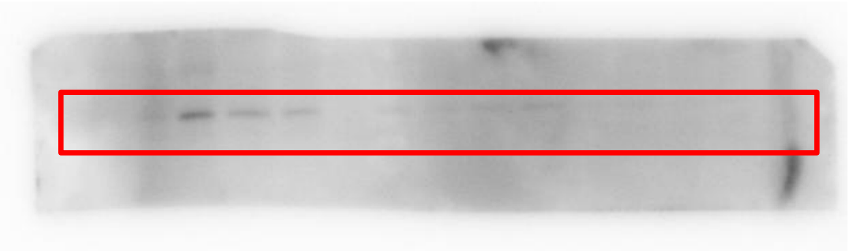

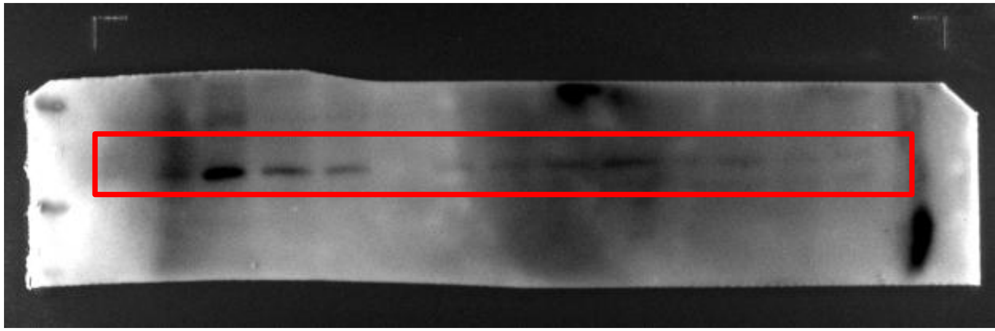

Bcl-2-actin

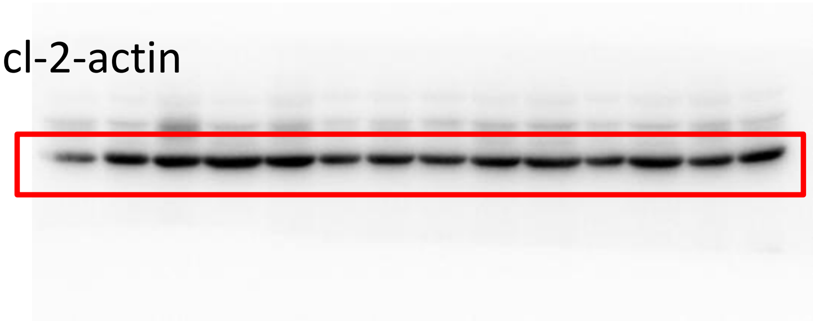

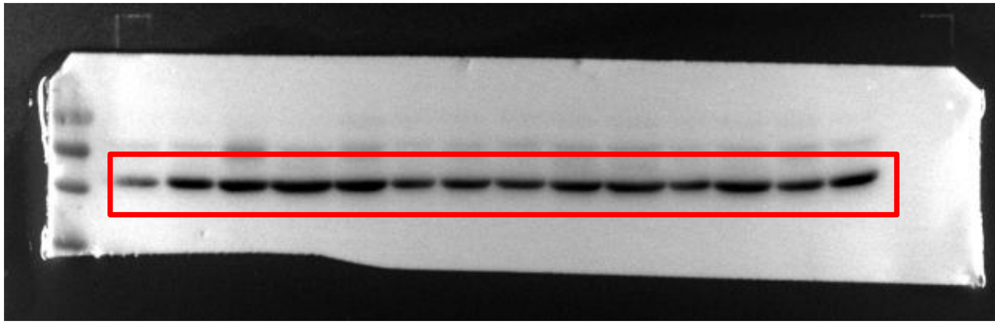

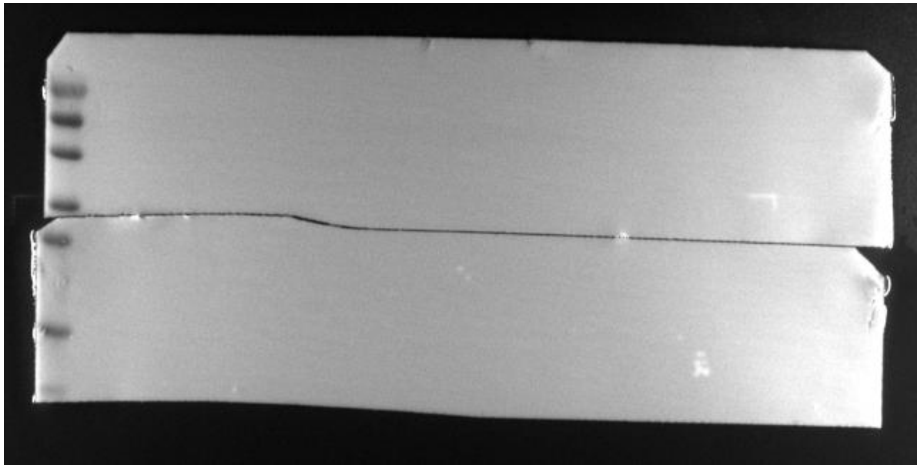

Fig7C

BAX

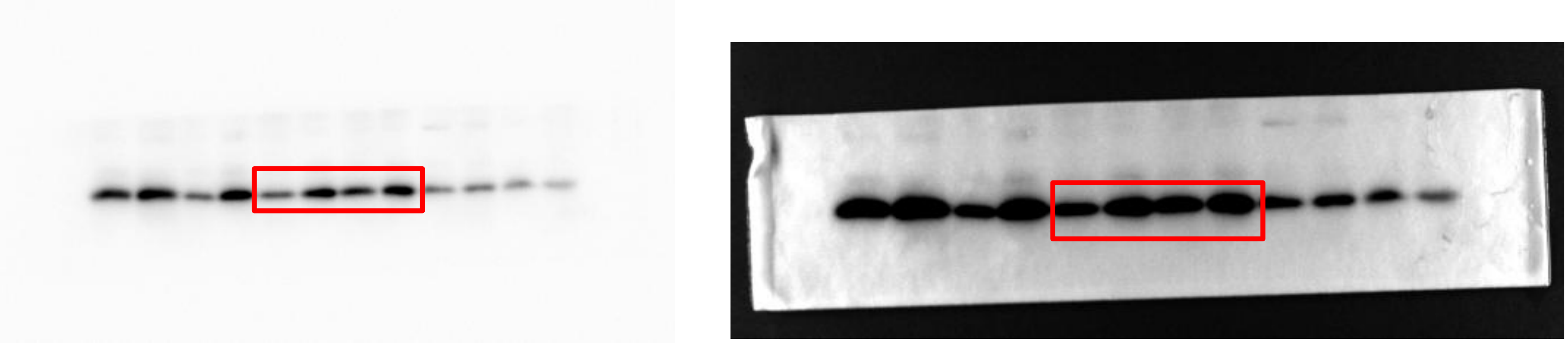

BCL2

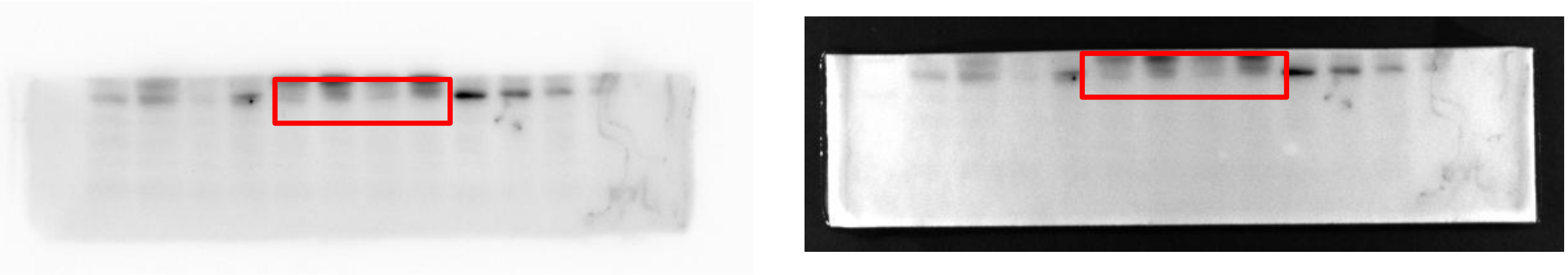

Fig7C

β-actin

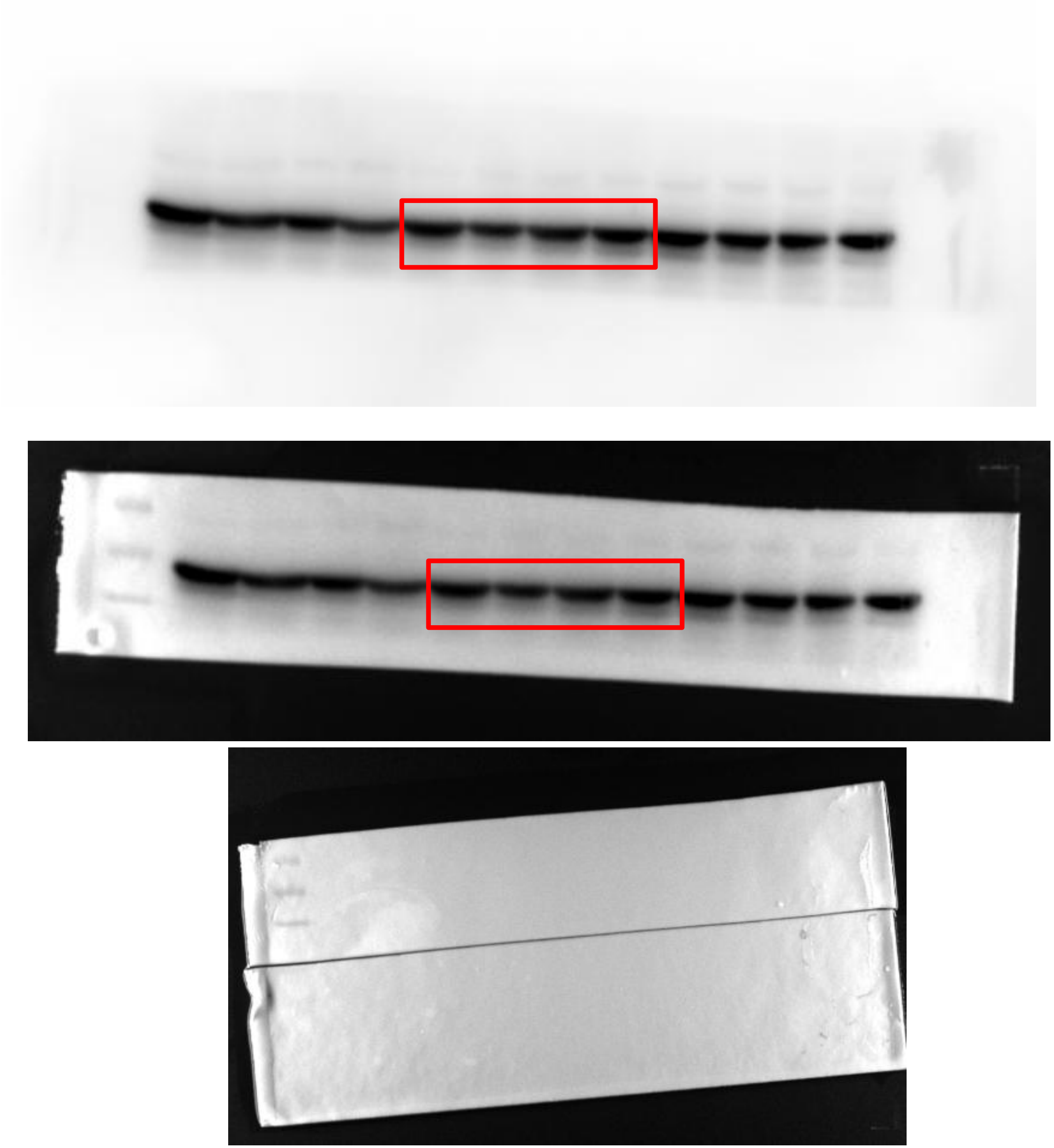

Fig7F

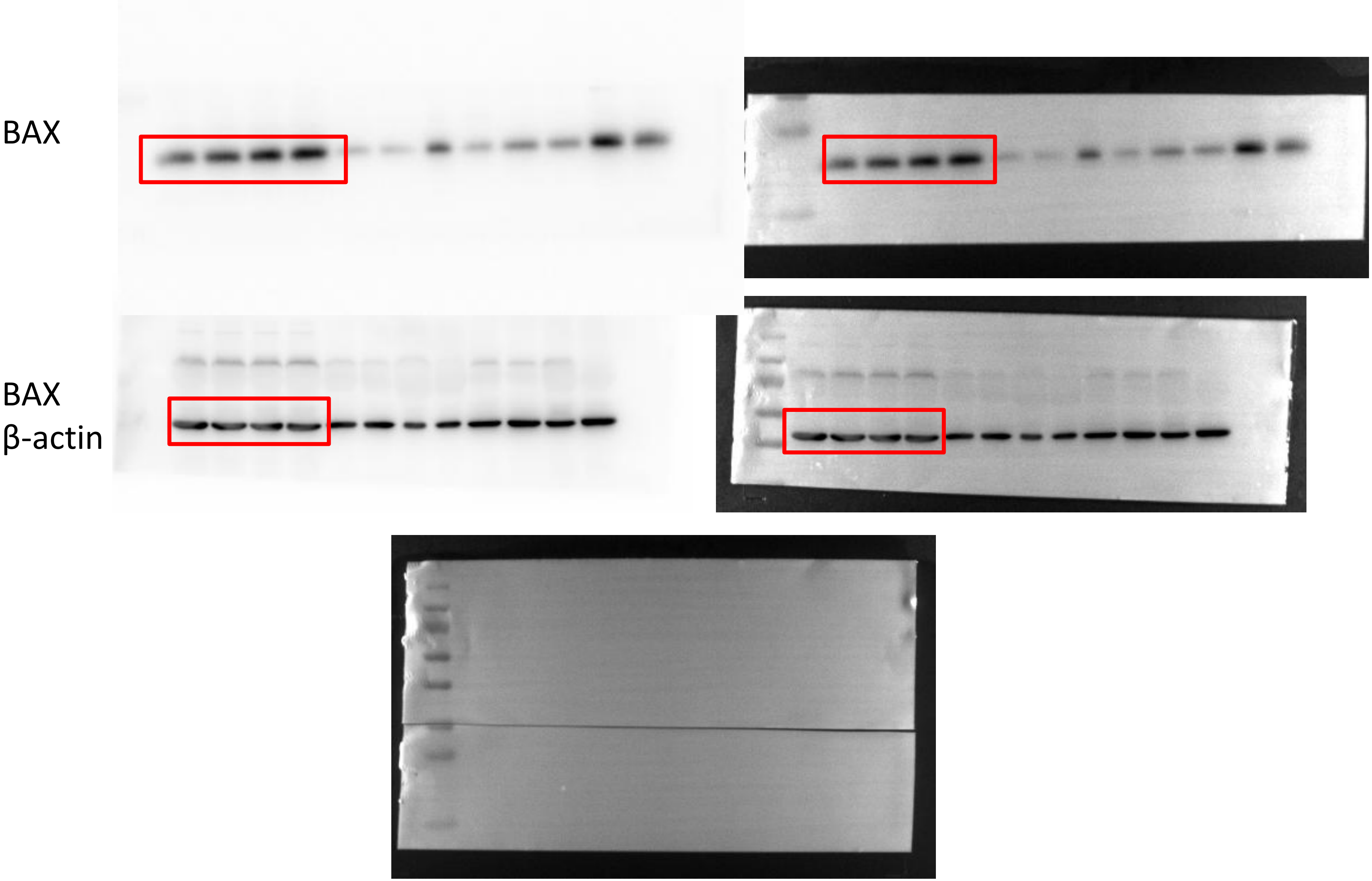

Fig7F

BCL-2

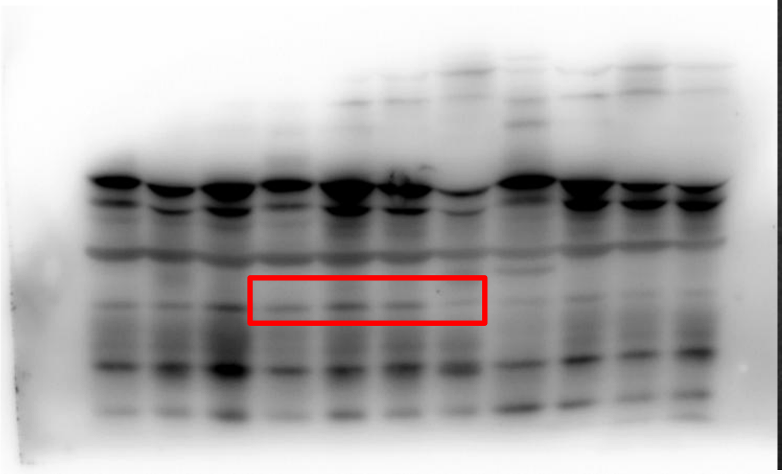

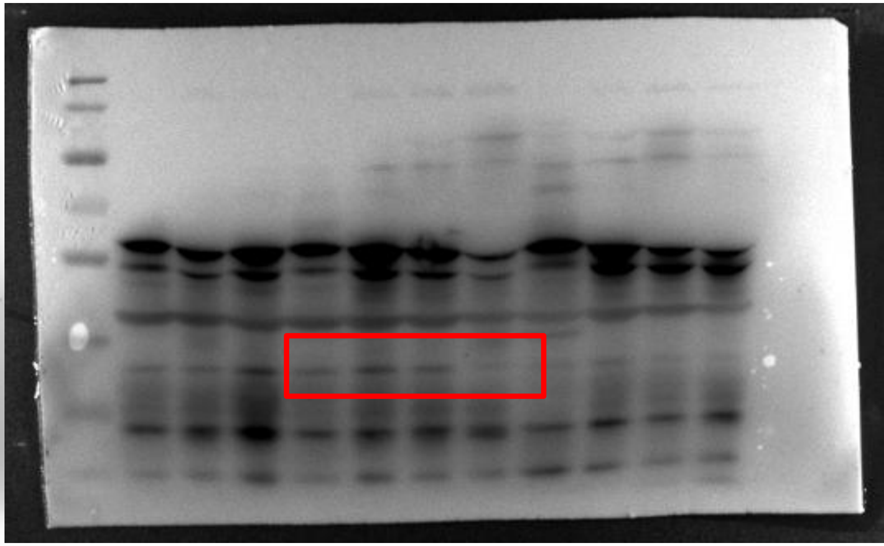

BCL-2
β-actin

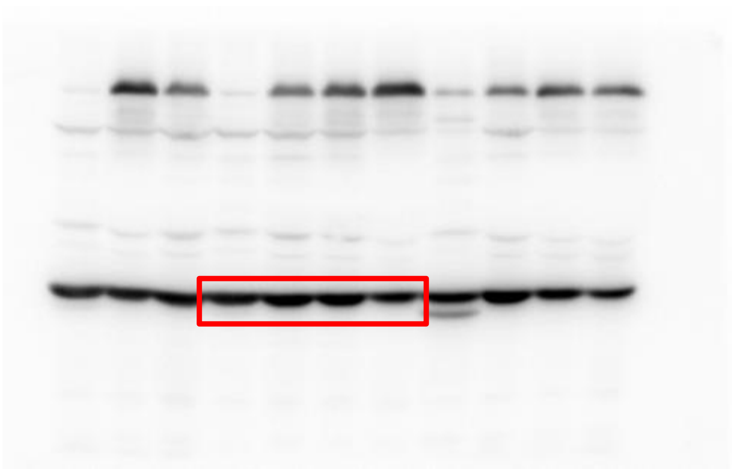

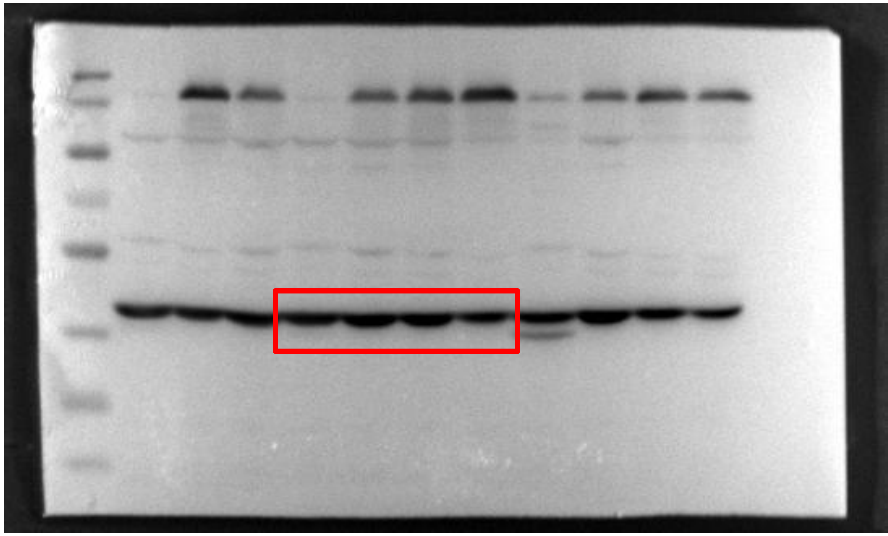

Fig7F

Survivin

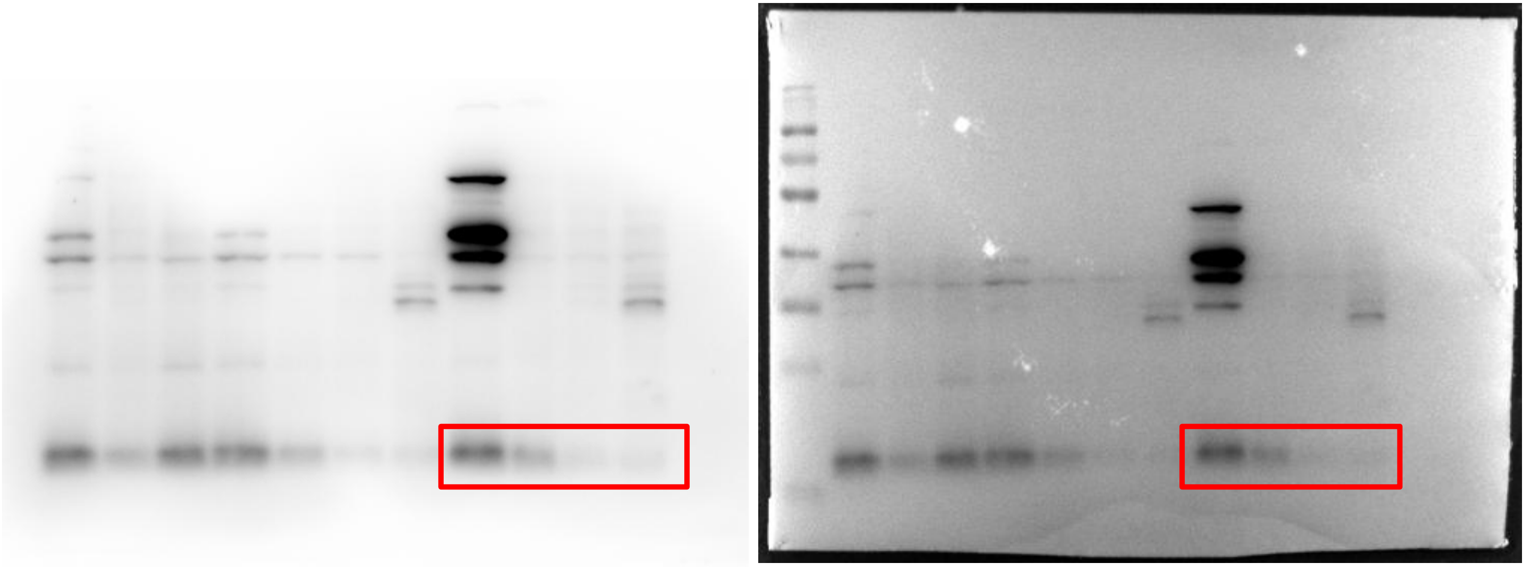

Survivin

β-actin

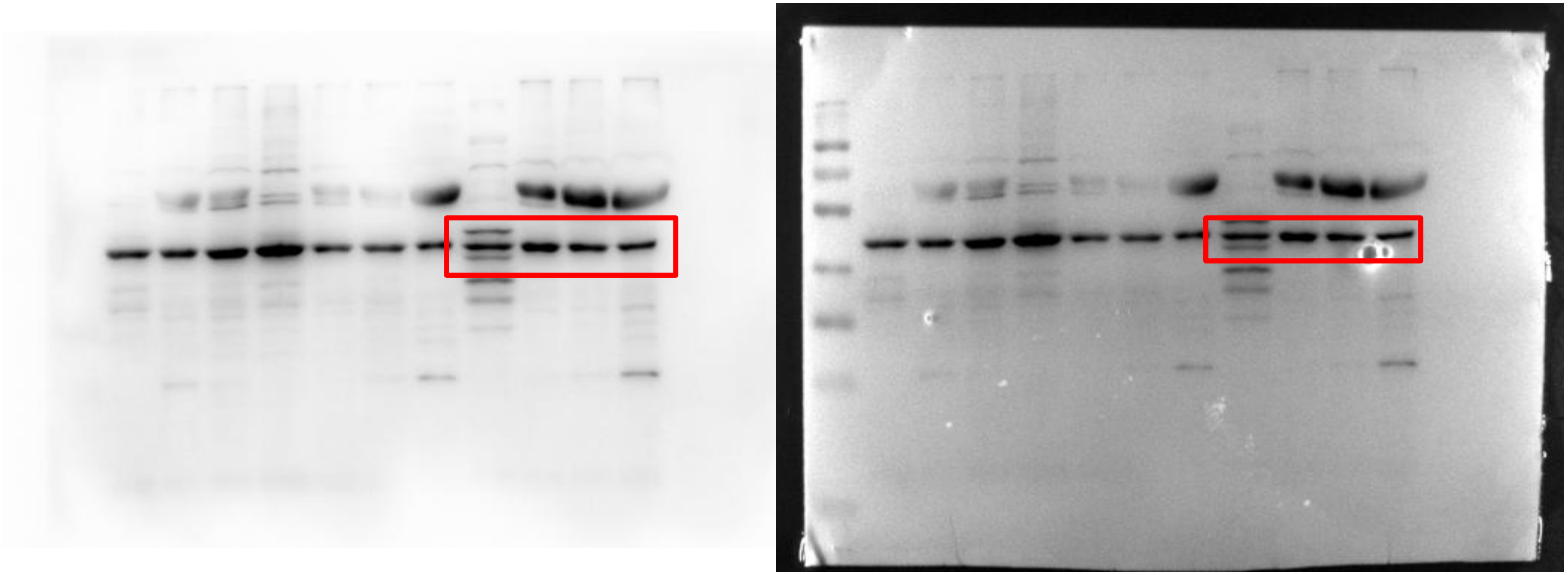

FigS1B

p16

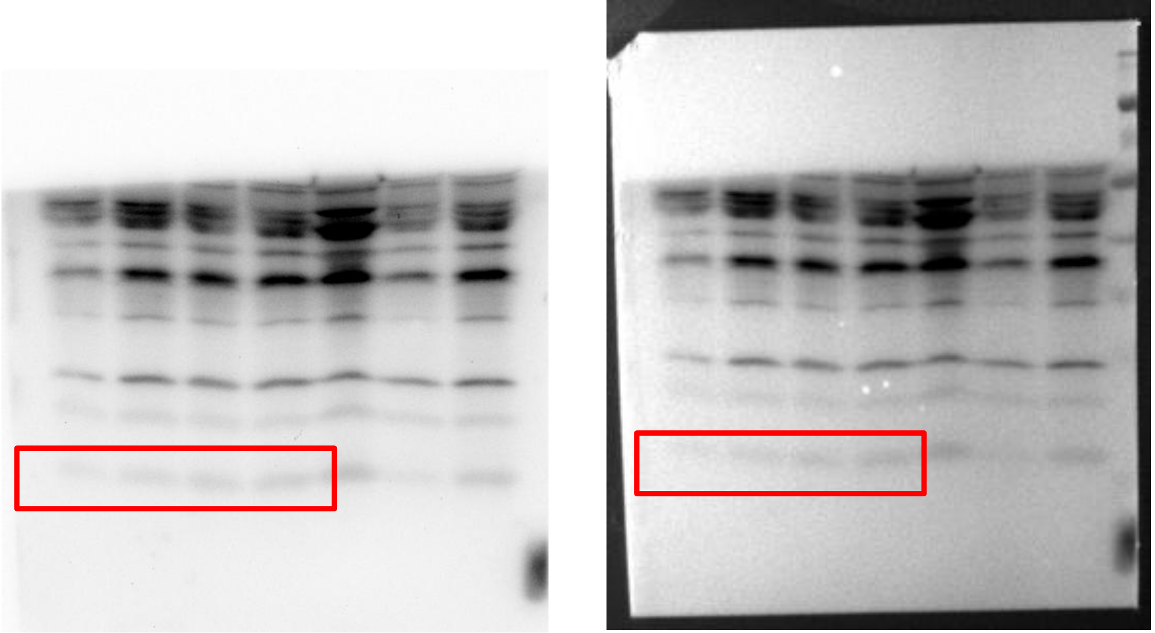

p21

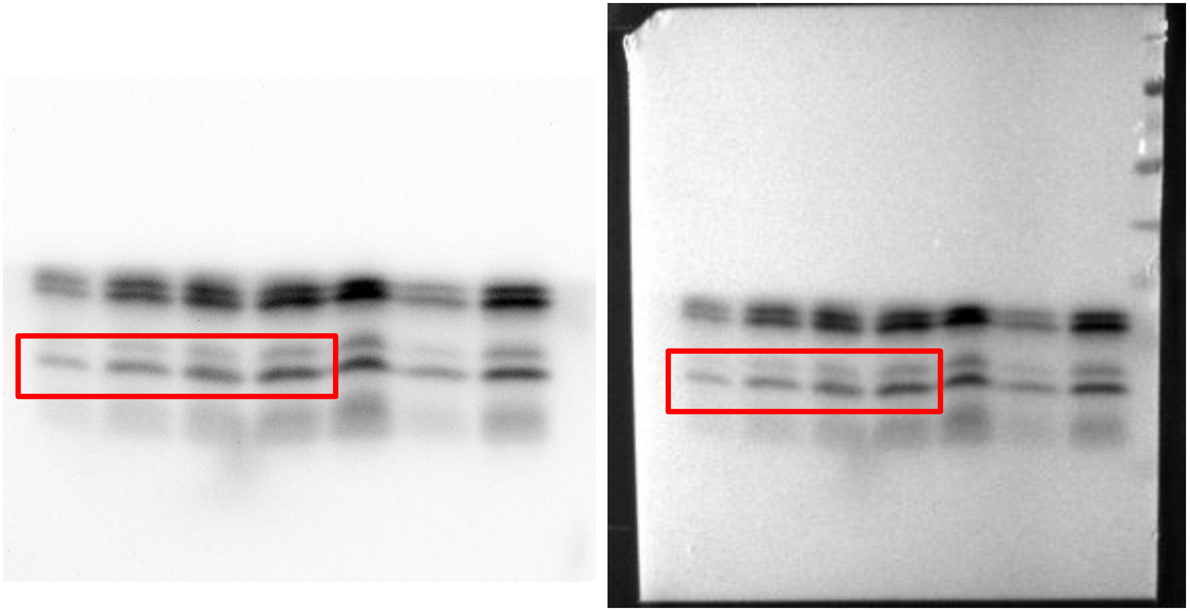

FigS1B

β-actin

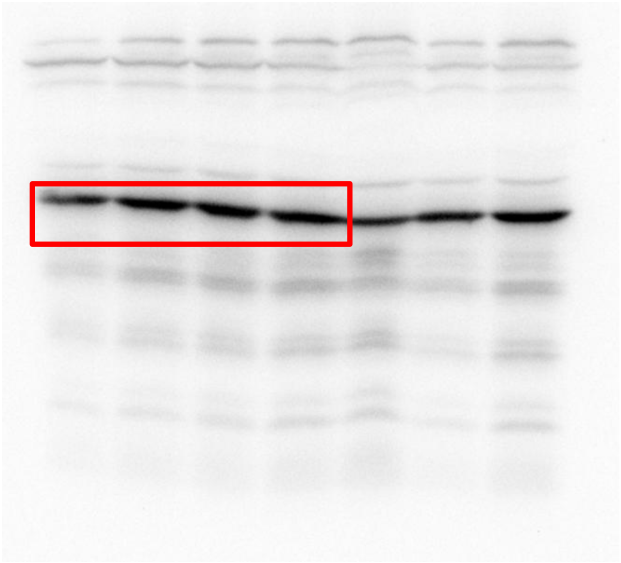

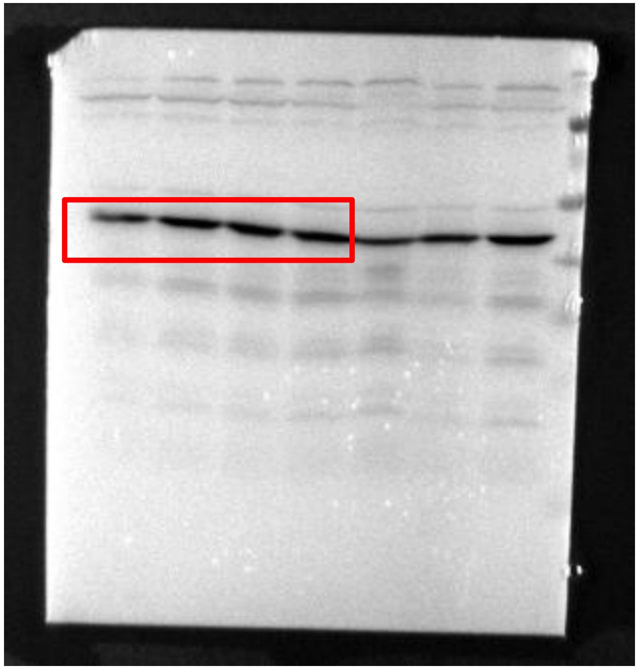

FigS1F

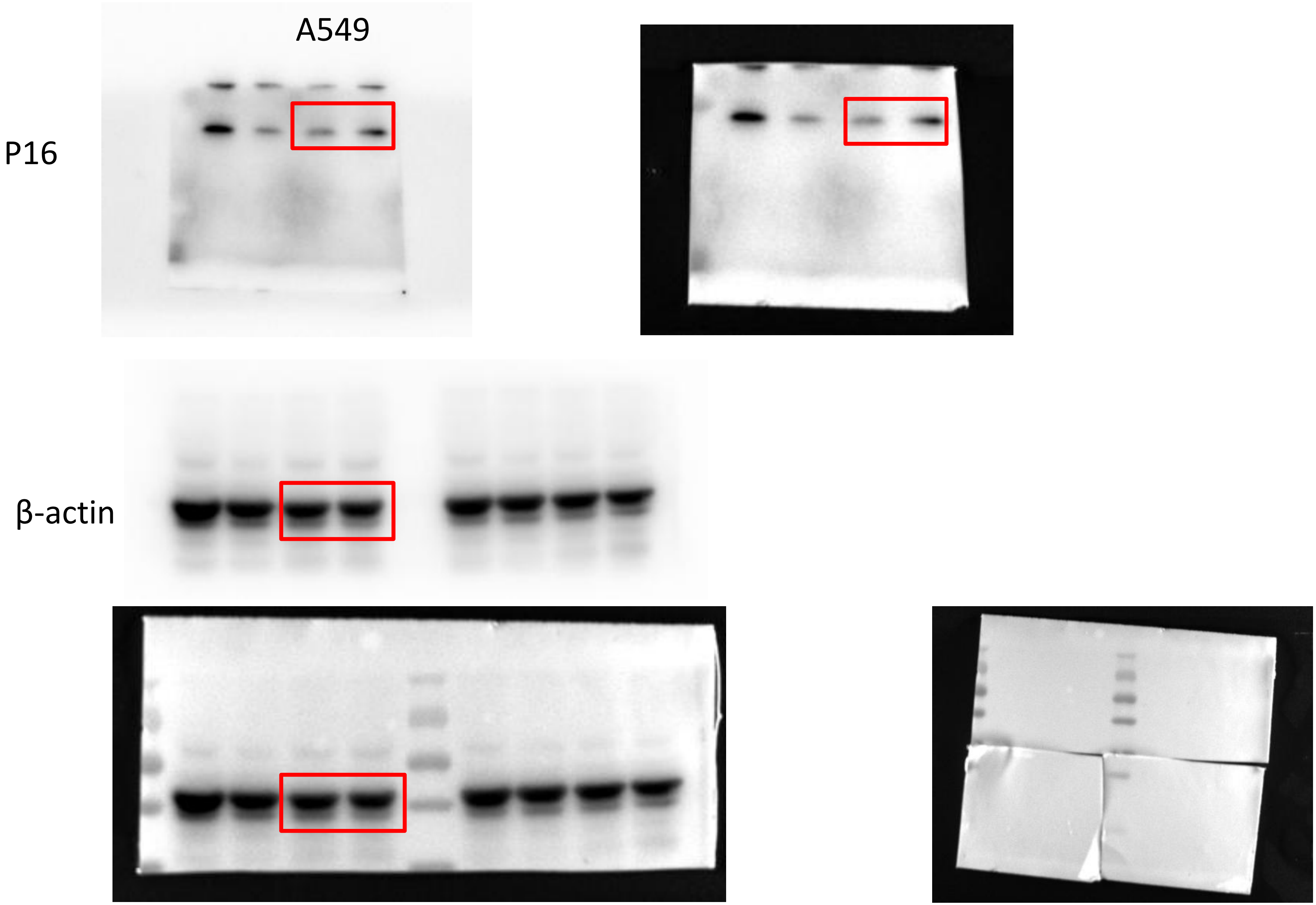

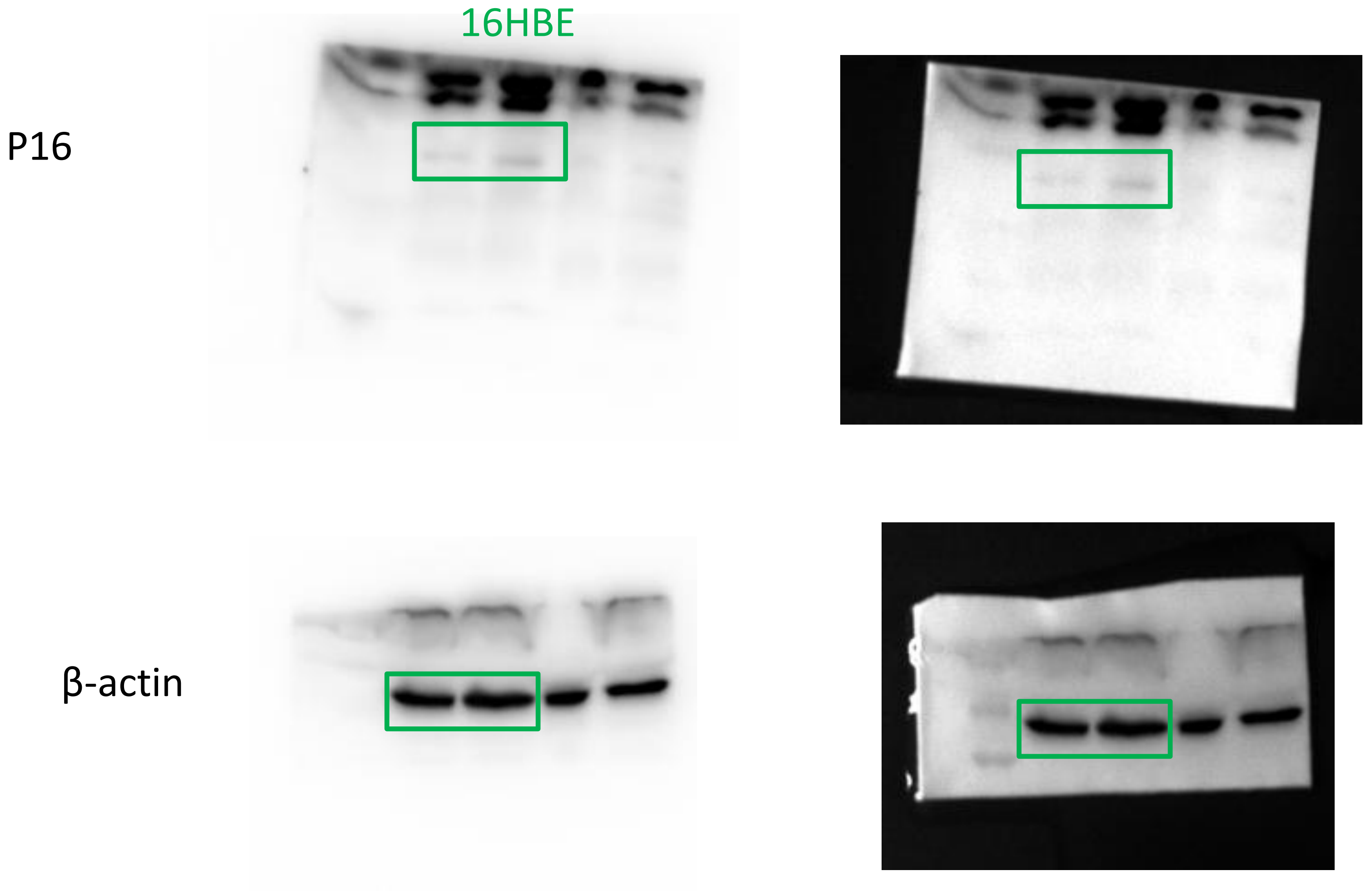
16HBE
P16
β-actin

FigS1F

P21

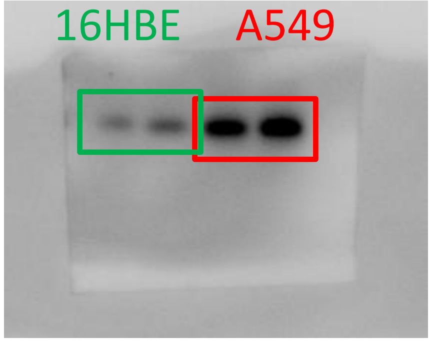

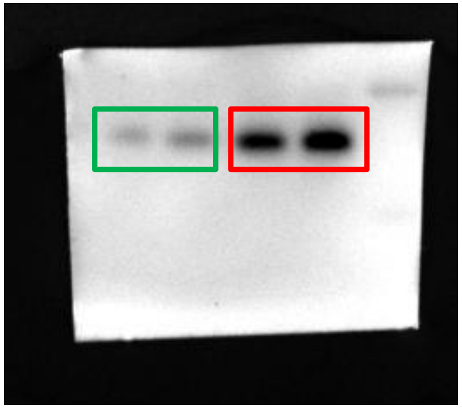

β-actin

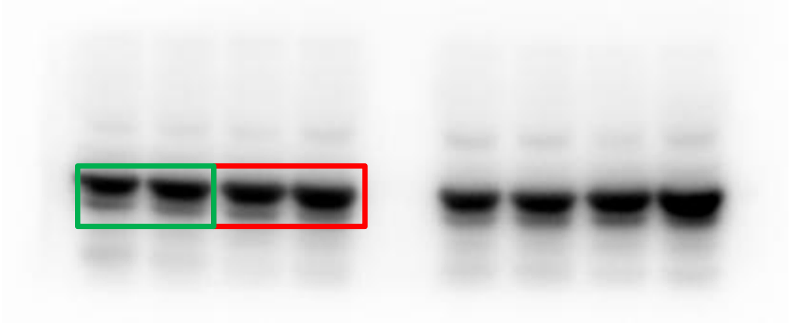

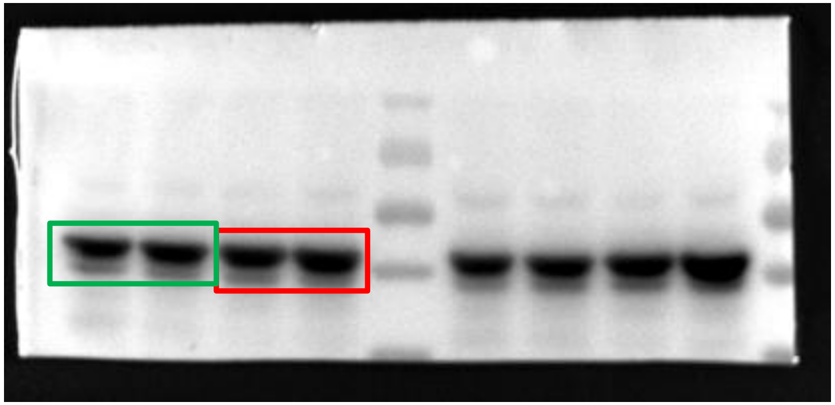

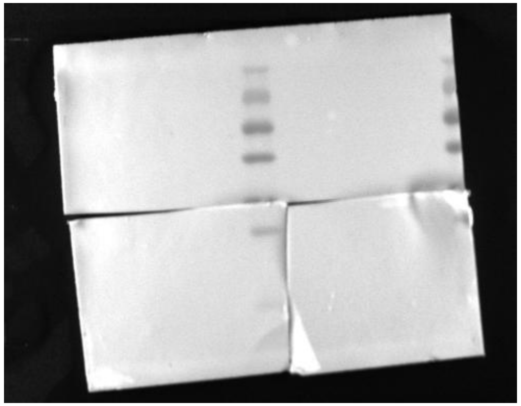

FigS2A

p16

p21

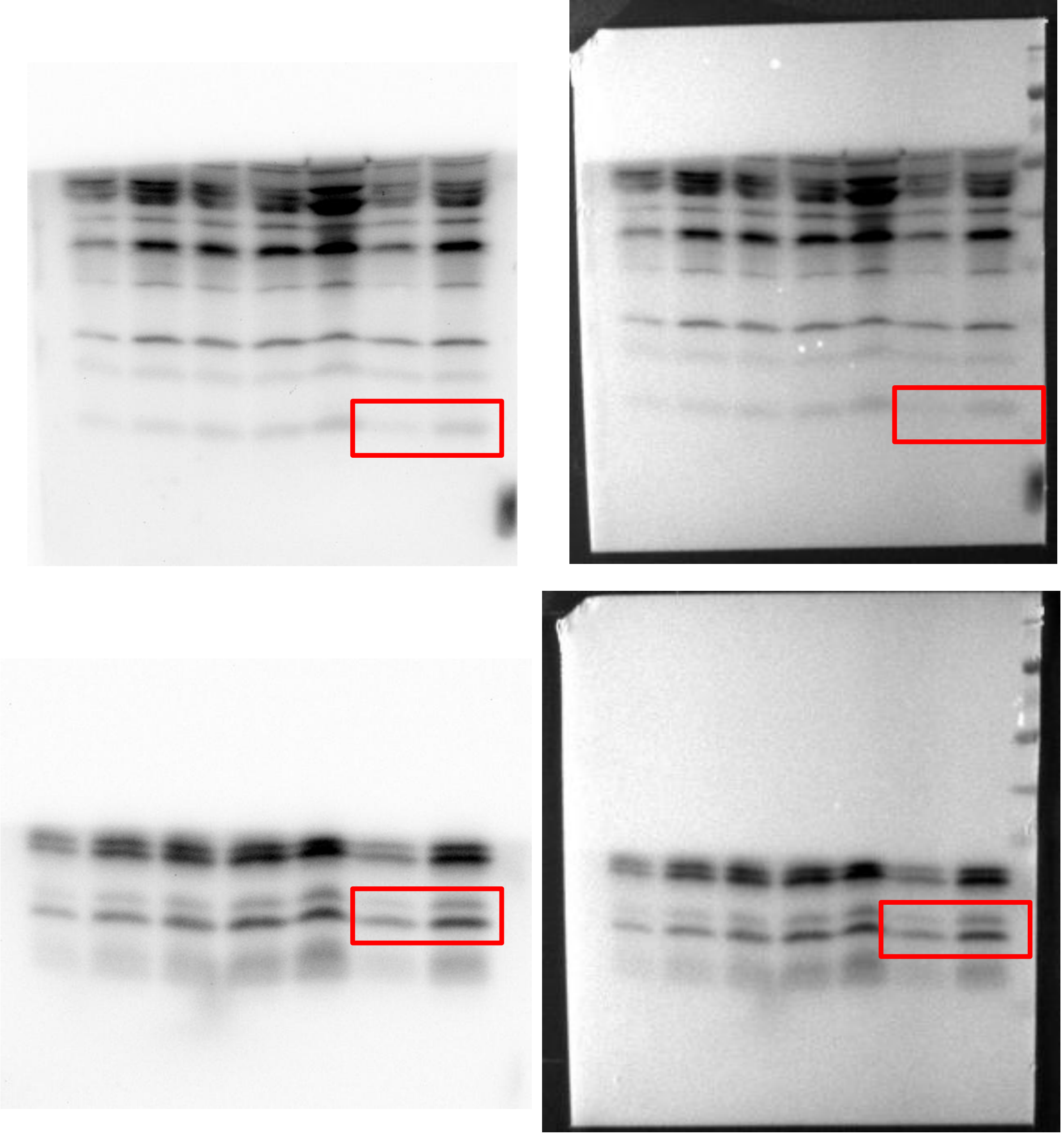

FigS2A

β-actin

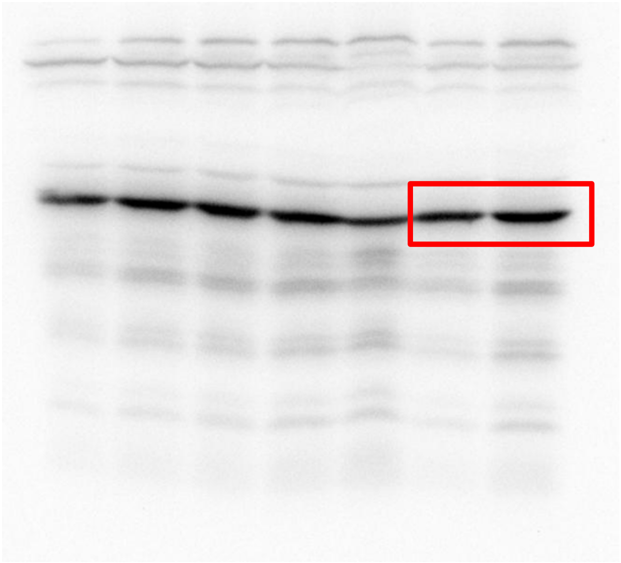

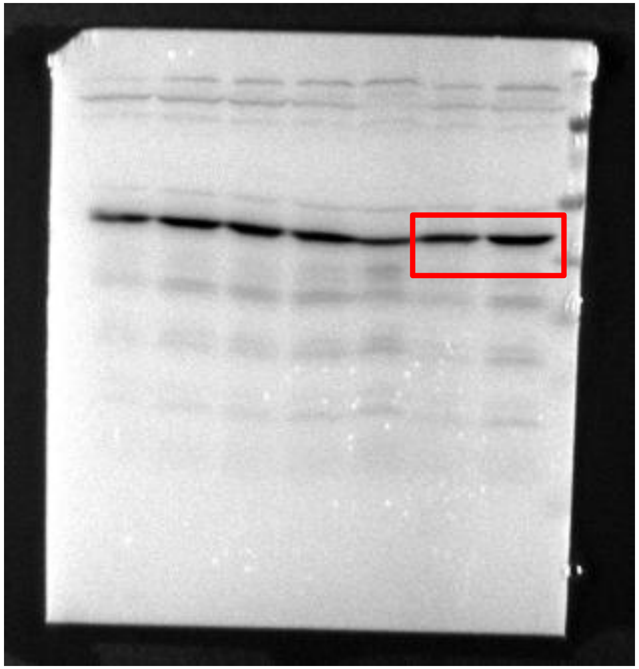

FigS2D

pYAP

pTAZ

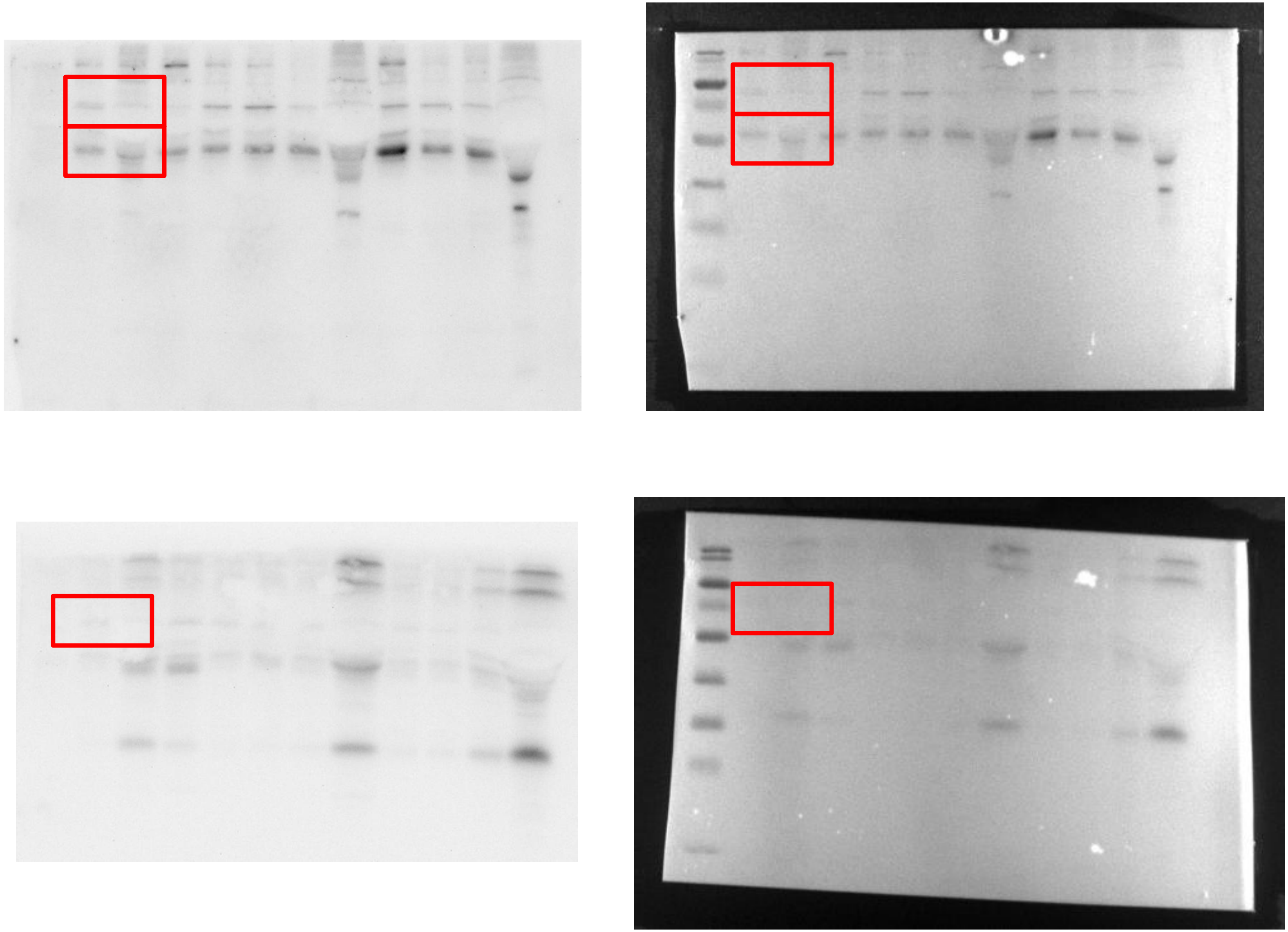

FigS2D

TAZ

β-actin

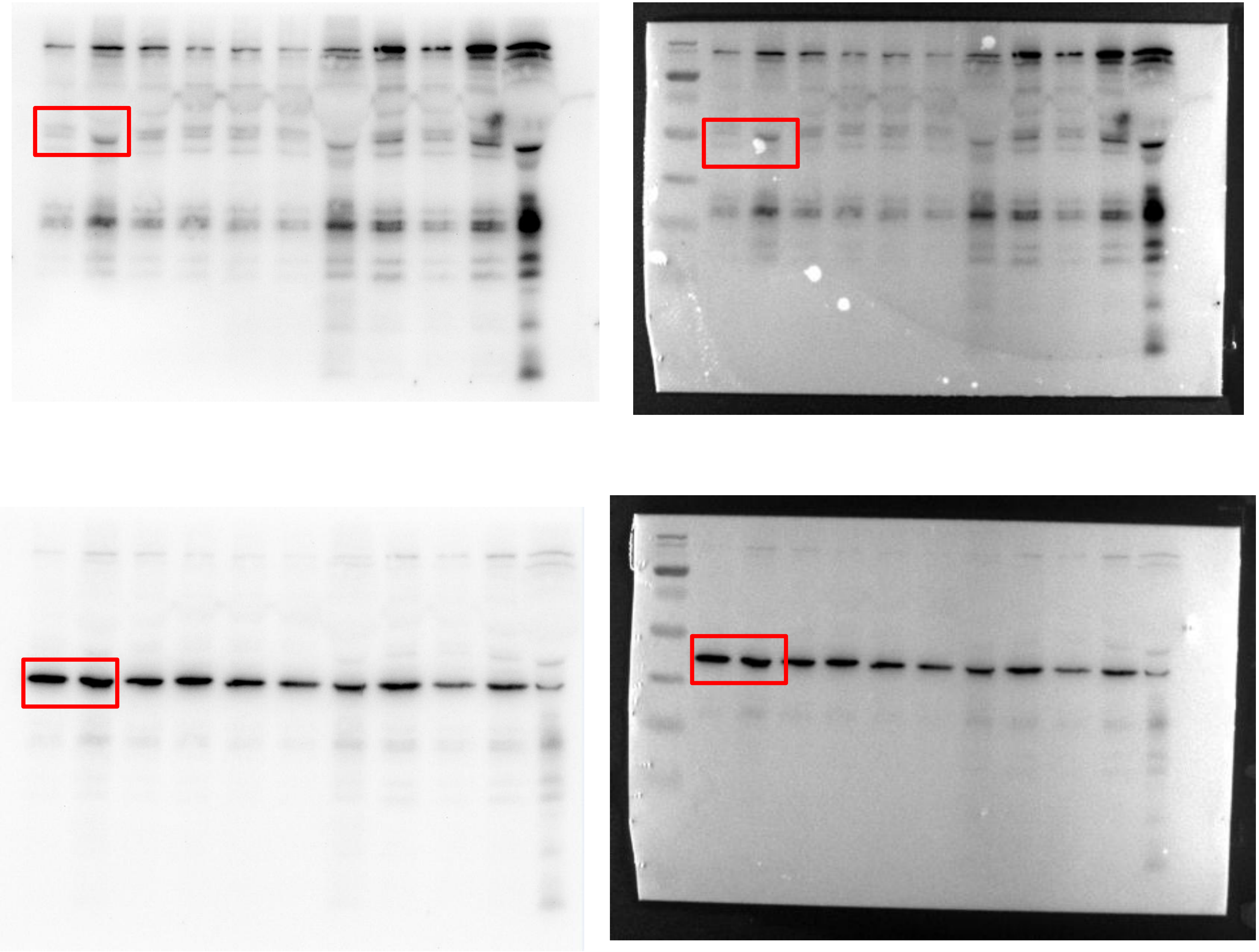

FigS2E

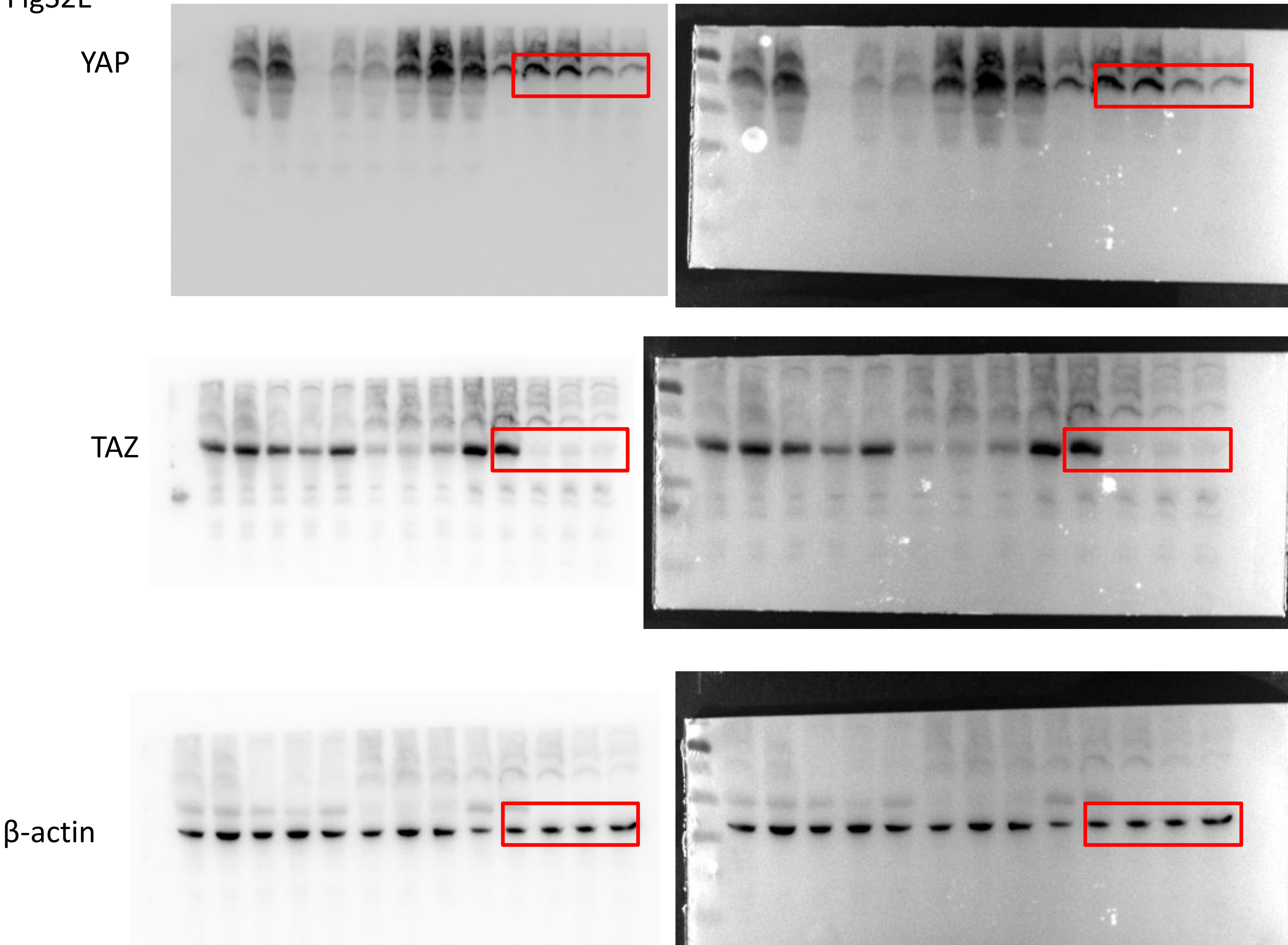

FigS2E

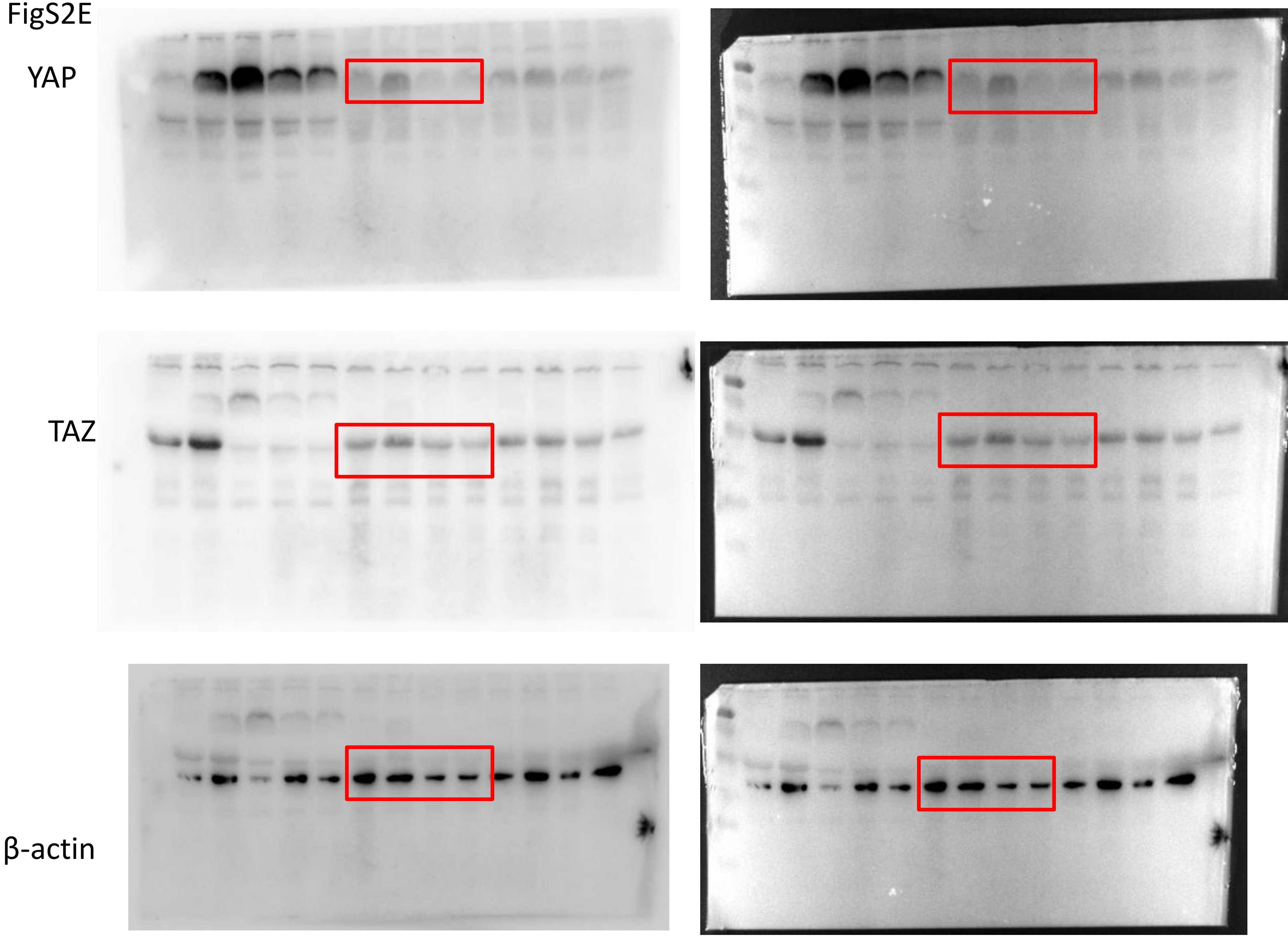

FigS2F

Yap

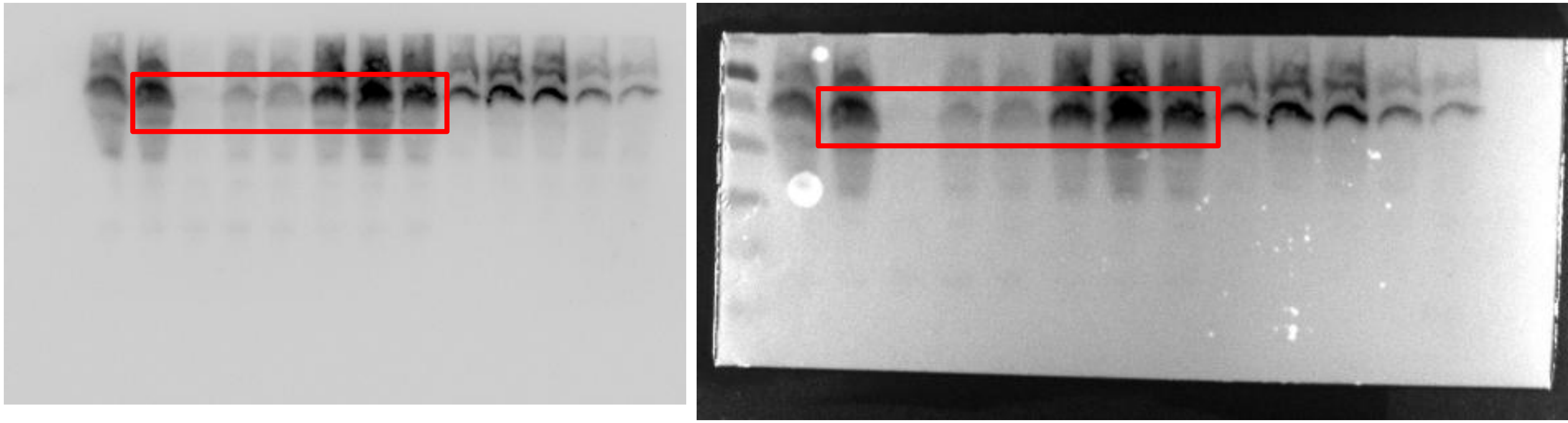

Taz

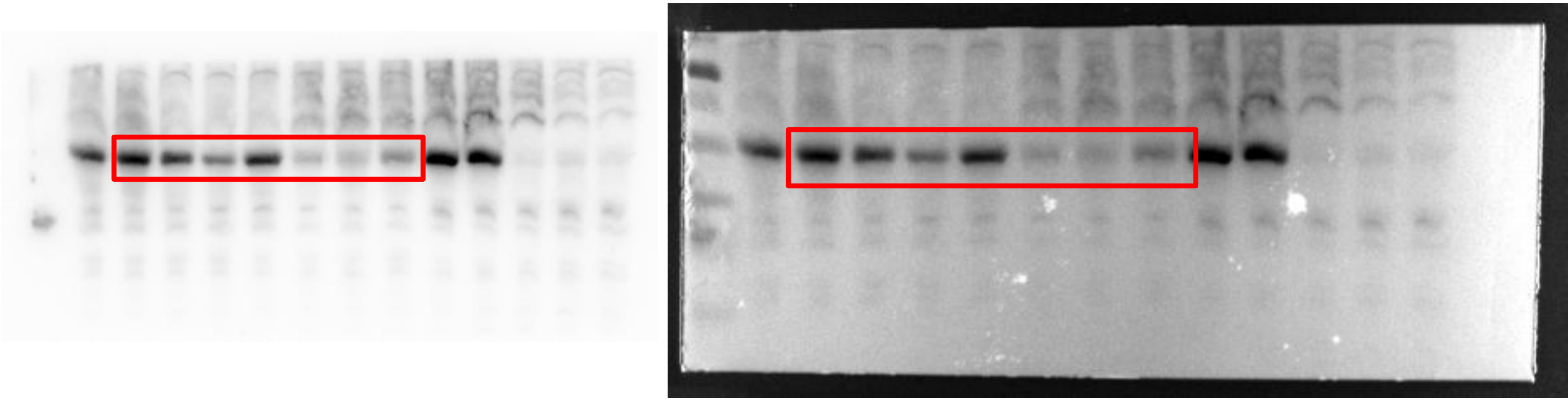

β-actin

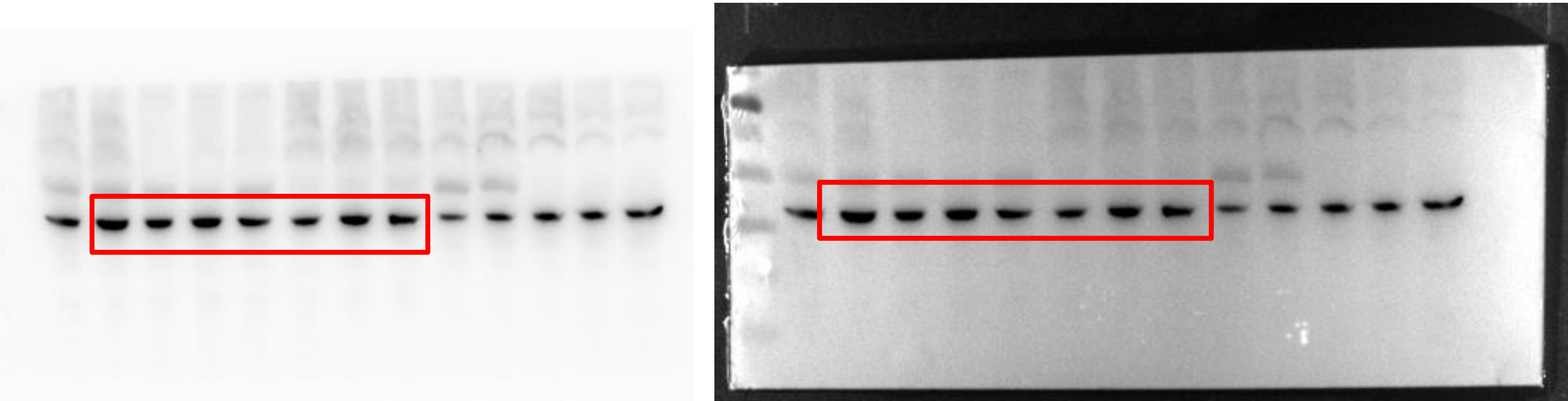

FigS2I

Yap

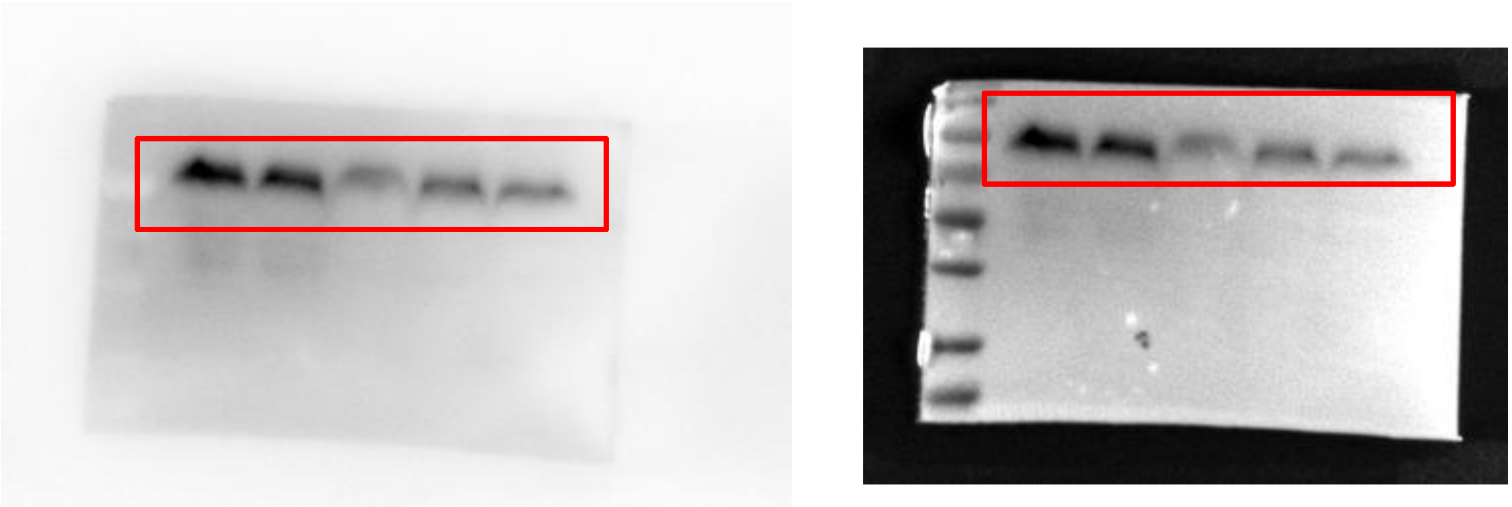

β-actin

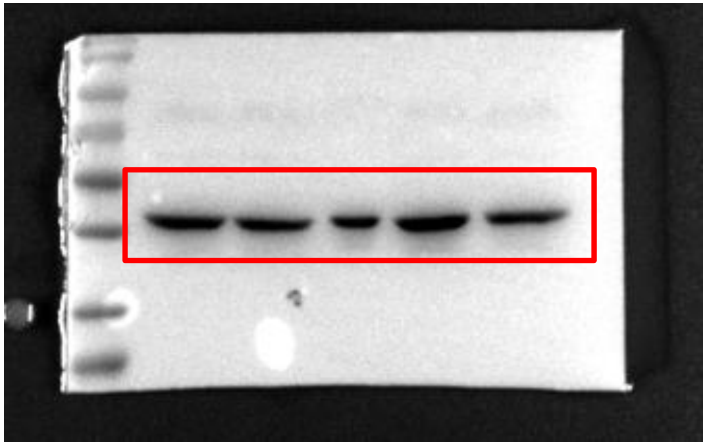

FigS2I

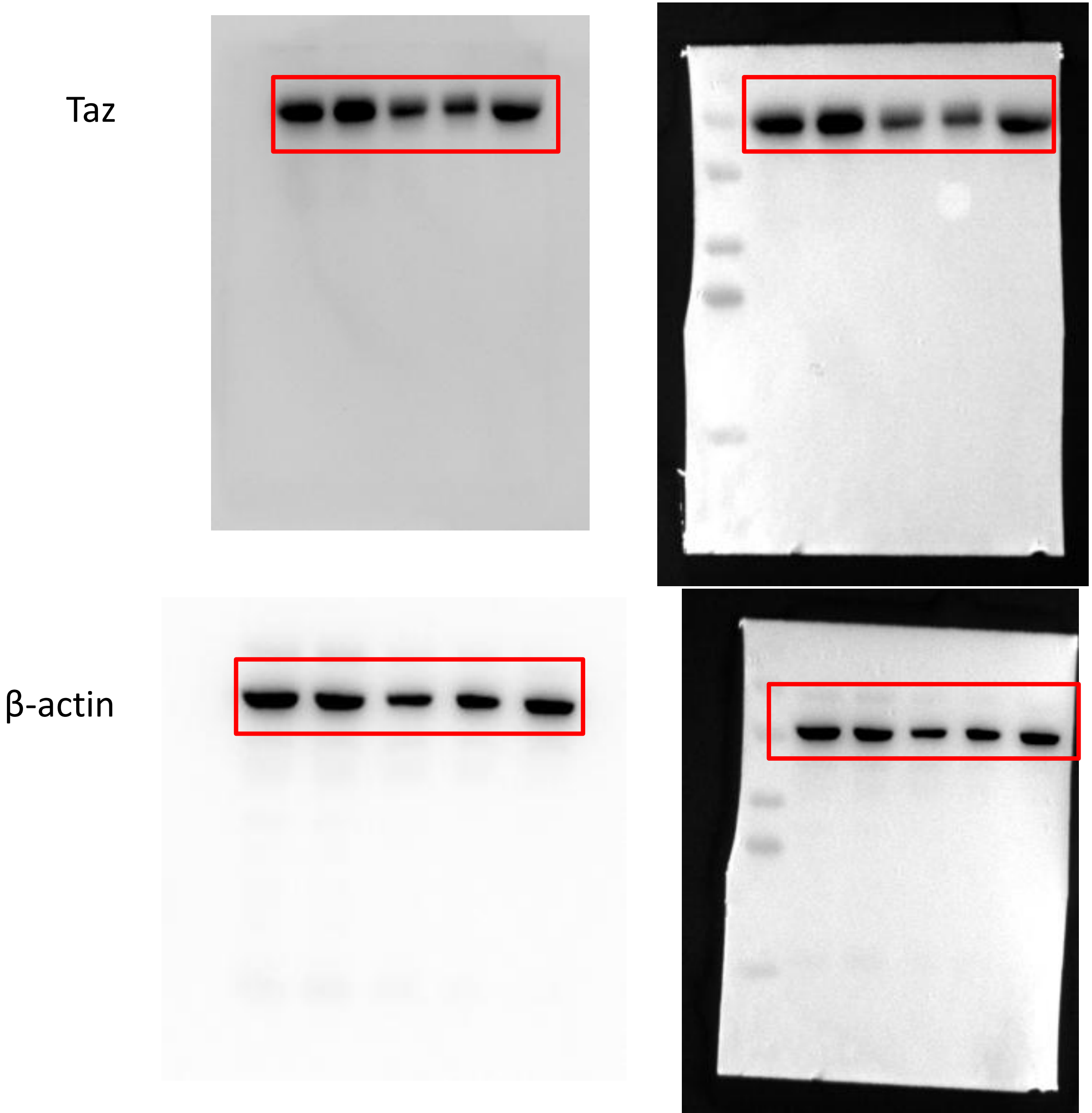

Supplement: Supplementary file 2 — Supplementary Material 2. [file 12931_2024_2832_MOESM2_ESM.pdf]
